# B(iii)-catalyzed synthesis of spirooxindole and dihydro-2-oxopyrrole under solventless conditions in a ball mill, along with DFT computations†

**DOI:** 10.1039/d5ra01991e

**Published:** 2025-07-21

**Authors:** Dina Mallah, Bi Bi Fatemeh Mirjalili, Hadi Basharnavaz, Abdolhamid Bamoniri

**Affiliations:** a Department of Chemistry, College of Science, Yazd University P.O. Box 89195-741 Yazd I. R. Iran fmirjalili@yazd.ac.ir h.basharnavaz@yazd.ac.ir +983538210644 +983531232672; b Department of Organic Chemistry, Faculty of Chemistry, University of Kashan Kashan I. R. Iran

## Abstract

Mechanochemical synthesis of heterocyclic compounds is a growing research field due to its simplicity and environmental compatibility. Solvent-free mechanochemical reactions using a ball mill not only eliminate the need for bulky solvents and reduce waste but also open the door to the synthesis of various organic compounds, including common drugs. Combining two different acids, including molybdic acid (MoO_3_(H_2_O)_3_ complex structure) and Lewis acid BF_3_, is a smart strategy to prepare a new and porous cluster nano-catalyst with high acidic properties (pH = 1). Here, the MoO_3_/BF_3_ nano-catalyst was prepared. This heterogeneous acidic nano-catalyst was then investigated for the synthesis of derivatives of nitrogen-containing heterocyclic compounds, such as spirooxindoles and dihydro-2-oxopyrroles, using the Michael–Mannich cyclocondensation method under solvent-free mixer Mill conditions. Which is, a green and environmentally friendly method. These results align with the data obtained from the DFT calculations. Total energy and electronic band gap energy (*E*_g_ = *E*_HOMO_ − *E*_LUMO_) calculations were performed for all dihydro-2-oxopyrrole and spirooxindoles derivatives synthesized in this work. The type of final structure of the catalyst was determined using different analyses such as analyses FT-IR, XRD, FESEM, EDX, EDS-MAP, TEM, BET, and TGA were performed.

## Abstract 2

Mechanochemical synthesis of heterocyclic compounds is a growing research field due to its simplicity and environmental compatibility.

## Graphical abstract

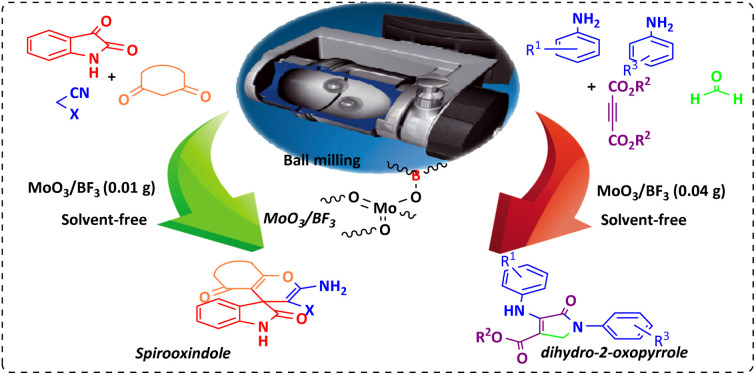

## Introduction

In recent years, due to the occurrence of serious environmental problems caused by solvents and chemicals, green chemistry has confirmed the need to find suitable alternatives, so the ball mill is known as a useful tool for the synthesis of organic compounds. The synthesis of heterocyclic compounds using advanced mechanochemical and environmentally friendly processes has advantages compared to ultrasound, microwave, and solvent-based methods.^1–4^

Recently, heterocyclic compounds, especially nitrogen-containing compounds, form many important organic compounds for natural products, agrochemicals, and pharmaceuticals. For this reason, the formation of compounds containing C–N bonds has been widely investigated in the past decades. Typically, the synthesis of this class of compounds requires significant amounts of solvent, and in most cases, relatively harsh reaction conditions are employed.^5–7^

**Scheme 1:**
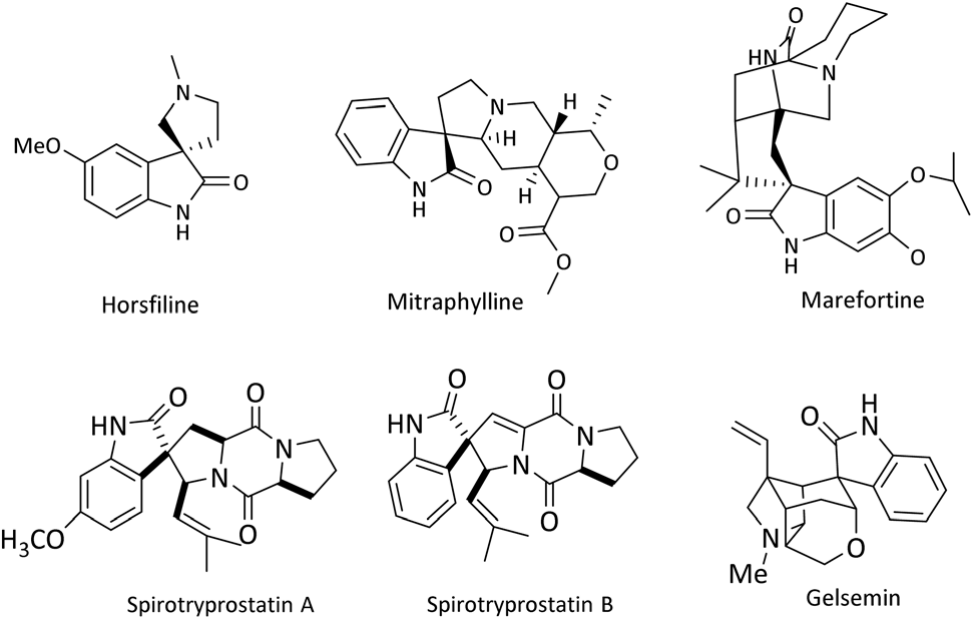
The structure of some pharmacologically and biologically active heterocyclic compounds containing spirooxindole.

Due to their rigidity and three-dimensional geometric structure, spirocyclic compounds occupy a unique position in organic chemical compounds. The existence of spirocycles was first proposed by von Baeyer in 1900.^8^ Among all spirocyclic compounds, spirooxindole compounds are an important branch of this category. Spirooxoindoles contain a fused spiro ring at C2 or C3 of the oxindole part, they are also a well-known subgroup of indole and form the core of very functional organic structures.^9,10^ These important structures are known as the central skeleton of many alkaloids with medicinal activity such as Horsfiline,^11^ Mitraphylline,^12^ and Marefortine.^9^ Spirooxindole compounds are found abundantly in nature have anti-fungal, anti-microbial, and anti-tumor properties, and are useful as an anti-cancer drug in cancer treatment.^13–15^ Two examples of spiro compounds in nature that have a spirooxindole system are Spirotriprostatin A and Spirotriprostatin B, which are obtained from *Aspergillus fumigatus* mold fermentation in a liquid medium and have anti-mitotic and inhibitory properties in the mammalian cell cycle.^16^ Another example of this class of spiro substances is an alkaloid isolated from the native jasmine flower of Guatemala, called Gelsemine, it is used as a topical medicine to treat muscular rheumatism, tonsillitis, inflammation of the esophagus, headache, and earache.^17^ Rhynchophylline is an antihypertensive, anticonvulsant, headache, non-antagonist. Horsfiline is used in traditional medicine and Mitraphylline has an anti-tumor activity for human brain cells and neuroblasts^14^ (Scheme 1).

One of the most common methods for the synthesis of compounds with spirooxindole skeleton is the use of isatin compound, considering the pharmacological and medicinal importance of this class of compounds, significant efforts have been made to synthesize spirooxindole compounds, one of the most efficient of which includes The three-component reaction is isatin, malononitrile and compounds with enolizable active C–H such as dimedone, 2-hydroxyphthalene-4,1-dione, and 4-hydroxycoumarin 18. So far, the derivatives of spirooxindoles have been synthesized using many catalysts and different conditions such as Rh_2_(OAc)_4_, chiral phosphoric acid (R),^19^ urea/ChCl,^20^ piperidine,^21^ Cu(CH_3_CN)_4_PF_6_,^22^ SiO_2_@g-C_3_N_4_,^23^ Fe_3_O_4_/GO/Au-Ag,^24^ CaFe_2_O_4_@MgAl-LDH,^25^ NiO@g-C_3_N_4_,^26^ CoFe_2_O_4_@SiO_2_,^27^ GN/SO_3_H,^28^ Au.^29^

Oxopyrrole rings are biologically and pharmacologically important as vital structural parts of natural and unnatural products. Holomycin and thiolutin,^30^ thiomarinol A4,^31^ and oteromycin^32^ are some natural bioactive molecules with oxopyrrole rings. In addition, this compound exhibits biological properties such as antitumor,^33^ herbicide,^34^ and pesticide^35^ activities, as well as compounds containing oxopyrroles as platelet aggregation inhibitors^36^ cardiac cyclic AMP phosphodiesterase inhibitors,^37^ endothelial growth factor receptor^38^ has been reported.

Several methods have been reported for the synthesis of multi-functional dihydro-2-oxopyrroles, the best known Michael–Mannich cyclocondensation reaction method is used, which uses amine, dialkyl acetylene dicarboxylate (DAAD), and formaldehyde.^39^ Some catalysts that have been reported for the synthesis of this class of compounds are listed here such as 2,6-pyridinedicarboxylic acid,^40^ 4CzIPN,^41^ trifluoroacetic acid,^42^ caffeine,^43^ salicylic acid,^44^ TiCl_4_/nano-sawdust,^45^ glycine,^46^ Fe/MWCNTs,^47^ I_2_,^48^ A_l_(H_2_PO_4_)_3_,^49^ H_3_PW_12_O_40_/Fe_3_O_4_@SiO_2_-Pr-Pi,^50^ nano-Fe_3_O_4_@ SiO_2_/SnCl_4_,^51^ UiO-66-SO_3_H.^52^

In this work, MoO_3_/BF_3_ was prepared and identified as a highly efficient and recyclable acid nano-catalyst using FT-IR, XRD, FESEM, TEM, EDX, EDS-MAP, BET, and TGA analyses. Next, the catalyst was investigated for the synthesis of spirooxindoles and dihydro-2-oxopyrroles using a mixer mill, a green and economical method.

## Results and discussion

**Fig. 1:**
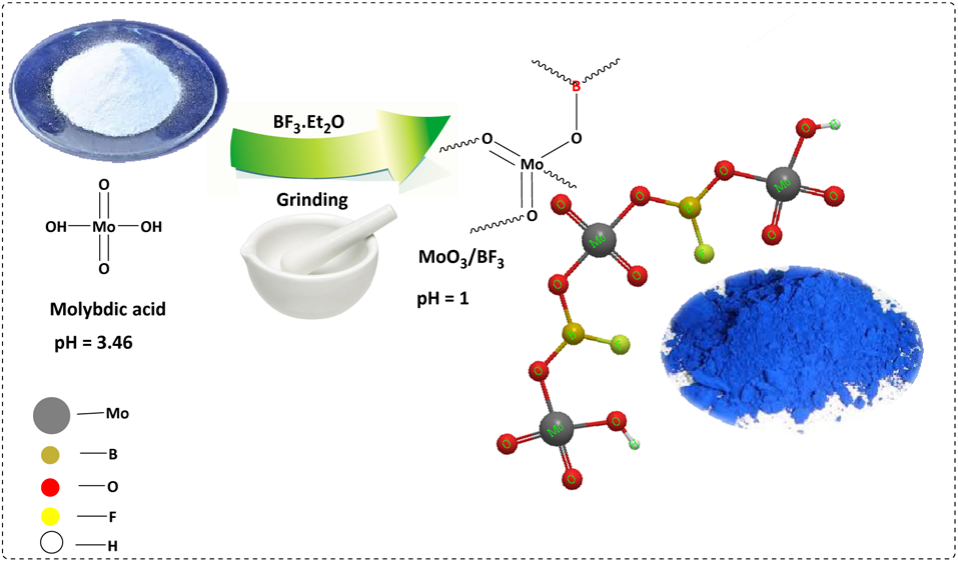
Preparation of MoO_3_/BF_3_.

The MoO_3_/BF_3_ catalyst was prepared (Fig. 1) and characterized using various analytical techniques, including FT-IR, XRD, FESEM, TEM, EDX, EDS-MAP, BET, and TGA.

### FT-IR of MoO_3_/BF_3_ nano-catalyst

**Fig. 2:**
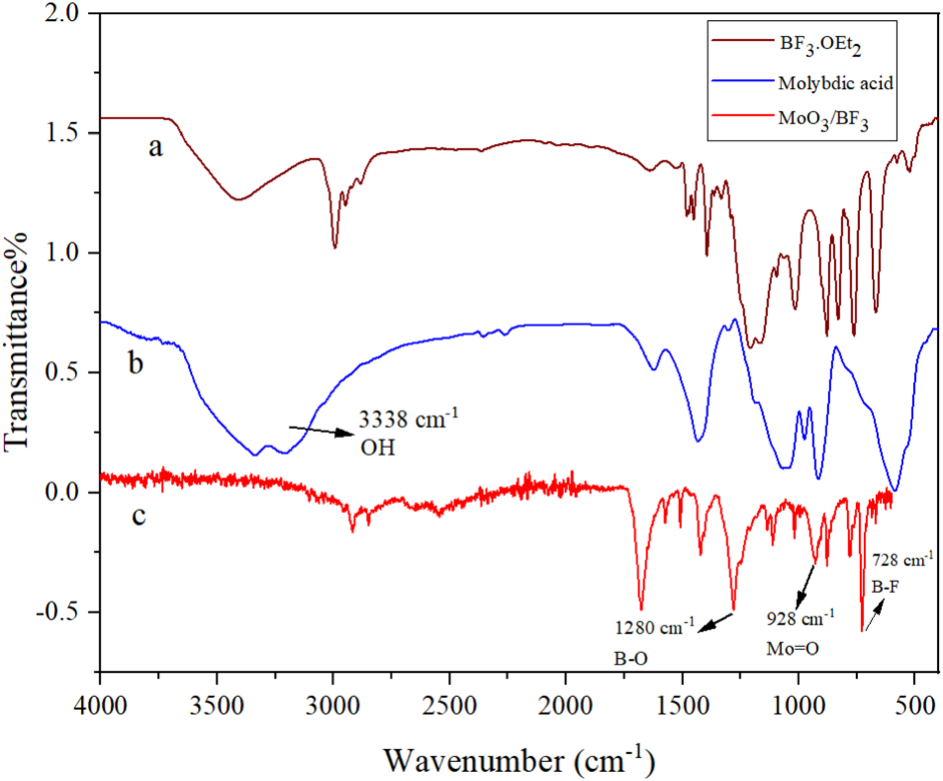
FT-IR Spectra of (a) BF_3_·OEt_2_, (b) molybdic acid, and (c) MoO_3_/BF_3_.

The FT-IR spectra of molybdic acid (a) and MoO_3_/BF_3_ nano-catalyst (b) were compared (Fig. 2). The broad absorption band observed in the spectrum (a) at 3338 cm^−1^ is related to the OH group of molybdic acid. In both spectra, the absorption band at 928 cm^−1^ corresponds to the stretching vibration of the Mo=O group. In the spectrum (b), the absorption band appearing in the 728 cm^−1^ and 1280 cm^−1^ corresponds to the stretching vibrations of the B–F and B–O groups, respectively. According to all these data, the formation of MoO_3_/BF_3_ nano-catalyst was confirmed.

### PXRD (powder X-ray diffraction) of MoO_3_/BF_3_

**Fig. 3:**
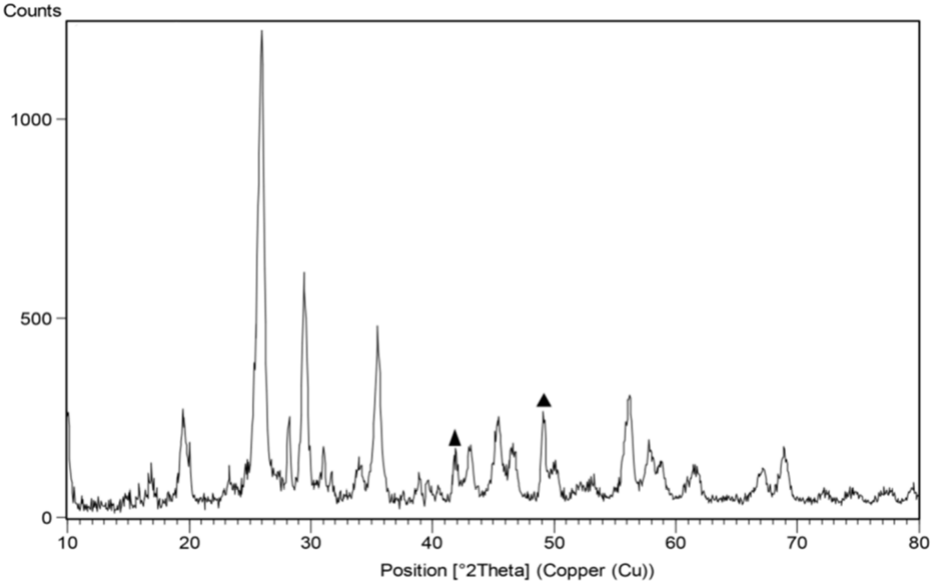
PXRD pattern of MoO_3_/BF_3_ nano-catalyst (the nano-catalyst shows some impurity peaks labeled in the black triangle (▲)).

The crystallography of the final MoO_3_/BF_3_ nano-catalyst was evaluated using the X-ray diffraction (XRD) pattern, shown in Fig. 3. All XRD peaks were consistent with the hexagonal phase of MoO_3_. A few impurity peaks were observed (▲), probably due to residual by-products formed by Mo^5+^ ions. The main peaks are at 2*θ* = 19.2 b 25.5° and, 29.9° which are characteristic of the hexagonal MoO_3_ phase in the final catalyst.^53^

### FESEM and TEM of MoO_3_/BF_3_ nano-catalyst

**Fig. 4:**
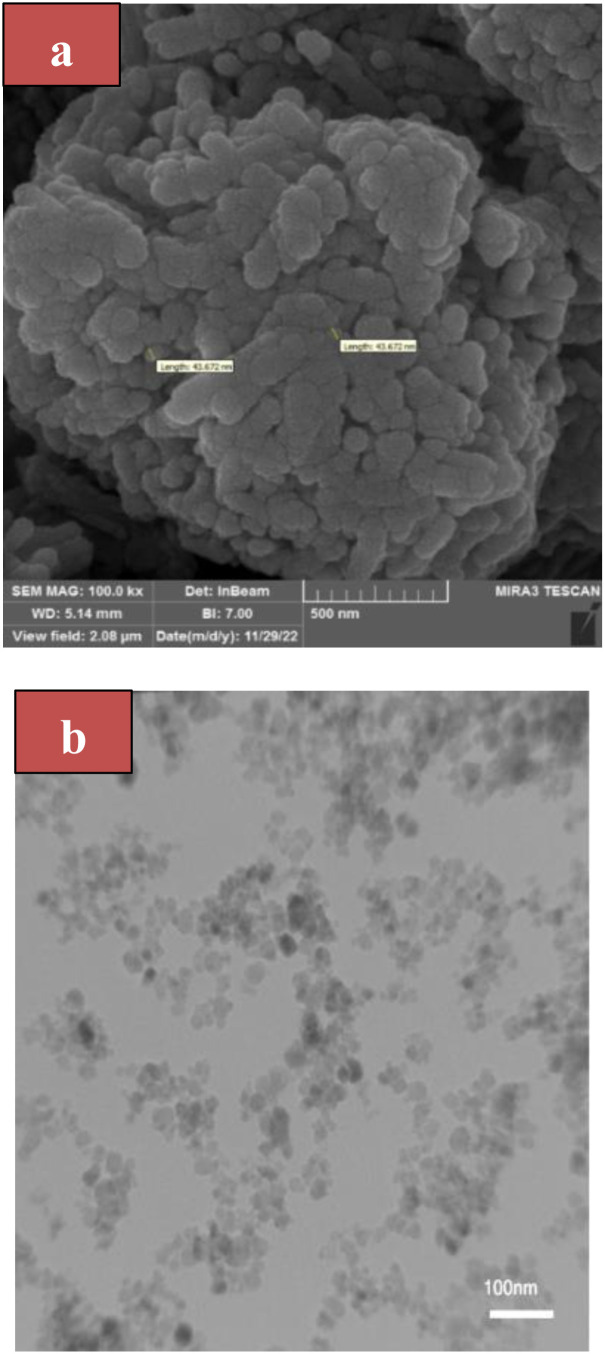
(a) FESEM image of MoO_3_/BF_3_ (b) TEM analysis of the prepared MoO_3_/BF_3_ catalyst at low magnification (100 nm).

In Fig. 4a, the field emission scanning electron microscope image of the final MoO_3_/BF_3_ nano-catalyst shows a quasi-spherical morphology with a diameter of 43 nm. TEM analysis of the prepared MoO_3_/BF_3_ catalyst depicts that the catalyst is a nanoparticle (Fig. 4b).

### EDX and EDS-map of MoO_3_/BF_3_

**Fig. 5:**
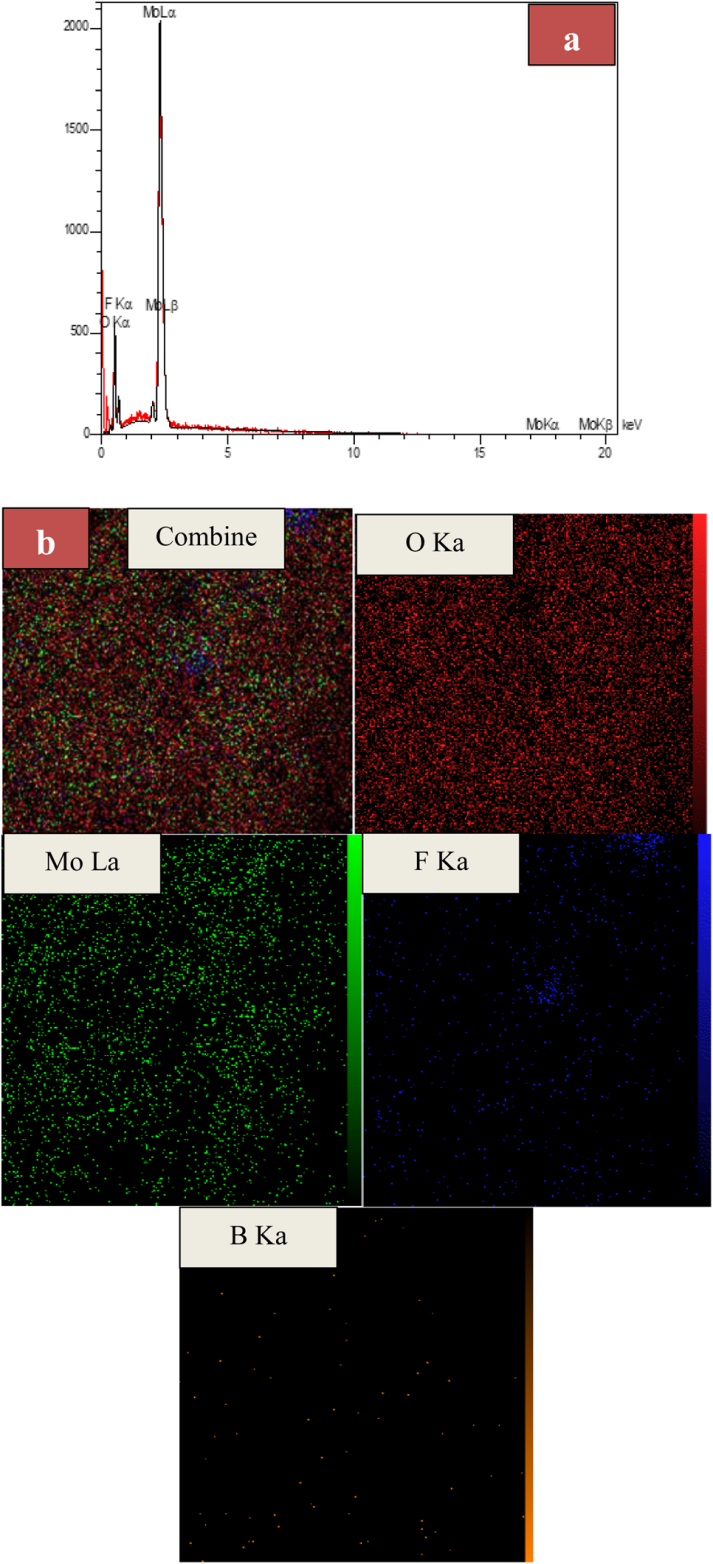
(a) EDX spectrum and (b) EDS-MAP of MoO_3_/BF_3_.

Fig. 5a shows the EDX spectrum of the final MoO_3_/BF_3_ nano-catalyst. As it is clear, all the elements in the final catalyst, including oxygen, molybdenum, and fluorine, have clear peaks. It confirms the presence of oxygen, molybdenum, fluorine, and boron elements in the prepared catalyst with 61.40%, 31.00%, 7.19%, and 0.41%, respectively. EDS-MAP of the final nano-catalyst shows the uniform and homogeneous distribution of elements on the surface of the catalyst (Fig. 5b).

### BET of MoO_3_/BF_3_

**Fig. 6:**
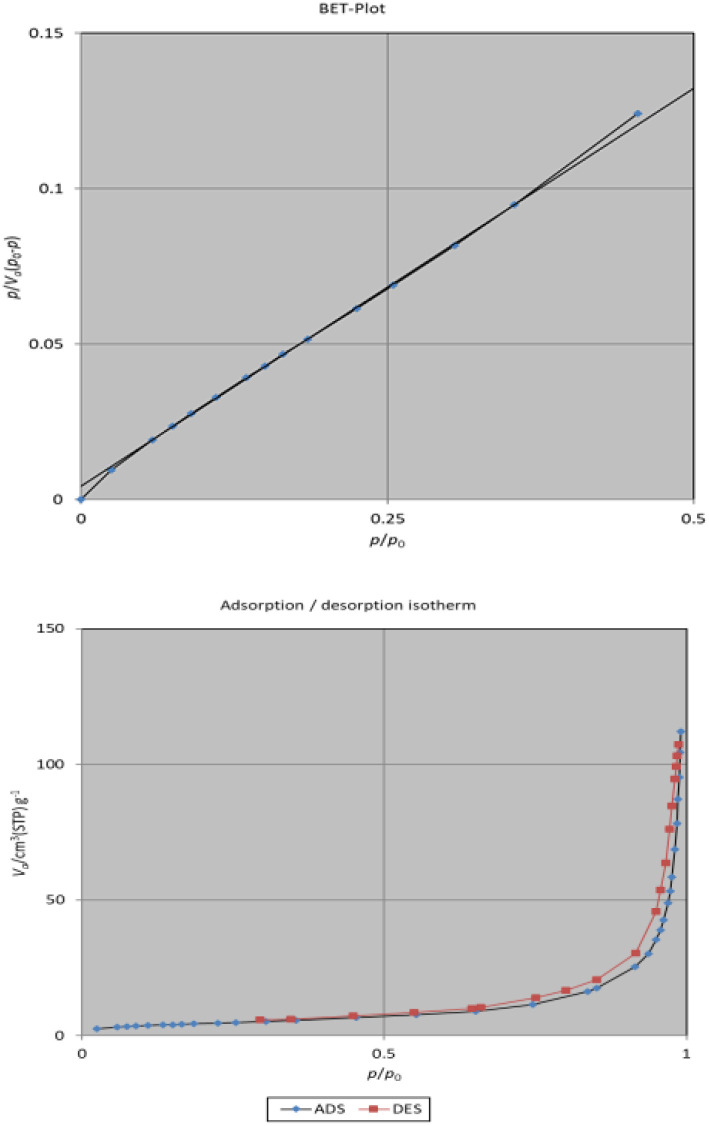
N_2_ adsorption (blue line)–desorption (red line) isotherm and corresponding diagrams pore size distributions.

The BET method was used to evaluate the amount and type of porosity as well as the surface area of the final nano-catalyst. Using the BET chart, the surface area of the MoO_3_/BF_3_ nano-catalyst was measured as 16.713 m^2^ g^−1^ (Fig. 6). The adsorption–desorption isotherm of N_2_ gas shows a type IV isotherm (Fig. 6) according to the IUPAC classification, which is characteristic of mesoporous and non-porous materials. In Table 1, information related to BJH, pore diameter, and total pore volume, are reported as 0.1689 cm^3^ g^−1^, 40.43 nm, and 0.1689 cm^3^ g^−1^, respectively.

**Table 1:** Summary of porosity parameters of MoO_3_/BF_3_ nano-catalyst

BET	
Vm	3.84 [cm^3^(STP) g^−1^]
as, BET	16.713 [m^2^ g^−1^]
C	60.97
Total pore volume (*p*/*p*_0_ = 0.990)	0.1689 [cm^3^ g^−1^]
Mean pore diameter	40.43 [nm]

**Langmuir plot**	
Vm	5.5722 [cm^3^(STP) g^−1^]
as,Lang	24.253 [m^2^ g^−1^]
B	0.2362

**t plot**	
Plot data	Adsorption branch
*a*_1_	14.509 [m^2^ g^−1^]
*V*_1_	0 [cm^3^ g^−1^]

**BJH plot**	
Plot data	Adsorption branch
Vp	0.1698 [cm^3^ g^−1^]
rp,peak (area)	4.61 [nm]
*a*_p_	21.382 [m^2^ g^−1^]

### TGA of MoO_3_/BF_3_

**Fig. 7:**
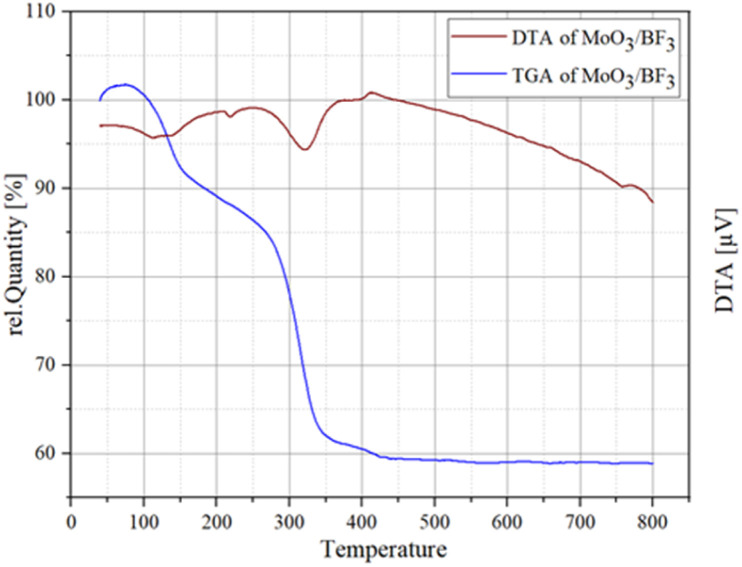
TGA (blue line) and DTA (red line) curves of MoO_3_/BF_3_.

Fig. 7 shows the curve obtained from the thermal weighting of the prepared nano-catalyst. According to this curve, mass reduction is observed at 100–150 °C, which can be attributed to the evaporation of absorbed water and solvents on the surface. The mass reduction occurred in the range of 200–350 °C, which is mainly related to the loss of existing hydroxyl groups. The third mass loss was recorded at 350–400 °C, the results depict that the presence of molecules of water and hydroxyl groups facilitates the stabilization of the hexagonal phase up to 400 °C, while above this temperature, the removal of these components leads to the transformation of the molybdic acid phase, as can be seen from the results, the present catalyst is suitable for the reaction up to 200 °C.^53^

### The catalytic activity of MoO_3_/BF_3_ nano-catalyst

The efficiency of the MoO_3_/BF_3_ nano-catalyst was investigated for the synthesis of nitrogen-containing heterocyclic compounds including, spirooxindoles and dihydro-2-oxopyrrole, by the Michael-Mannich cyclocondensation method and using the mill mixer, which is a safe method for the environment.

To achieve optimal conditions for the synthesis of spirooxindoles, at the outset, in a 10 mL stainless steel vial, with two stainless steel balls with a diameter of 0.8 mm, the reaction between isatin (1 mmol) malononitrile (1 mmol) and dimedone (1 mmol) were selected as model reactions. Then, different amounts of nano-catalyst (0.005 g to 0.02 g) and different solvents and temperatures were investigated to optimize the reaction conditions. The model reaction was carried out in various protic, and aprotic solvents and the absence of solvent. The reaction in the absence of solvent has a better yield than in the presence of solvent. To improve the performance and efficiency and reduce the reaction time, the effect of different frequencies of the mixer mill (10, 15, and 20 Hz) on the response of the model was investigated. The best frequency of the device was estimated to be 20 Hz. When the reaction was performed at lower frequencies, such as 10 Hz, some of the primary material was still present, possibly due to the reduced amount of energy per pulse. The results of this investigation are reported in Table 2. As it is evident, the best result, the highest efficiency, and the shortest time are related to the 0.01 g of the nano-catalyst in the mixer mill with a frequency of 20 Hz at room temperature and solvent-free conditions.

**Table 2:** Optimization of the reaction conditions for the synthesis of spirooxindole^a^

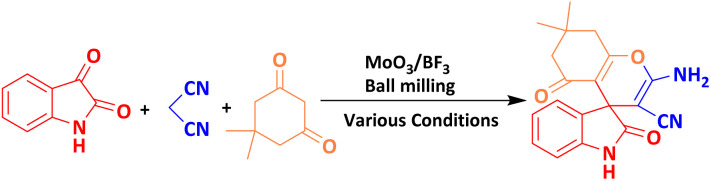			
Entry	Conditions	Time (min)	Yield^c^ (%)
	Solvent/temp. (°C)/catalyst (g)		
1	—/—/—	120	Trace
2	—/—/MoO_3_ (0.01)	60	43
3	—/—/BF_3_ (0.01)	60	30
4	—/—/MoO_3_/BF_3_ (0.005)	30	64
5	—/—/MoO_3_/BF_3_ (0.01)	10	98
6	—/—/MoO_3_/BF_3_ (0.015)	10	97
7	—/—/MoO_3_/BF_3_ (0.02)	10	97
8^b^	—/50/MoO_3_/BF_3_ (0.01)	30	89
9^b^	—/70/MoO_3_/BF_3_ (0.01)	35	73
10^b^	—/80/MoO_3_/BF_3_ (0.01)	60	73
11^b^	H_2_O/—/MoO_3_/BF_3_ (0.01)	60	67
12^b^	EtOH/—/MoO_3_/BF_3_ (0.01)	60	75
13^b^	H_2_O:EtOH/—/MoO_3_/BF_3_ (0.01)	60	70
14^b^	MeOH/—/MoO_3_/BF_3_ (0.01)	60	67
15^b^	CH_3_CN/—/MoO_3_/BF_3_ (0.01)	60	56

a Reaction conditions: isatin (1 mmol), 1,3-diketone (1 mmol), malononitrile, or ethyl cyanoacetate (1 mmol) were ground in a 10 mL stainless steel mixer vial with two balls at a frequency of 20 Hz.

b Magnetic stirring conditions.

c Isolated yield.

In the last step, after determining the optimal reaction conditions (MoO_3_/BF_3_ nano-catalyst 0.01 g, ground in a 10 mL stainless steel mixer mill vial with two stainless steel balls at a frequency of 20 Hz, room temperature, solvent-free), To investigate the generality of MoO_3_/BF_3_ nano-catalyst in the synthesis of spirooxindoles, different 1,3-diketone compounds were studied (Table 3). The active site of the MoO_3_/BF_3_ nano-catalyst is boron (B). According to the EDX analysis data, the reported weight percent B content in the prepared nano-catalyst is 0.41%. Here, we used 0.01 g of the catalyst for 1 mmol of substrate for the synthesis of spirooxindole. In this case, 0.01 g of catalyst is 4.1 × 10^−5^ g of B, equal to 0.0038 mmol of B. The measured TON and TOF for the model reaction (isatin, malononitrile, and dimedone) are 25 789.47 and 2578.94 min^−1^ respectively.

**Table 3:** MoO_3_/BF_3_-catalyzed synthesis of spirooxindole scaffolds^a^

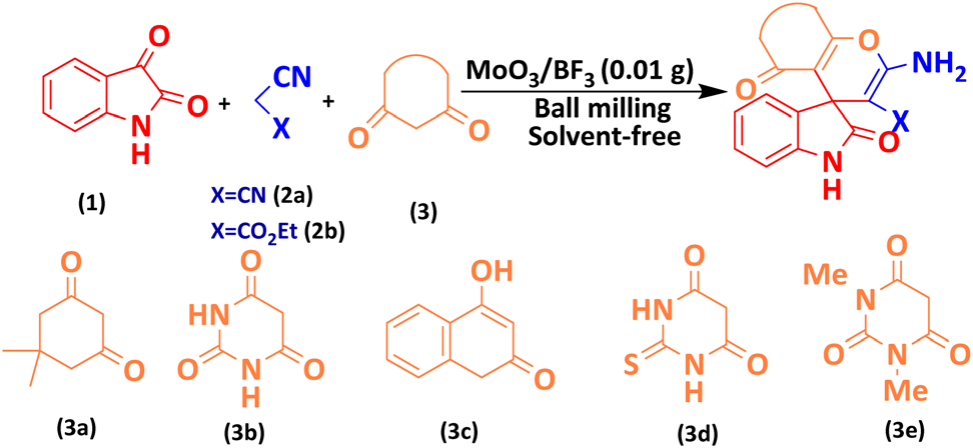							
Entry	1,3-Diketone	X	Product	Time (min)	Yield^b^ (%)	TON (TOF (min^−1^))	m.p. (°C) (ref.)
1	3a	2a	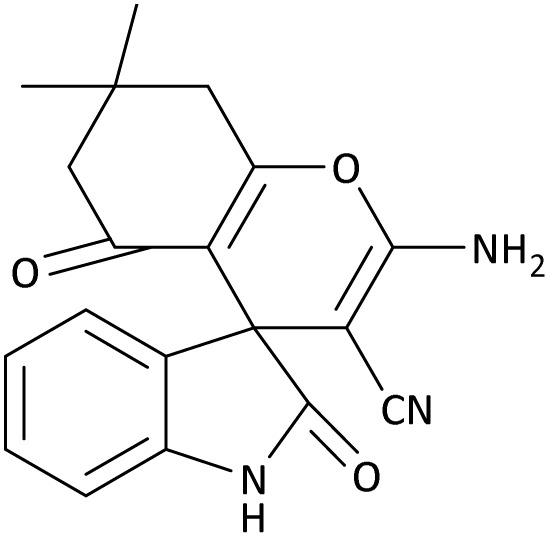	10	98	25 789.47 (2578.94)	290–292 (ref. 54)
2	3a	2b	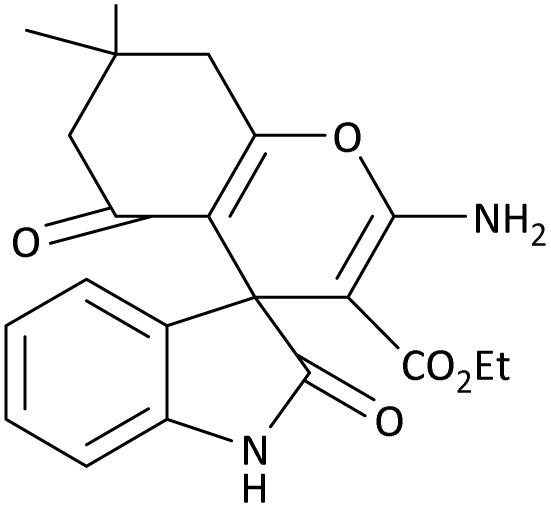	20	92	24 210.52 (1210.52)	230–232 (ref. 54)
3	3b	2a	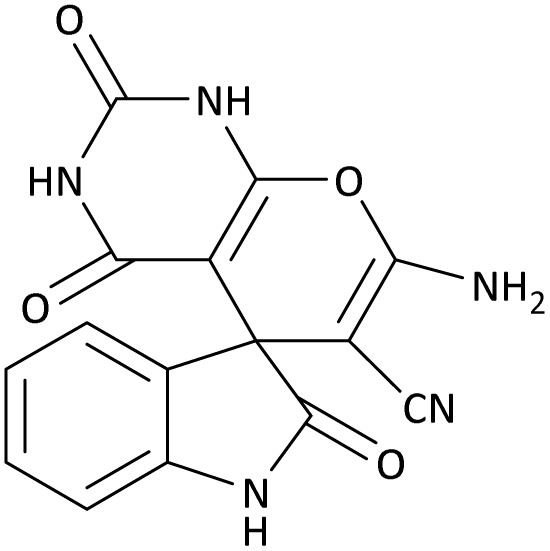	10	93	24 473.68 (2447.36)	270–272 (ref. 54)
4	3c	2a	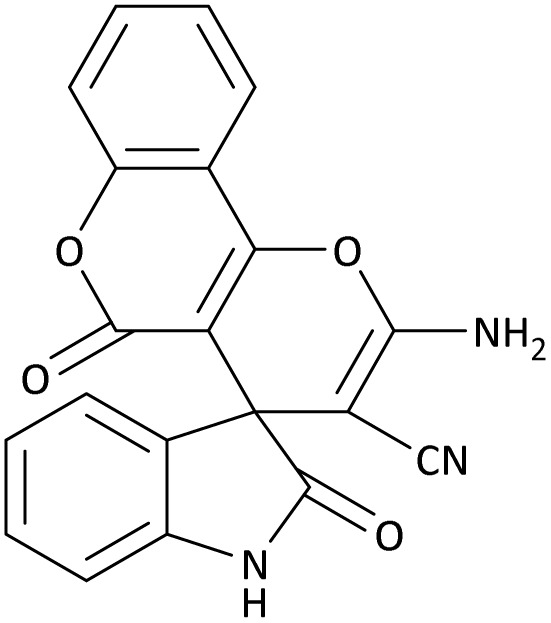	12	92	24 210.52 (2017.54)	293–295 [54]
5	3c	2b	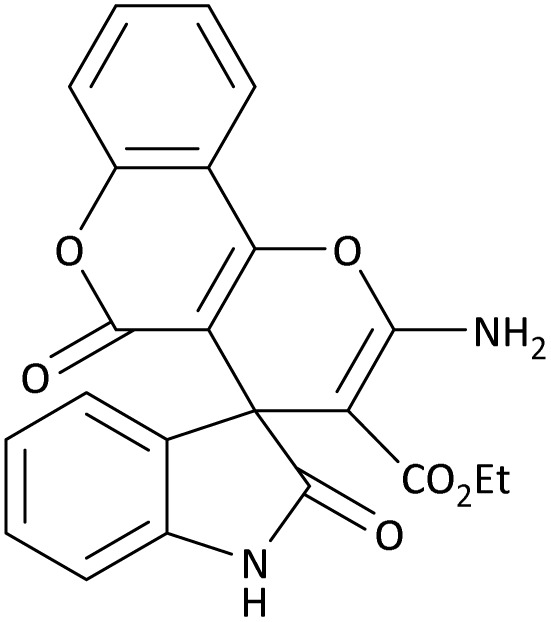	20	89	23 421.05 (1171.05)	210–212 (ref. 54)
6	3d	2a	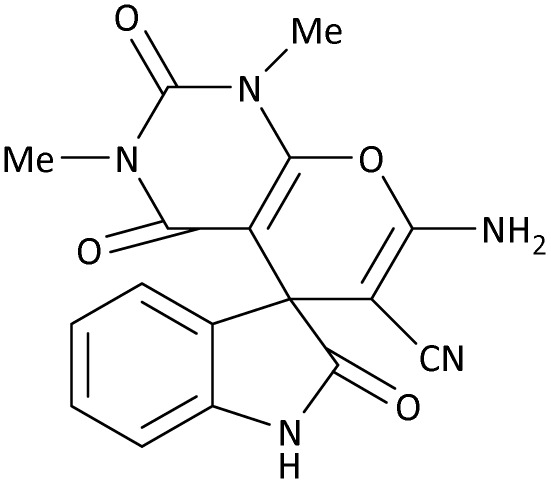	10	95	25 000 (2500)	244–246 (ref. 55)
7	3e	2a	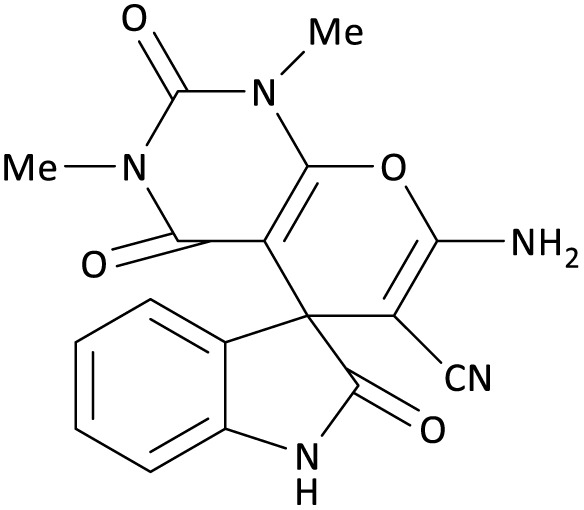	20	85	22 368.42 (1118.42)	221–224 (ref. 56)

a Reaction conditions: isatin (1 mmol), 1,3-diketone (1 mmol), and malononitrile or ethyl cyanoacetate (1 mmol) were ground in a 10 mL stainless steel vial of mixer mill with two balls at a frequency of 20 Hz, room temperature, 0.01 g catalyst.

b Isolated yield.

To investigate the catalytic activity of MoO_3_/BF_3_, in the next study, the present nano-catalyst was investigated for the synthesis of dihydro-2-oxopyrrole by the Michael-Mannich cyclocondensation method. To achieve the optimum conditions in the synthesis of dihydro-2-oxypyrrole, the reaction between dimethyl acetylene dicarboxylate (DMAD) (1 mmol), 4-chloroaniline (2 mmol), and formaldehyde (1.5 mmol) in the presence of MoO_3_/BF_3_ nano-catalyst in a stainless steel vial of 10 mL, with two stainless steel balls with a diameter of 0.8 mm, in various conditions were examined (Table 4).

**Table 4:** Optimization of experimental conditions of MoO_3_/BF_3_ for the synthesis of dihydro-2-oxopyrrole^a^

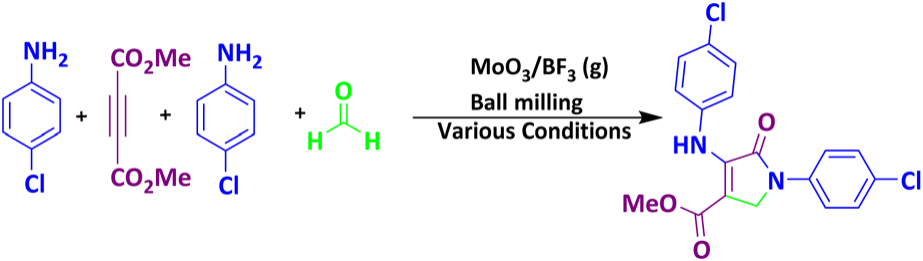			
Entry	Conditions	Time (min)	Yield^c^ (%)
	Solvent/temp. (°C)/catalyst (g)		
1	—/—/—	120	Trace
2	—/—/MoO_3_ (0.04)	120	64
3	—/—/BF_3_ (0.04)	120	58
4	—/—/MoO_3_/BF_3_ (0.005)	120	Trace
5	—/—/MoO_3_/BF_3_ (0.01)	60	30
6	—/—/MoO_3_/BF_3_ (0.015)	45	38
7	—/—/MoO_3_/BF_3_ (0.02)	45	40
8	—/—/MoO_3_/BF_3_ (0.025)	45	59
9	—/—/MoO_3_/BF_3_ (0.03)	30	68
10	—/—/MoO_3_/BF_3_ (0.035)	30	89
11	—/—/MoO_3_/BF_3_ (0.04)	25	95
12	—/—/MoO_3_/BF_3_ (0.05)	25	95
13^b^	—/50/MoO_3_/BF_3_ (0.04)	64	89
14^b^	—/70/MoO_3_/BF_3_ (0.04)	85	73
15^b^	—/80/MoO_3_/BF_3_ (0.04)	110	72
16^b^	H_2_O/—/MoO_3_/BF_3_ (0.04)	60	72
17^b^	EtOH/—/MoO_3_/BF_3_ (0.04)	60	80
18^b^	H_2_O:EtOH/—/MoO_3_/BF_3_ (0.041)	60	85
19^b^	MeOH/—/MoO_3_/BF_3_ (0.04)	60	74

a Reaction conditions: DMAD (1 mmol), 4-chloroaniline (2 mmol), and formaldehyde (1.5 mmol) were ground in a 10 mL stainless steel mixer vial with two balls at a frequency of 20 Hz.

b Magnetic stirring conditions.

c Isolated yield.

The scope of this methodology was evaluated using dimethyl\ethyl acetylene dicarboxylate, formaldehyde, and various aromatic amines under optimal conditions and the desired oxopyrroles products were synthesized with good to excellent yields (Table 5). Here, 0.04 g of catalyst was used for 1 mmol of substrate for the synthesis of dihydro-2-oxopyrrole which, 0.04 g of catalyst contains 1.6 × 10^−4^ g of B, equal to 0.0151 mmol of B. The measured TON and TOF for the model reaction are 6291.39 and 251.65 min^−1^ respectively.

**Table 5:** Synthesis of dihydro-2-oxopyrrole derivatives in the presence of MoO_3_/BF_3_^a^

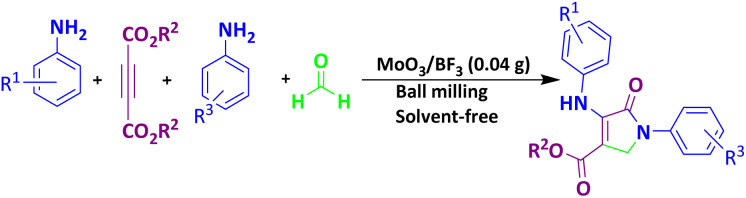								
Entry	R^1^	R^2^	R^3^	Product	Time (min)	Yield^b^ (%)	TON (TOF (min^−1^))	m. p. (°C) (ref.)
1	4-Cl^−^	Me	4-Cl^−^	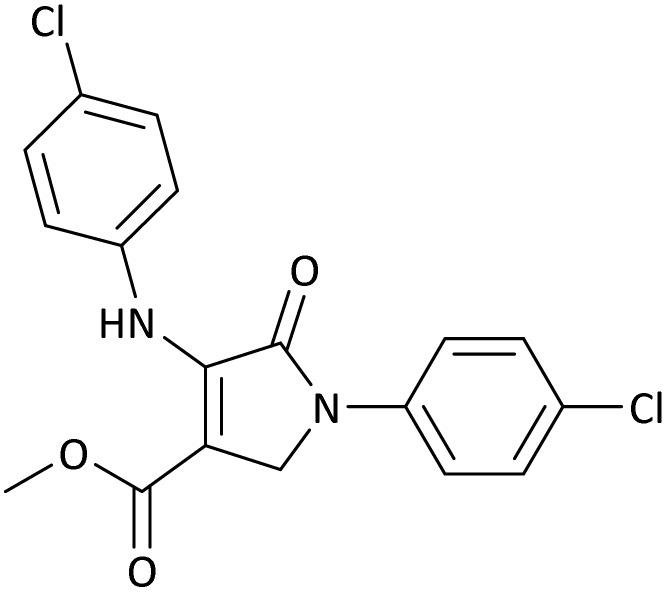	25	95	6291.39 (251.65)	173–174 (ref. 51)
2	4-Cl^−^	Et	4-Cl^−^	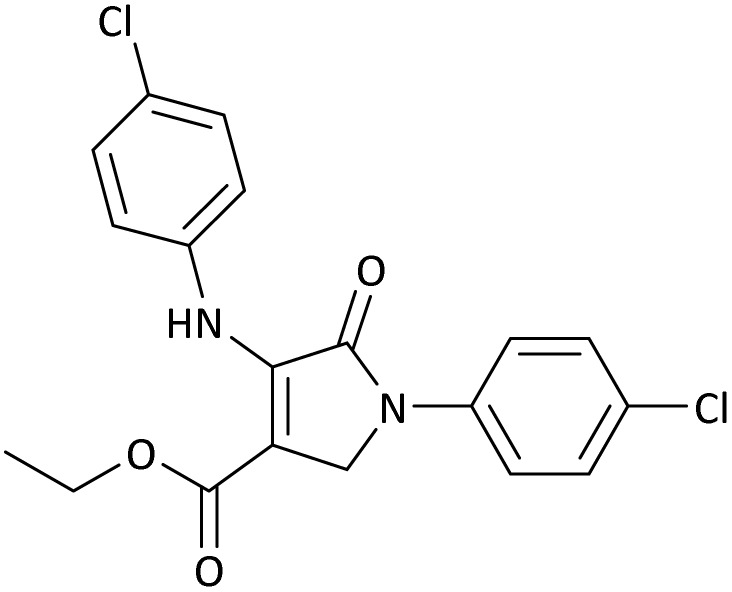	35	90	5060.26 (170.29)	165–167 (ref. 47)
3	4-Br^−^	Me	4-Br^−^	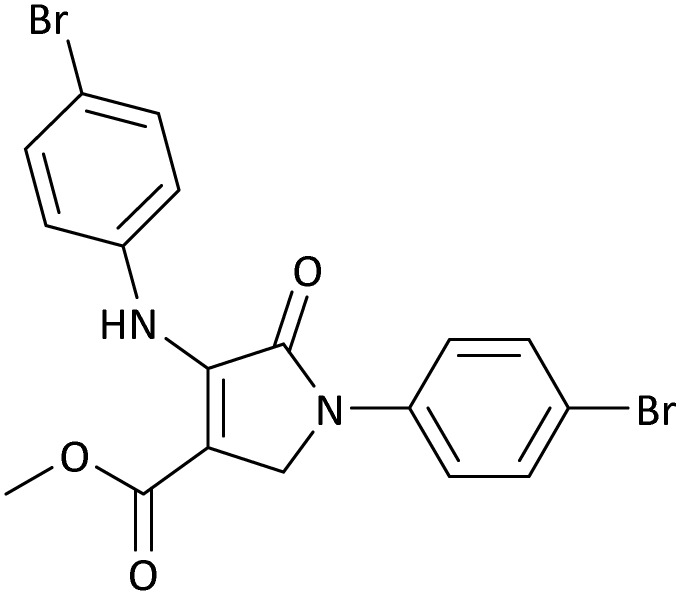	20	95	6291.39 (314.56)	181–182 (ref. 47)
4	4-Br^−^	Et	4-Br^−^	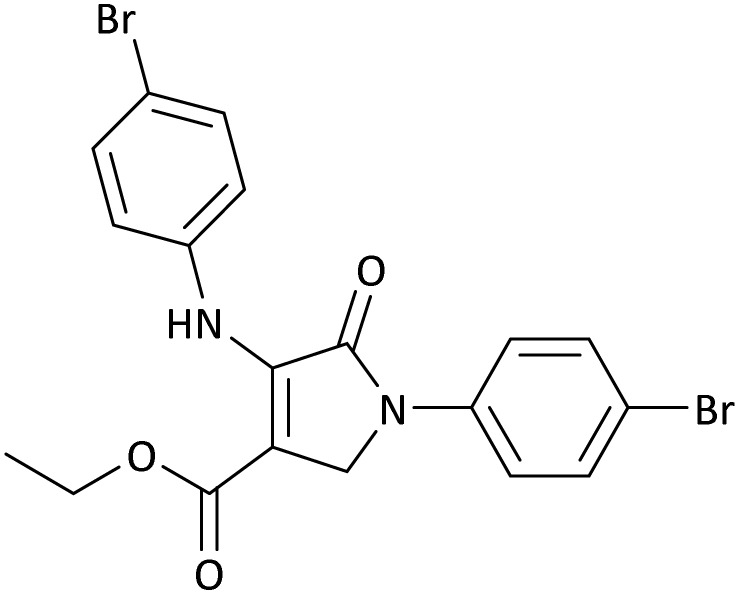	25	95	6291.39 (251.65)	165–166 (ref. 51)
5	4-NO_2_^−^	Et	4-NO_2_^−^	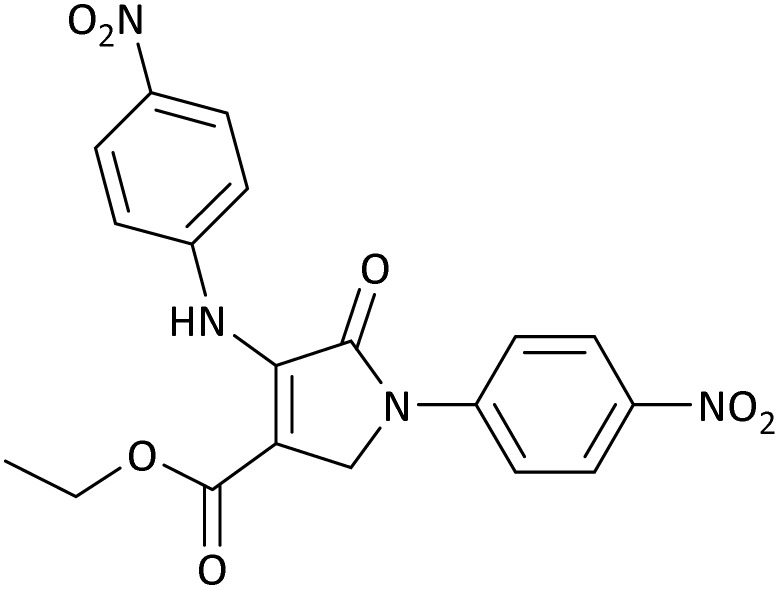	35	95	6291.39 (179.75)	206–208 (ref. 51)
6	3-NO_2_^−^	Me	3-NO_2_^−^	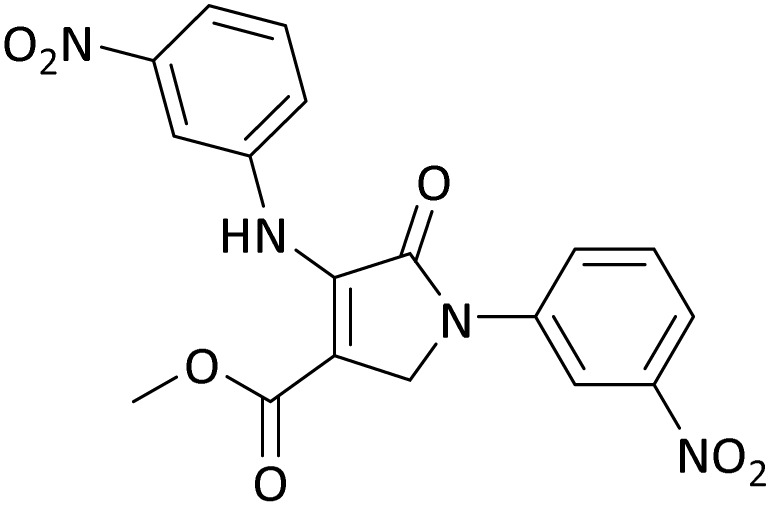	35	90	5960.26 (170.29)	202–204 (ref. 51)
7	3-NO_2_^−^	Et	3-NO_2_^−^	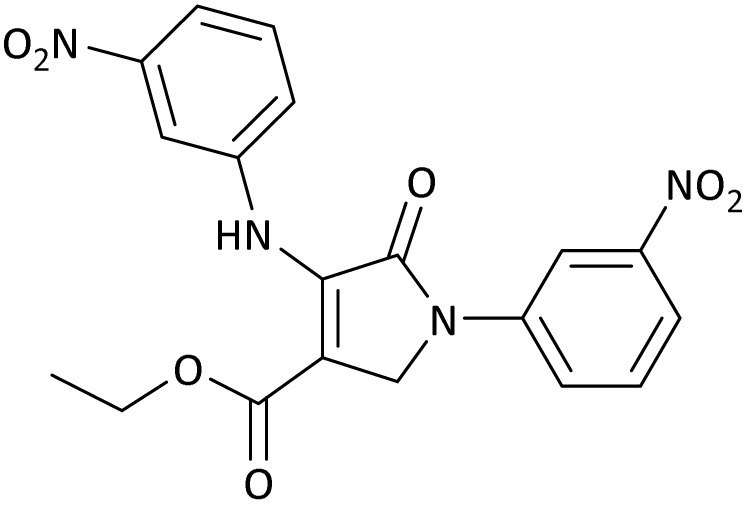	40	80	5298.01 (132.45)	190–192 (ref. 51)
8	4-Me^−^	Me	4-Me^−^	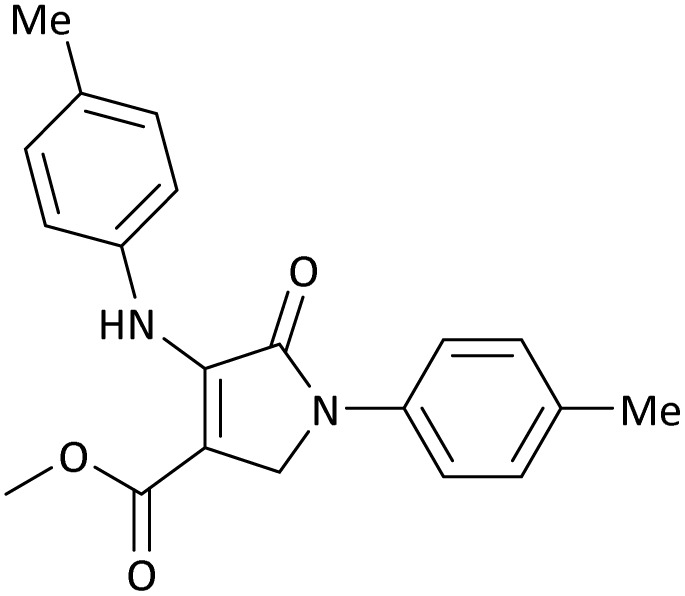	20	89	5894.04 (294.70)	174–176 (ref. 47)
9	4-Et^−^	Me	4-Et^−^	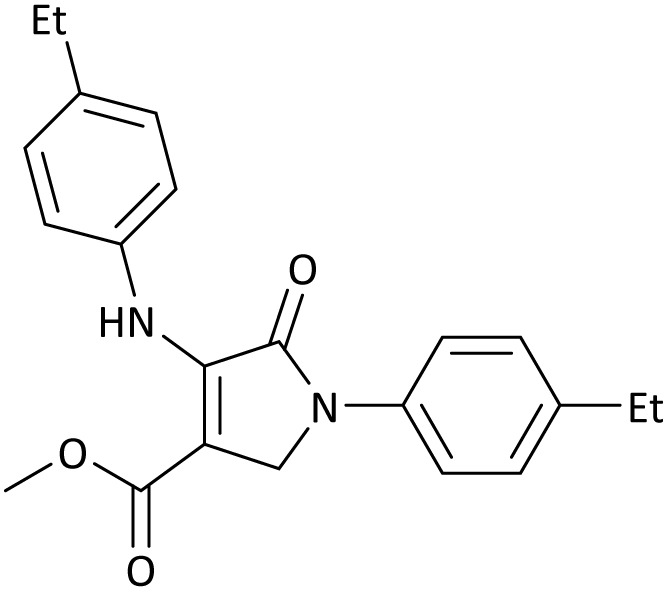	25	90	5960.26 (238.41)	125–126 (ref. 51)
10	4-OMe	Me	4-OMe	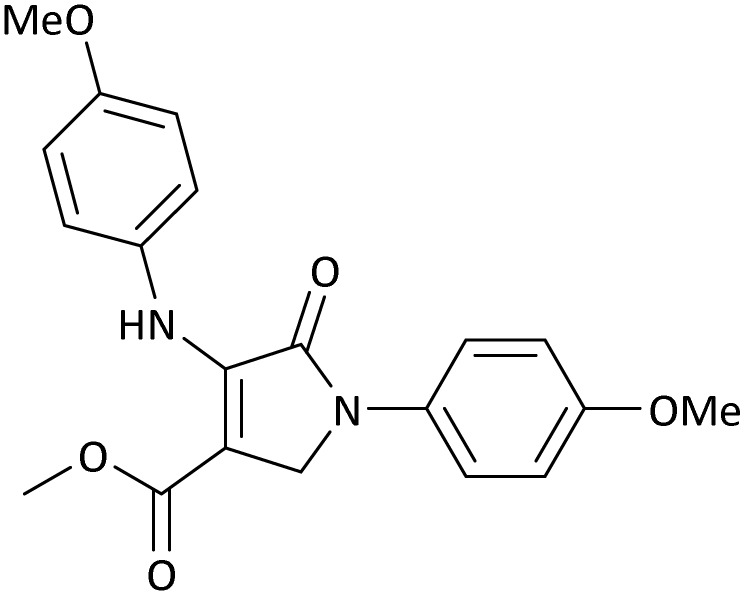	20	95	6291.39 (314.57)	160–162 (ref. 51)
11	4-OMe	Et	4-OMe	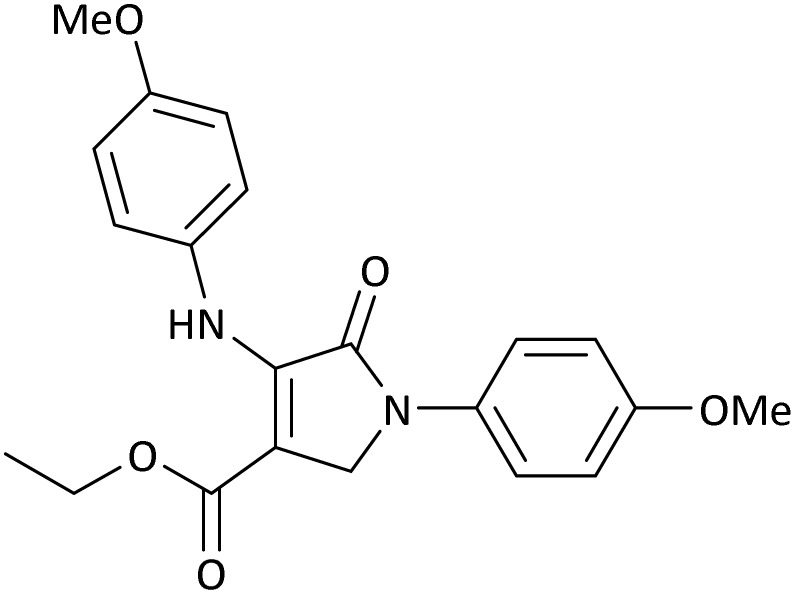	25	90	5960.26 (238.41)	152–154 (ref. 51)

a Reaction conditions: DAAD (1 mmol), aromatic amine (2 mmol), and formaldehyde (1.5 mmol) were ground in a 10 mL stainless steel vial of mixer mill with two balls at a frequency of 20 Hz, room temperature, 0.04 g catalyst.

b Isolated yield.

### A plausible mechanism for the synthesis of spirooxindole

**Scheme 2:**
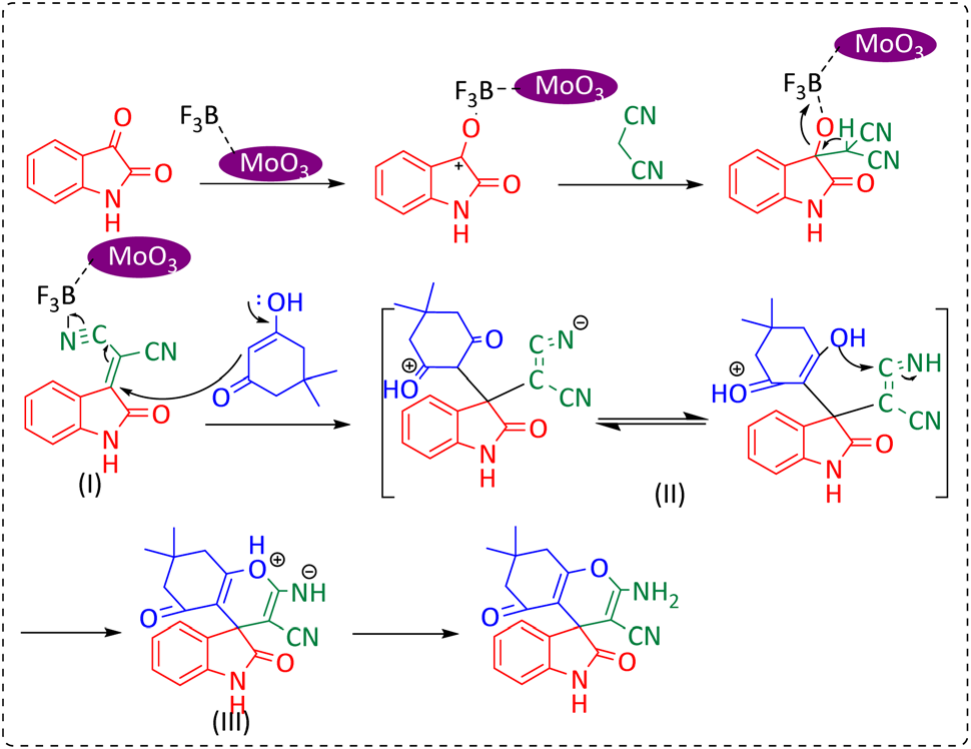
Proposed mechanism for the synthesis of spirooxindole.

The proposed mechanism for the synthesis of spirooxindoles derivatives in the presence of MoO_3_/BF_3_ nano-catalyst is shown in Scheme 2. At first, the carbonyl group of isatin was activated by BF_3_ nano-catalyst, then, by the attack of methylene carbon of malononitrile through Knoevenagel condensation to carbonyl isatin and removal of a water molecule, alkenyl is formed. Then, by increasing Michael between the 1,3-dicarbonyl compound and intermediate (II), compound (III) is formed, and finally, the desired product is obtained by cyclization and tautomerism.^9^

### A proposed mechanism for the formation of dihydro-2-oxopyrrole

**Scheme 3:**
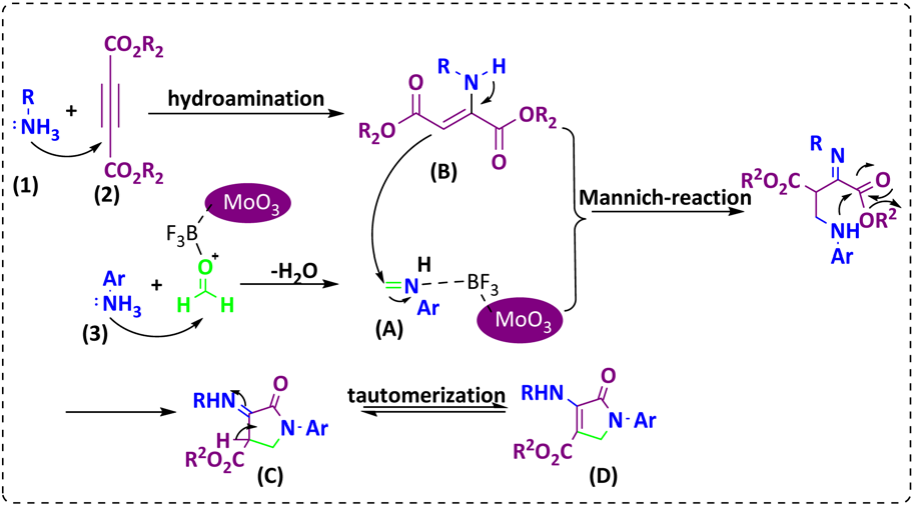
Suggested mechanism for the synthesis of dihydro-2-oxopyrrole.

The proposed mechanism for the synthesis of dihydro-2-oxypyrrole by the Michael-Mannich cyclocondensation method is shown in Scheme 3. In the presence of MoO_3_/BF_3_ nano-catalyst, a reaction occurs between amine (3) and formaldehyde to create imine (A). Amine (1) and DAAD (2) perform a Mannich-type reaction response to produce intermediate (A) and then enamine (B) performs tautomerization to form intermediate (C) and then the more stable form (D).^57^

To show the efficiency and merit of the nano-catalyst, the model reaction for the synthesis of spirooxindole (isatin, malononitrile, dimedone) and dihydro-2-oxopyrrole (DMAD, form aldehyde, 4-chloroaniline) in the presence of this nano-catalyst was compared with other reported nano-catalysts (Tables 6 and 7 respectively). Easy recyclability, high acid strength (pH = 1), short reaction time, and high efficiency without significant loss of nano-catalyst performance are among the advantages of this nano-catalyst.

**Table 6:** The comparison result of MoO_3_/BF_3_ with the reported catalysts in the literature for the synthesis of spirooxindole

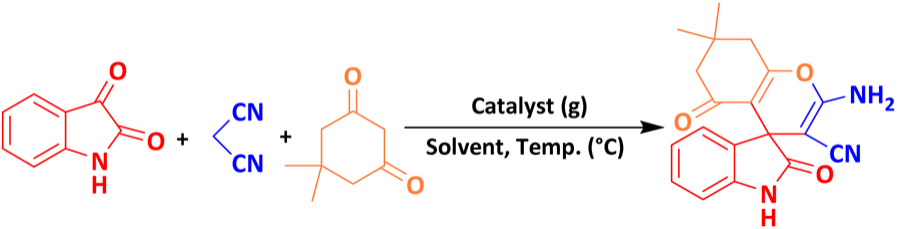				
Entry	Conditions	Time (min)	Yield (%)	References
	Solvent/temp. (°C)/catalyst			
1	H_2_O:EtOH/—/HPA@HNTs-IMI-SO_3_H (0.025 g)	15	95	58
2	H_2_O: EtOH/ref./GN/SO_3_H (0.08 g)	40	98	28
3	—/80/urea : ChCl (2 : 1) (0.5 mL)	60	95	20
4	H_2_O: EtOH/80/citric acid (0.038 g)	10	81	54
5	EtOH/—/[bmim]OH (1 mmol)	10	94	59
6	—/—/MoO_3_/BF_3_ (0.01 g)	10	98	This work

**Table 7:** Comparison of synthesis of dihydro-2-oxopyrrole with reported protocols

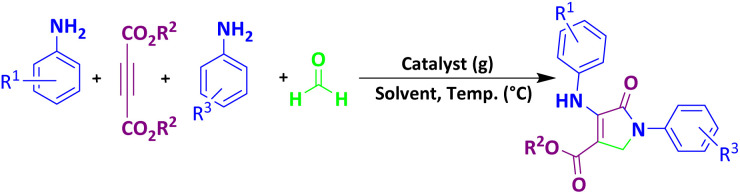				
Entry	Conditions	Time (min)	Yield (%)	References
	Solvent/temp. (°C)/catalyst			
1	EtOH/65/nano-Fe_3_O_4_@SiO_2_/SnCl_4_ (0.04 g)	60	97	51
2	MeOH/—/glycine amino acid (10 mol%)	180	93	46
3	MeOH/—/I_2_ (10 mol%)	60	82	48
4	—/—/MoO_3_/BF_3_(0.04 g)	25	95	This work

### Reusability of MoO_3_/BF_3_

**Fig. 8:**
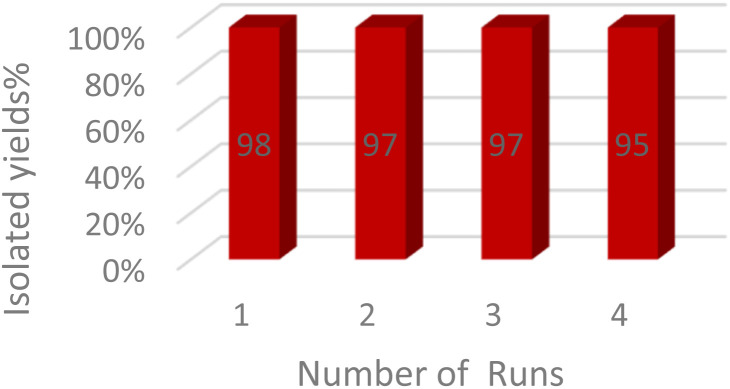
Reusability of MoO_3_/BF_3_ for the synthesis of spirooxindole.

To check the recyclability of the MoO_3_/BF_3_ nano-catalyst, the template reaction for the synthesis of spirooxindole (isatin, malonitrile, dimedone) was evaluated under optimal conditions (Fig. 8). The results showed that the nano-catalyst can be reused for up to four consecutive stages without a significant drop in efficiency. After the completion of the reaction, hot ethanol was added to the reaction mixture, and the MoO_3_/BF_3_ nano-catalyst was separated from the reaction mixture by simple filtration, washed several times with ethanol, then dried at ambient temperature, and used in the subsequent reactions.

**Fig. 9:**
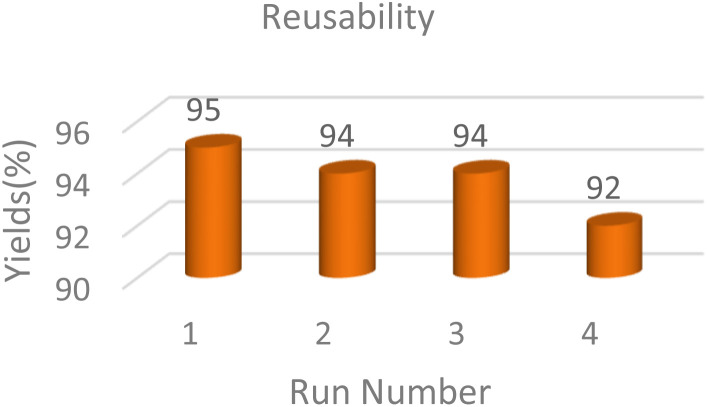
Reusability study for the synthesis of dihydro-2-oxopyrrole.

To determine the recyclability of the MoO_3_/BF_3_ nano-catalyst, the model reaction for the synthesis of dihydro-2-oxopyrrole, after the completion of the reaction, hot ethanol was added to the reaction mixture and scraped. The MoO_3_/BF_3_ nano-catalyst was separated from the reaction mixture by simple filtration, washed several times with hot ethanol, dried at ambient temperature, and used in the subsequent steps. The results showed that the nano-catalyst can be reused for up to three consecutive steps without significantly reducing efficiency (Fig. 9).

### Leaching test

**Fig. 10:**
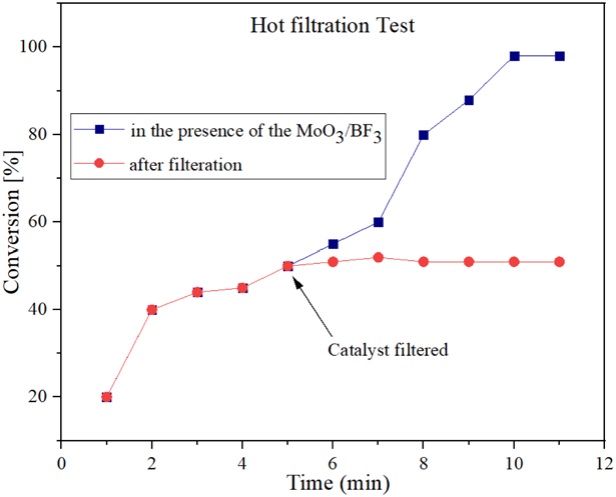
Hot filtration test and leaching effect to investigate the heterogeneous nature of MoO_3_/BF_3_.

To investigate the leakage of BF_3_ from the catalyst during the reaction, the hot filtration leaching test was performed for the synthesis of spirooxindole. The catalytically active particles were removed from the reaction by filtration after 5 min using hot filtration. After hot filtration, the reaction yield did not change and no reaction progress was obtained, which indicates that the nano-catalyst did not leak into the reaction mixture (Fig. 10).

### Methods of computation

In this work, the calculation of the spirooxindoles and dihydro-2-oxopyrrole derivatives to determine the most stable combinations was performed using the method of density functional theory (DFT) by the Becke-3-Lee–Yang–Parr (B3LYP) with a 6–311G (d,p) basis set in the Gaussian 09 W program.^60–66^ The total energy and electronic band gap energy (*E*_g_ = *E*_HOMO_ − *E*_LUMO_) computations for all dihydro-2-oxopyrrole derivatives and spirooxindoles are given in Tables 8 and 9, respectively. The results of these tables demonstrated that the stability of compound 3 in dihydro-2-oxopyrrole derivatives and compound 6 in spirooxindoles is greater than that of the other reported compounds in the 8 and 9 tables, according to stability statistics and total energies.

**Table 8:** The electronic band gap energy (*E*_g_ = *E*_HOMO_ − *E*_LUMO_) and total energy computations for all dihydro-2-oxopyrrole derivatives

Entry	R^1^	R^2^	R^3^	R	*E*_tot_ (a.u.)	*E*_g_ = *E*_HOMO_ − *E*_LUMO_
1	4-Cl^−^	Me	4-Cl^−^	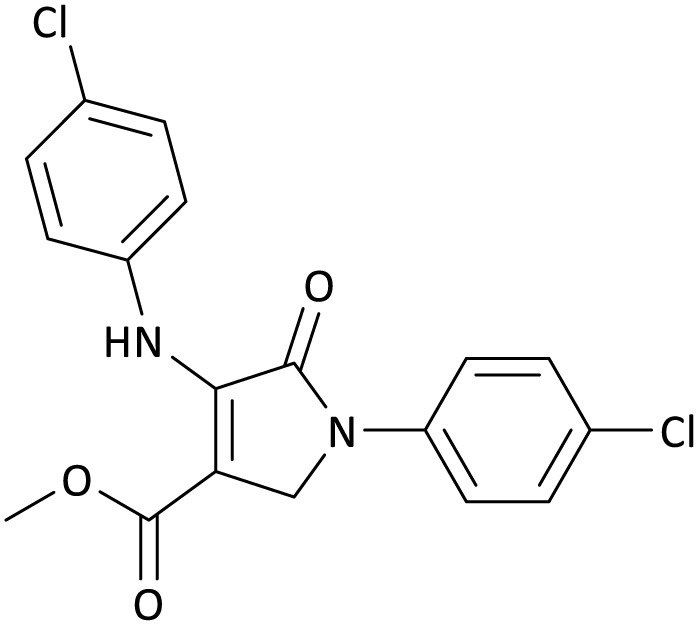	−5938.63329146	0.11524
2	4-Cl^−^	Et	4-Cl^−^	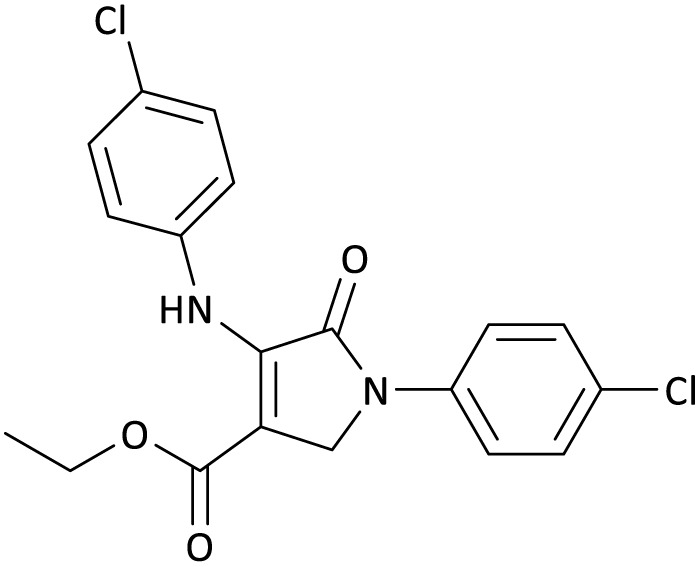	−1477.74163106	0.11311
3	4-Br^−^	Me	4-Br^−^	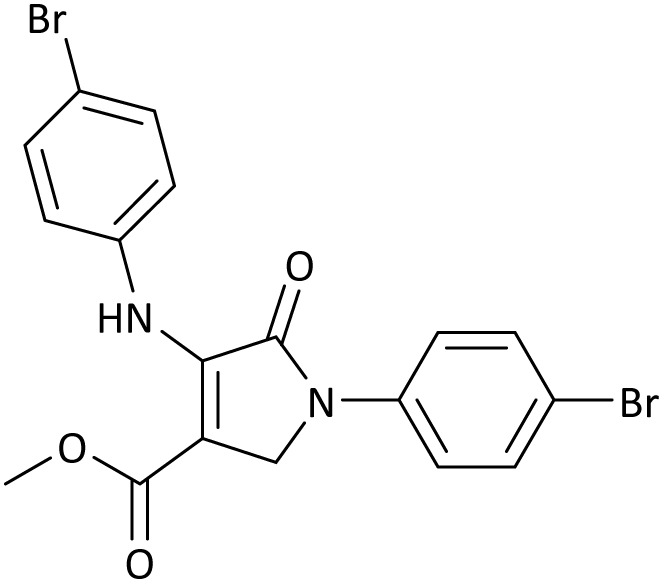	−6185.36730055	0.11763
4	4-Br^−^	Et	4-Br^−^	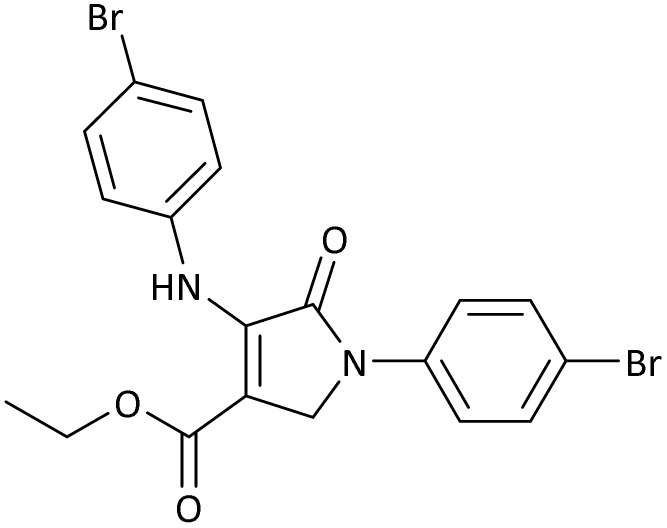	−6146.25897292	0.11748
5	4-NO_2_^−^	Et	4-NO_2_^−^	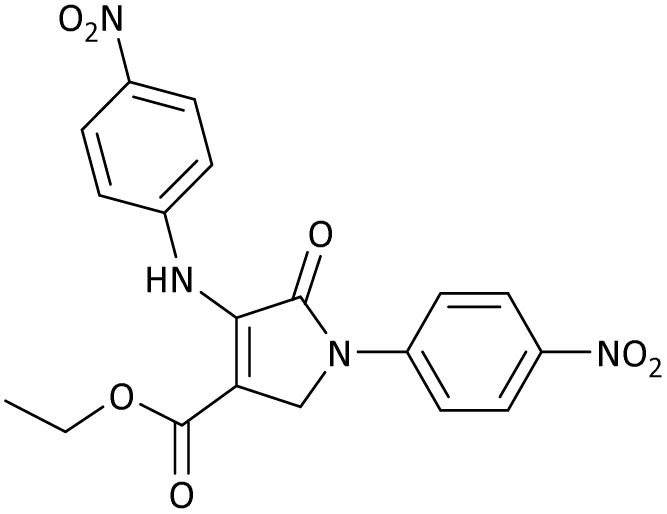	−5469.63456394	0.11415
6	3-NO_2_^−^	Me	3-NO_2_^−^	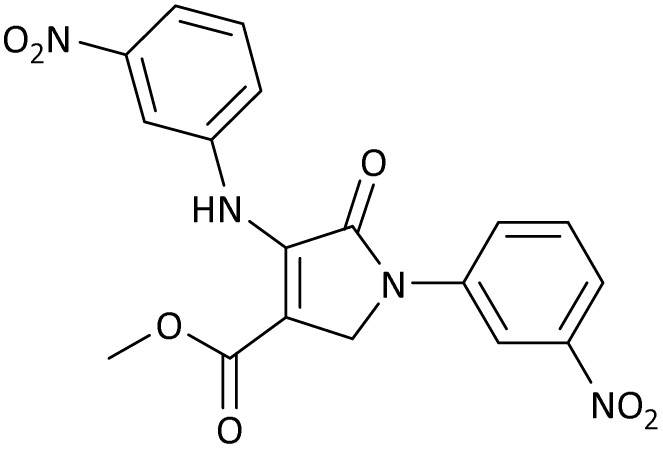	−1430.52537598	0.11209
7	3-NO_2_^−^	Et	3-NO_2_^−^	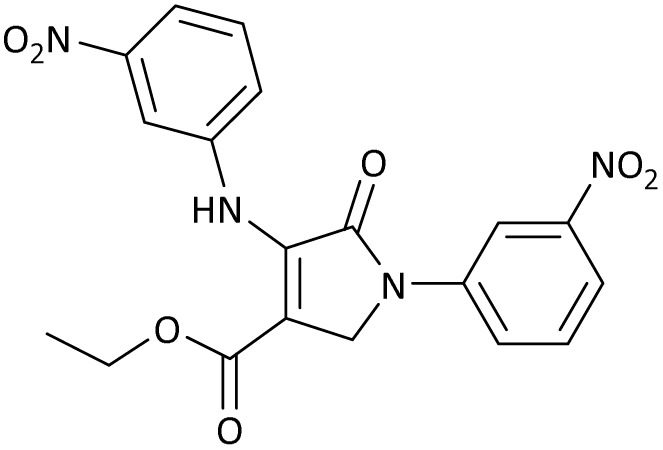	−1416.63308118	0.11098
8	4-Me^−^	Me	4-Me^−^	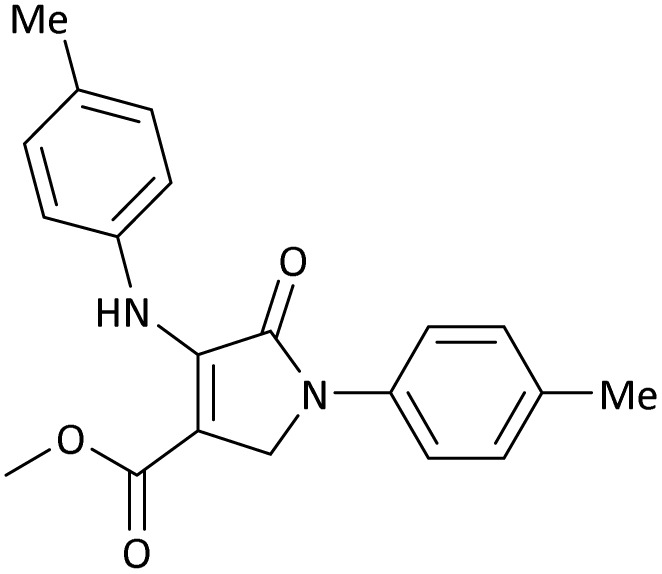	−1432.05492778	0.11073
9	4-Et^−^	Me	4-Et^−^	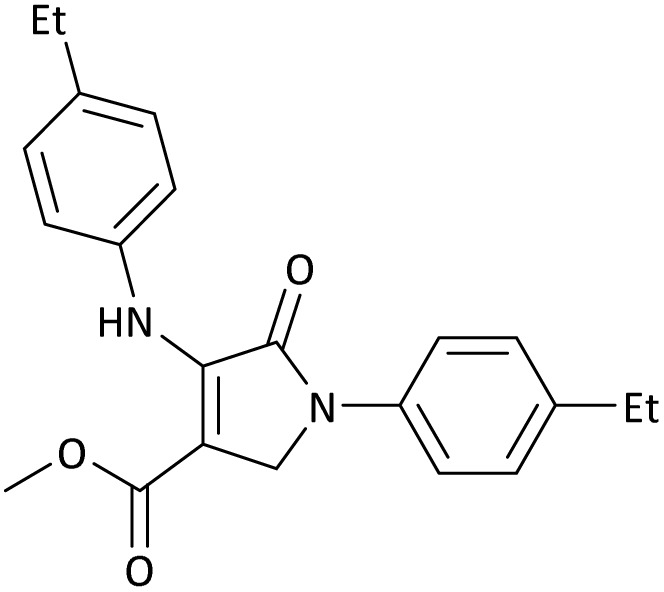	−1480.25623350	0.11203
10	4-OMe	Me	4-OMe	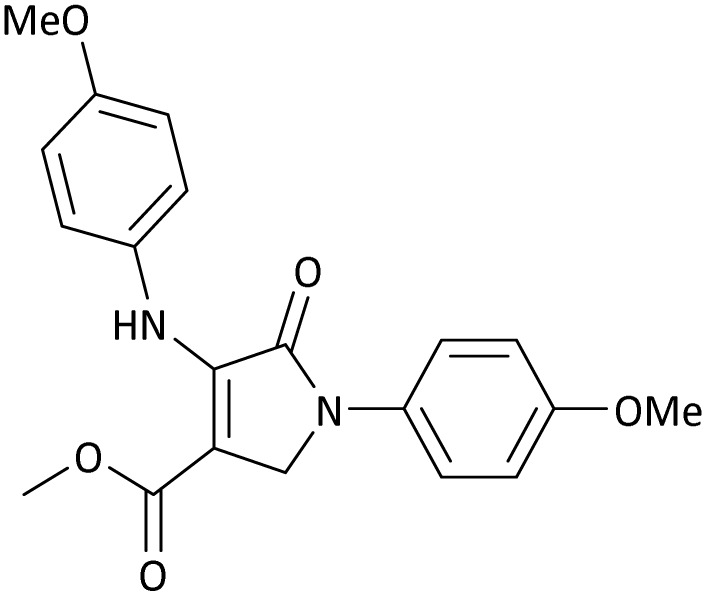	−6185.13898950	0.11751
11	4-OMe	Et	4-OMe	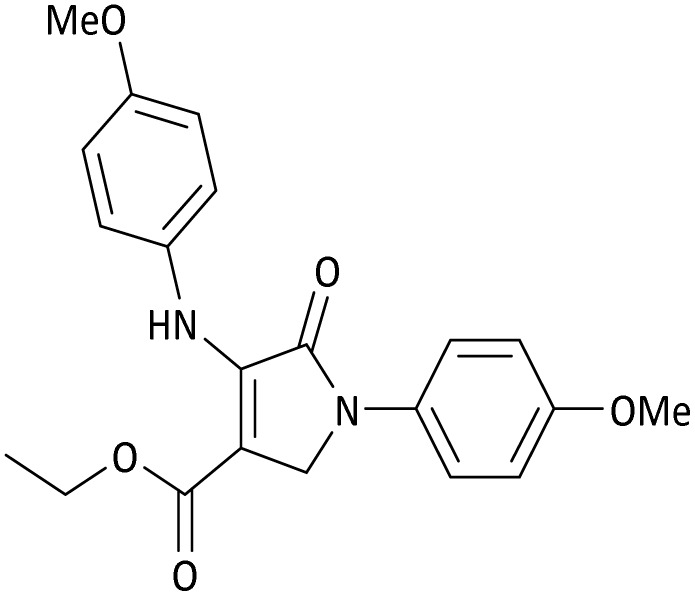	−1480.14731210	0.11101

**Table 9:** The total energy and electronic band gap energy (*E*_g_ = *E*_HOMO_ − *E*_LUMO_) calculations for all spirooxindoles

Entry	1,3-Diketone	X	*R*	*E*_tot_ (a.u)	*E*_g_ = *E*_HOMO_ − *E*_LUMO_
1	3a	2a	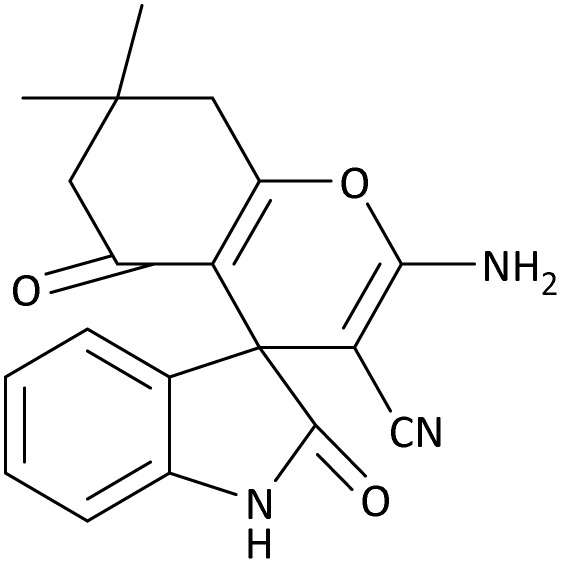	−1515.34722503	0.17006
2	3a	2b	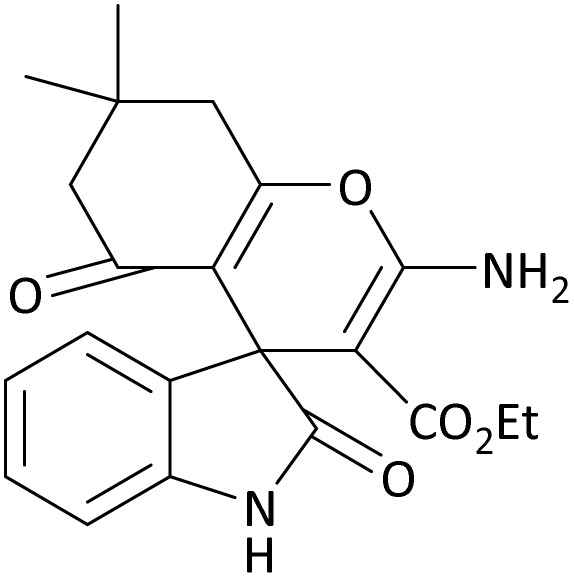	−1289.35423416	0.13277
3	3b	2a	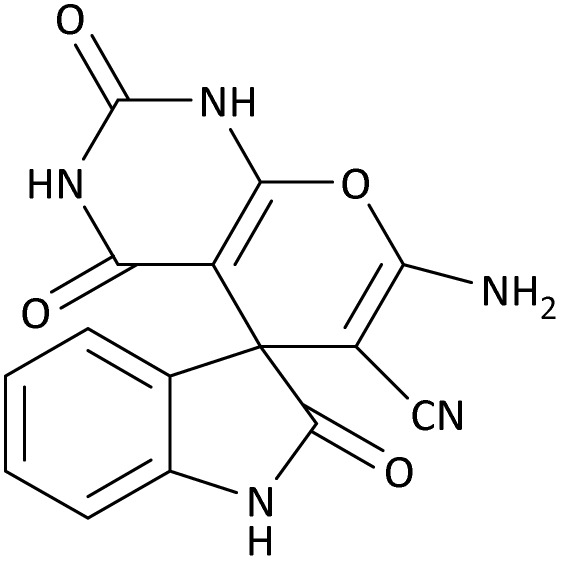	−1344.01838347	0.14494
4	3c	2a	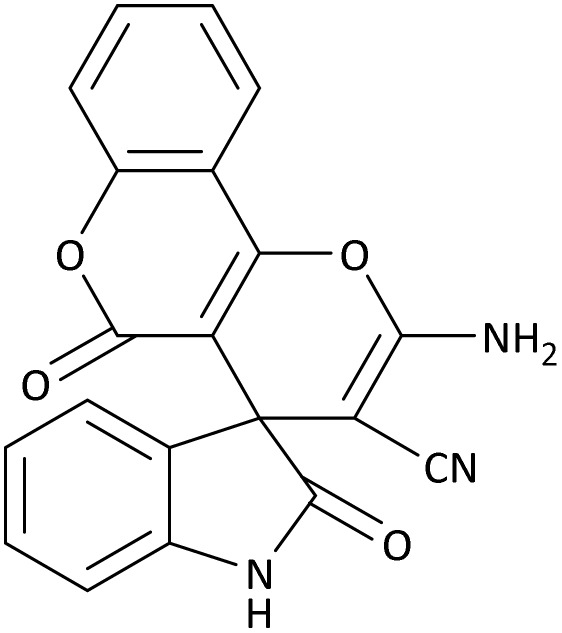	−1227.12032668	0.13812
5	3c	2b	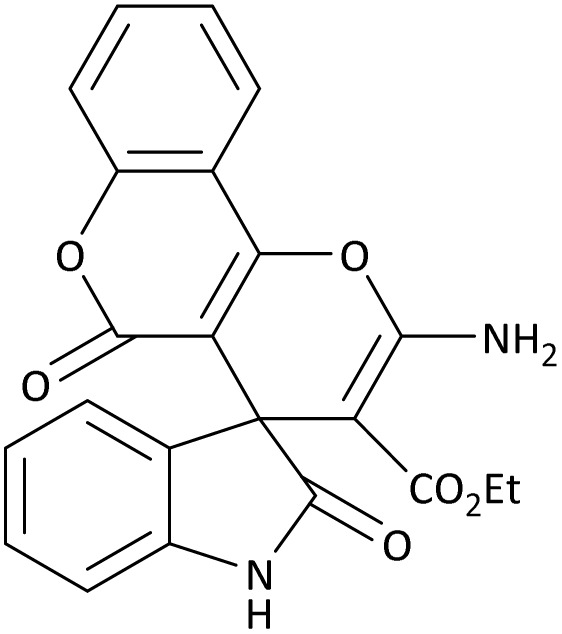	−1201.12599336	0.07286
6	3d	2a	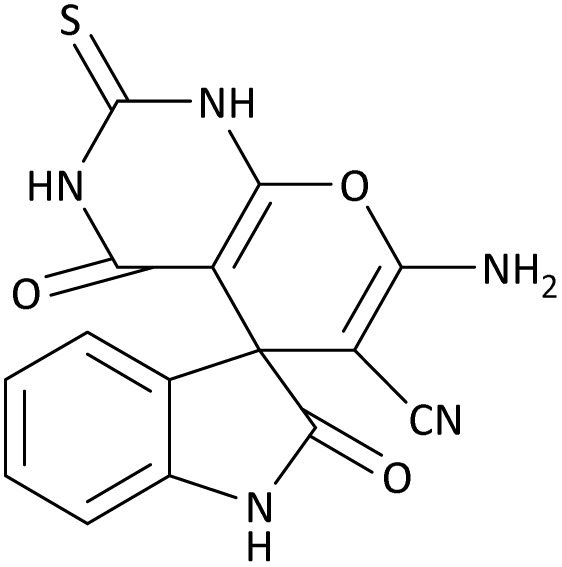	−1465.41619165	0.17037
7	3e	2a	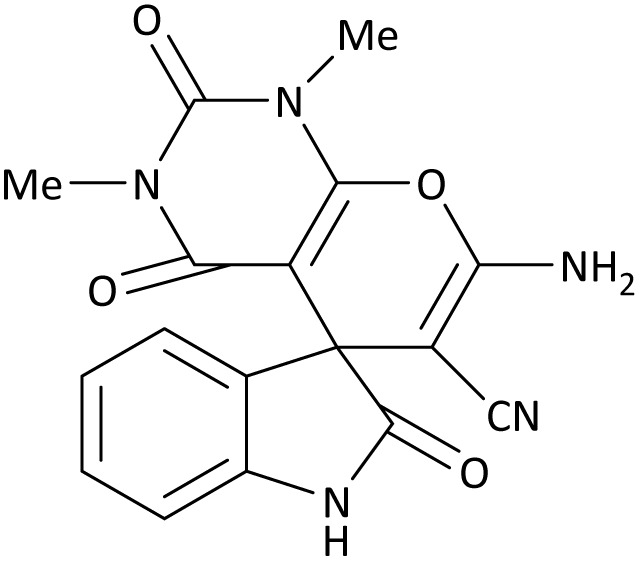	−1123.55900915	0.07046

**Fig. 11:**
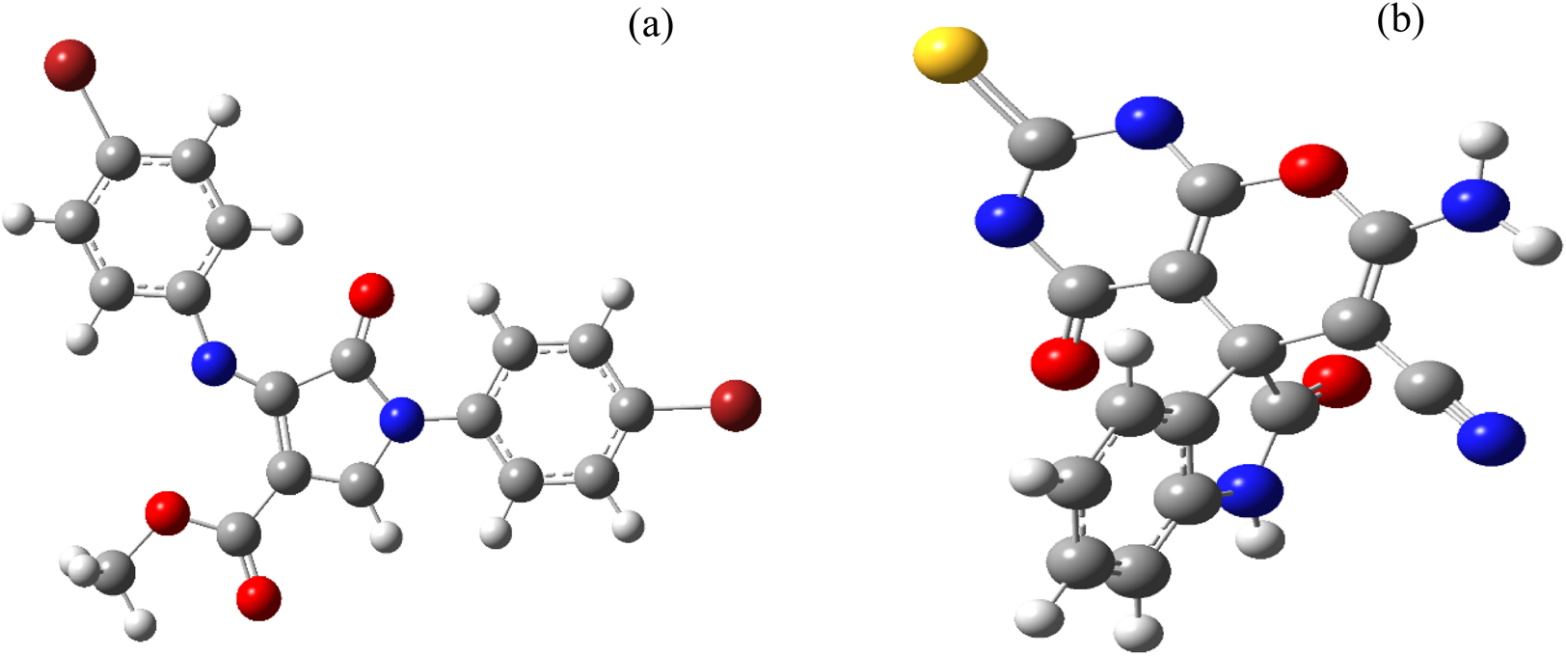
The relaxed structures for (a) compound 3 in dihydro-2-oxopyrrole derivatives and (b) compound 6 in spirooxindoles.

Furthermore, Fig. 11 displays the optimized structures of compound 3 in dihydro-2-oxopyrrole derivatives and compound 6 in spirooxindoles with total energies of −6185.36730055 and −1465.41619165 a.u., respectively.

Based on the electronic band gap energy, total energy, and relaxed structures, we conclude that compound 3 in dihydro-2-oxopyrrole derivatives and compound 6 in spirooxindoles are more stable than the other reported compounds. This finding aligns well with the experimental data.

## Experimental section

### Materials and methods

Chemicals were purchased from Merck, Fluka, and Aldrich Chemical Companies. ^1^H NMR and ^13^C NMR spectra were recorded at 400 and 100 MHz, respectively. Fourier transform infrared (FT-IR) measurements (in KBr pellets or ATR) were recorded on a Bruker spectrometer. Melting points were determined on a Büchi B-540 apparatus. The X-ray diffraction (XRD) pattern was obtained by a Philips Xpert MPD diffractometer equipped with a Cu Kα anode (*k* = 1.54 Å) in the 2*θ* range from 10° to 80°. Field Emission Scanning Electron Microscopy (FESEM) was obtained on a Mira 3-XMU. Transmission electron microscopy (TEM) was obtained using a Philips CM120 with a LaB6 cathode and an accelerating voltage of 120 kV. Energy-dispersive X-ray spectroscopy (EDS) of nano-catalyst was measured by an EDS instrument and Phenom pro-X. The EDX-MAP micrographs were obtained on the MIRA II detector SAMX (France). Thermal gravimetric analysis (TGA) was conducted using the “STA 504” instrument. BELSORP MINI II nitrogen adsorption apparatus (Japan) for recording Brunauer–Emmett–Teller (BET) of nano-catalyst at 77 K. The Reactions were conducted using the Mixer Mill model Retsch MM 400 which consisted of two stainless steel vials, each containing two stainless steel balls.

### MoO_3_/BF_3_ preparation

To prepare MoO_3_/BF_3_ nano-catalyst, boron trifluoride (BF_3_·Et_2_O) (0.251 mL, 1 mmol) was added drop by drop to molybdic acid (0.08 g, 1.5 mmol) in a crystalline mortar under the hood. The resulting mixture was grinding for 1 hour. After the completion of the reaction (formation of a blue precipitate), the resulting mixture was dried several times with diethyl ether at ambient temperature and used in the required reactions.

### Synthesis of spirooxindole derivatives *via* ballmilling

In a stainless steel mixer mill vial, a mixture of different 1,3-diketones (1 mmol), malononitrile or ethyl cyanoacetate (1 mmol), and isatin (1 mmol) in the presence of MoO_3_/BF_3_ (0.01 g) nano-catalyst was milled at room temperature at a frequency of 20 Hz. The reaction proceeded at a suitable time until completion. The reaction was monitored by thin-layer chromatography (*n*-hexane : ethyl acetate, 1 : 3). After the end of the reaction, hot ethanol was added and the reaction mixture was scraped and filtered to separate the nano-catalyst. Then cold water was added to the reaction mixture. The residue appears as a solid.

### Synthesis of dihydro-2-oxopyrrole derivatives *via* ballmilling

In a stainless steel mixer mill vial, a mixture of different DAAD (1 mmol), aromatic aniline (2 mmol), and formaldehyde (1.5 mmol) in the presence of MoO_3_/BF_3_ (0.04 g) was milled at room temperature at a frequency of 20 Hz. The reaction was monitored by thin-layer chromatography (*n*-hexane : ethyl acetate, 1 : 3). After the completion of the reaction, hot ethanol was added and the reaction mixture was scraped and filtered to separate the nano-catalyst by simple filtration. In the end, cold water was added to the obtained solution.

## Conclusions

In this study, the MoO_3_/BF_3_ nano-catalyst was successfully prepared and characterized by different analyses such as FT-IR, XRD, FESEM, TEM, EDX, EDS-MAP, BET, and TGA. Briefly, the results concluded that the MoO_3_/BF_3_ could be considered as a desired catalyst with high acid properties (pH = 1) for the synthesis of nitrogen-containing heterocyclic compounds, such as spirooxindole and dihydro-2-oxopyrrole, using a mill mixer, which is a mechanochemical, green, and economic method. The hot filtration test of the nano-catalyst was performed, and it shows that the present nano-catalyst is heterogeneous and there was no leakage into the reaction mixture. The main advantages of this method are green conditions, short reaction time, high acidity, high reaction efficiency, and recyclability without a significant decrease in its activity. The DFT simulations indicate compound 6 in spirooxindoles and compound 3 in dihydro-2-oxopyrrole derivatives exhibit greater stability than other reported compounds. These findings are consistent with the experimental data.

## Author contributions

DM, BFM, HB, and AB designed and performed the research, analyzed the data, interpreted the results, and prepared the manuscript. DM performed the assay and optimized and purified the compounds. HB performed the DFT calculation of compounds. All authors read and approved the final manuscript.

## Conflicts of interest

There are no conflicts to declare.

## Supplementary Material

RA-015-D5RA01991E-s001

## Data Availability

All data generated or analysed during this study are included in this published article.
