# Supplementary material for: B(iii)-catalyzed synthesis of spirooxindole and dihydro-2-oxopyrrole under solventless conditions in a ball mill, along with DFT computations
Source: RSC Adv. 2025 Jul 21;15(32):25949–64. doi: 10.1039/d5ra01991e (PMC12278902; doi:10.1039/d5ra01991e)
Supplement: RA-015-D5RA01991E-s001 [file RA-015-D5RA01991E-s001.pdf]

Supplementary Information (SI) for RSC Advances.
This journal is © The Royal Society of Chemistry 2025

B(III)-catalyzed synthesis of spirooxindole and dihydro-2-oxopyrrole under solventless conditions in a ball mill, along with DFT computations

Dina Mallah[a], Bi Bi Fatemeh Mirjalili*[b], Hadi Basharnavaz*[b], and Abdolhamid Bamoniri[c]

[1]Department of Chemistry, College of Science, Yazd University, P.O. Box 89195-741, Yazd, I.R.IRAN. Fax: +983538210644; Tel: +983531232672. Email: fmirjalili@yazd.ac.ir

[2]Department of Organic Chemistry, Faculty of Chemistry, University of Kashan, Kashan, I.R.IRAN.

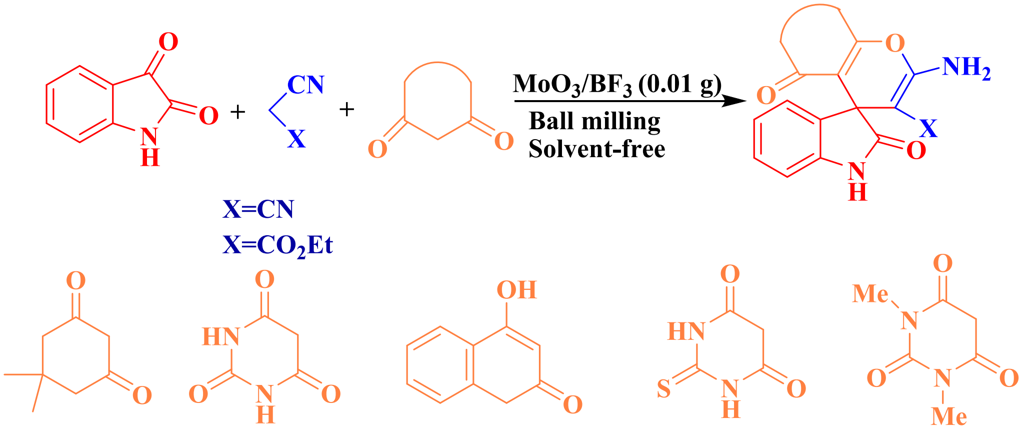

2-Amino-7,7-dimethyl-2′,5-dioxo-5,6,7,8-tetrahydrospiro[chromene-4,3′-indoline]-3-carbon itrile.

White solid. m.p. 290-292 ºC. FT-IR (ATR) ῡ (cm^{-1}): 3302, 3134, 2955, 2191, 1720, 1681, 1654, 1604 1466, 1347, 1216, 1053, 903, 743, 677. ^{1}H NMR (400 MHz, *DMSO-d_6*) /δ (ppm): 0.99 (s, 3H, CH_3), 1.02 (s, 3H, CH_3), 2.06–2.18 (m, 2H, CH_2), 2.49–2.55 (m, 2H, CH_2), 6.78 (d, J = 7.6 Hz, 1H), 6.90 (t, J = 7.2 Hz, 1H), 6.98 (d, J = 7.2 Hz, 1H), 7.15 (t, J = 7.6 Hz, 1H), 7.22 (s, 2H, NH_2), 10.38 (s, 1H, NH).

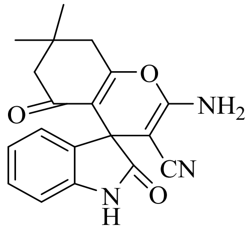

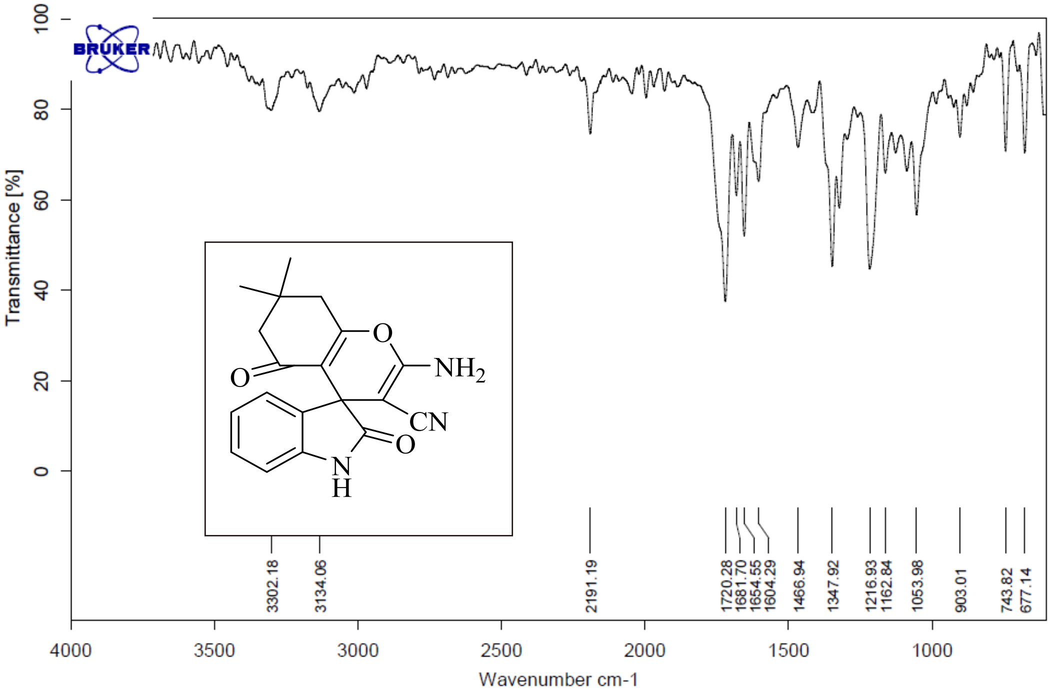

The FT-IR of

2-Amino-7,7-dimethyl-2′,5-dioxo-5,6,7,8-tetrahydrospiro[chromene-4,3′-indoline]-3-carbon itrile

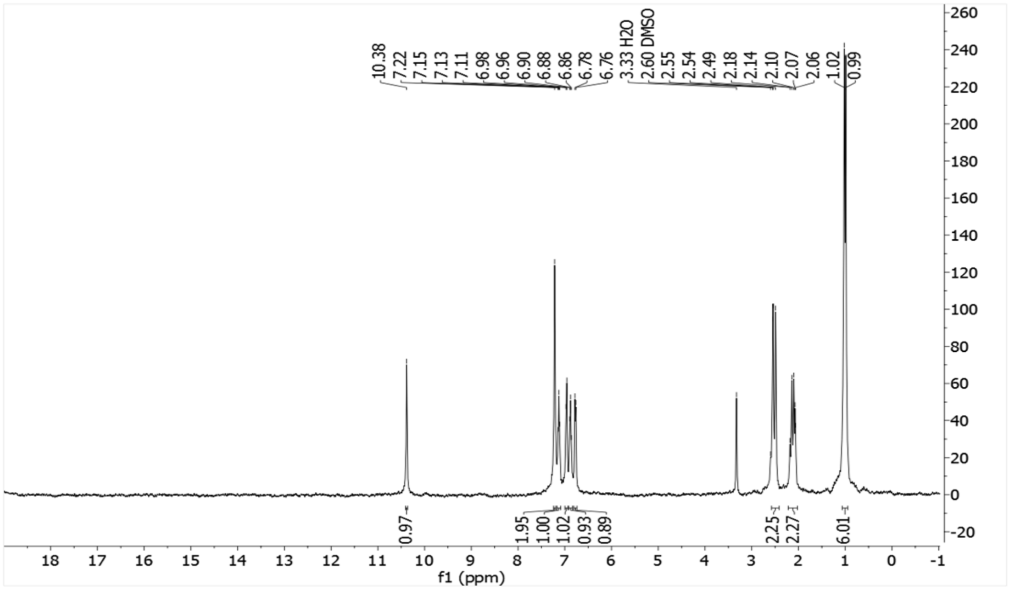

The ^{1}H NMR spectrum of

2-Amino-7,7-dimethyl-2′,5-dioxo-5,6,7,8-tetrahydrospiro[chromene-4,3′-indoline]-3-carbon itrile

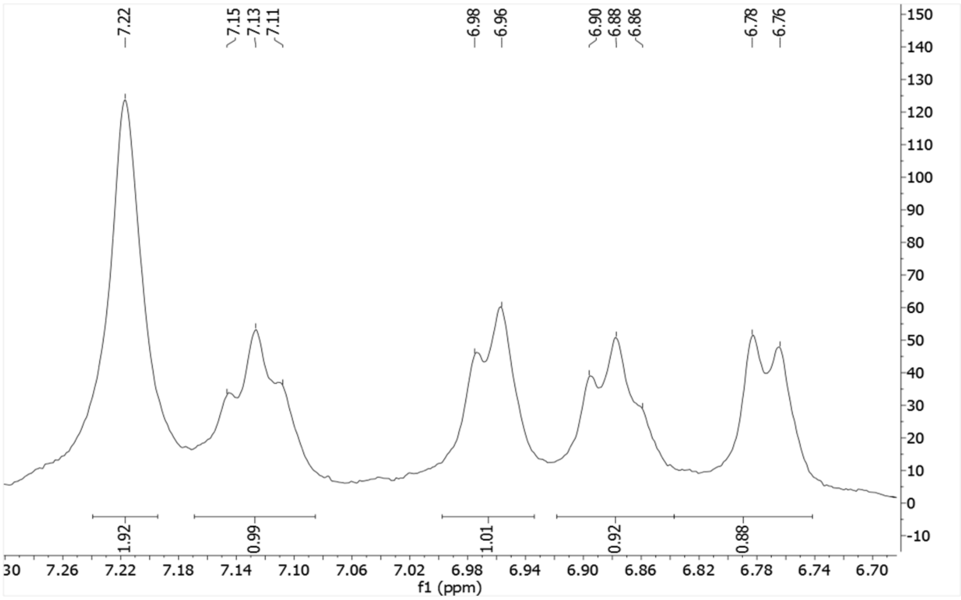

The ^{1}H NMR spectrum of

2-Amino-7,7-dimethyl-2′,5-dioxo-5,6,7,8-tetrahydrospiro[chromene-4,3′-indoline]-3-carbonitrile

Ethyl-2-amino-7,7-dimethyl-2′,5-dioxo-5,6,7,8-tetrahydrospiro[chromene-4,3′-indoline]-3-carboxylate.

White solid. m.p. 230-232 ºC. FT-IR (ATR) ῡ (cm^{-1}): 3366, 3175, 3109, 2960, 1711, 1685, 1613, 1525, 1473, 1397, 1222, 1057, 909, 745, 674 . ^{1}H NMR (500 MHz, *DMSO-d$_6$*) /δ (ppm): 0.81 (t, 3H, CH_3),0.94 (s, 3H, CH_3), 1.01 (s, 3H, CH_3), 2.03 (d, J = 16.0 Hz, 1H), 2.16 (d, J = 16.0 Hz, 1H), 2.50-2.51 (m, 2H, CH_2), 3.67-3.74 (m, 2H, CH_2), 6.68 (d, J = 7.5 Hz, 1H), 6.77 (t, J = 7.5 Hz, 1H), 6.84 (d, J = 7.5 Hz, 1H), 7.05 (t, J = 7.5 Hz, 1H), 7.85 (s, 2H, NH_2), 10.13 (s, 1H, NH). ^{13}C NMR (125 MHz, *DMSO-d$_6$*) /δ (ppm): 13, 27, 31, 46, 51, 70, 76, 106, 113, 126, 136, 144, 157, 159, 162, 167, 177, 179, 182, 194.

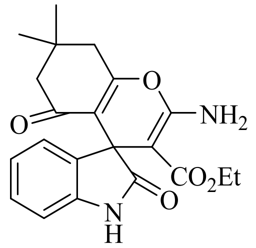

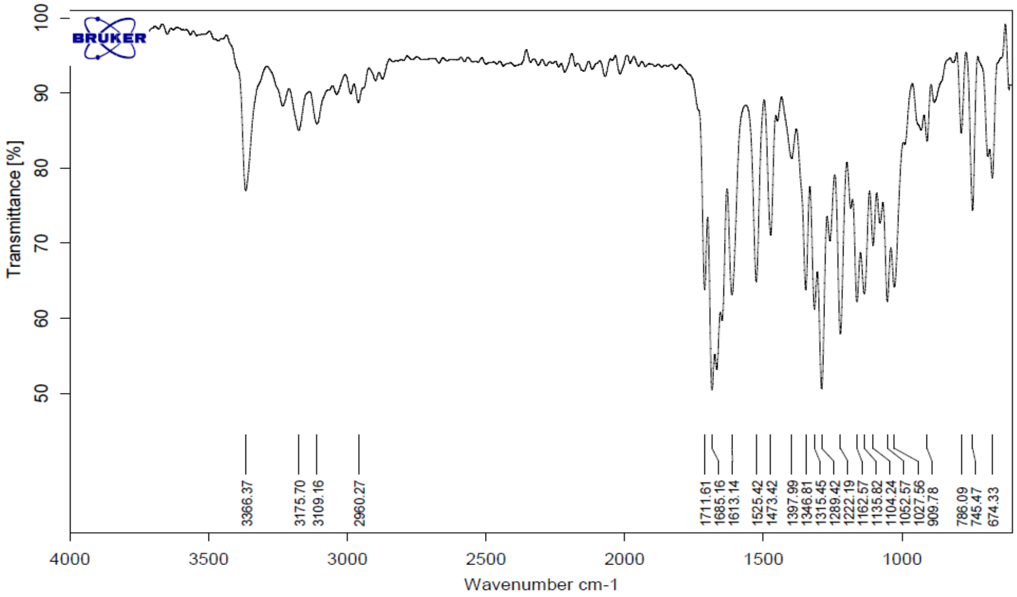

The FT-IR of Ethyl-2-amino-7,7-dimethyl-2′,5-dioxo-5,6,7,8-tetrahydrospiro[chromene-4,3′-indoline]-3-carboxylate

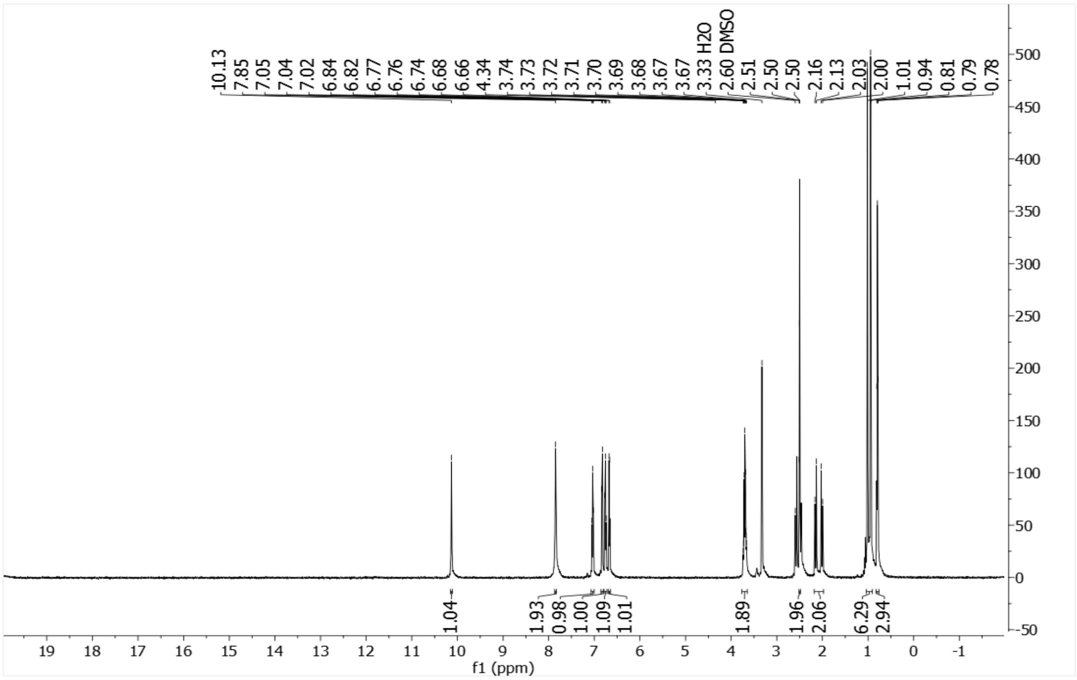

The ^{1}H NMR spectrum of Ethyl-2-amino-7,7-dimethyl-2′,5-dioxo-5,6,7,8-tetrahydrospiro[chromene-4,3′-indoline]-3-carboxylate

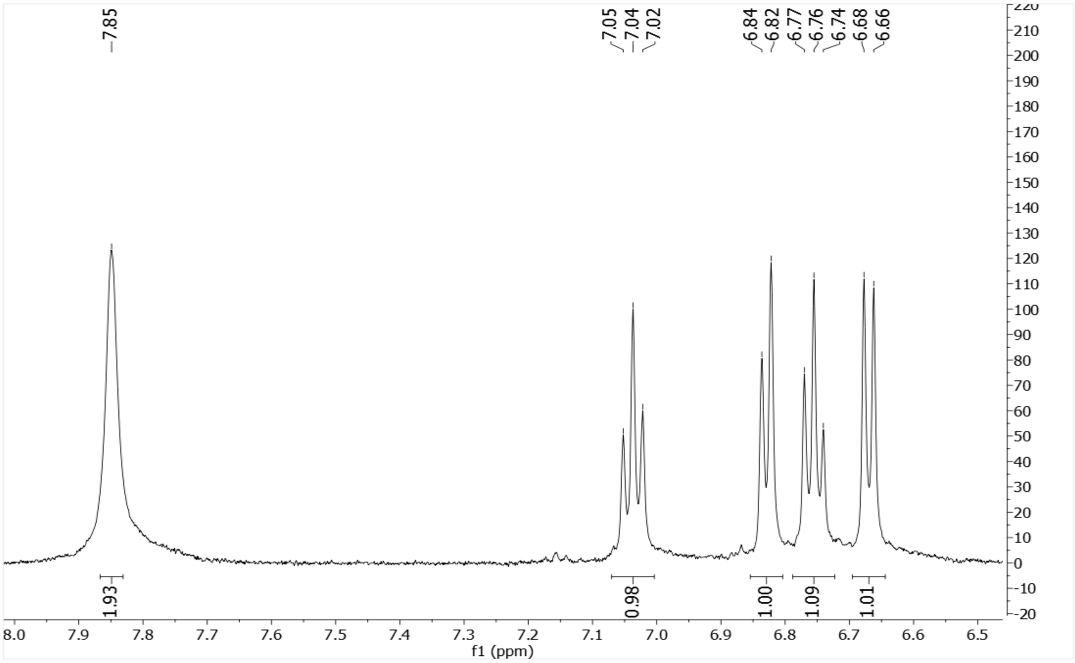

The ^{1}H NMR spectrum of Ethyl-2-amino-7,7-dimethyl-2′,5-dioxo-5,6,7,8-tetrahydrospiro[chromene-4,3′-indoline]-3-carboxylate

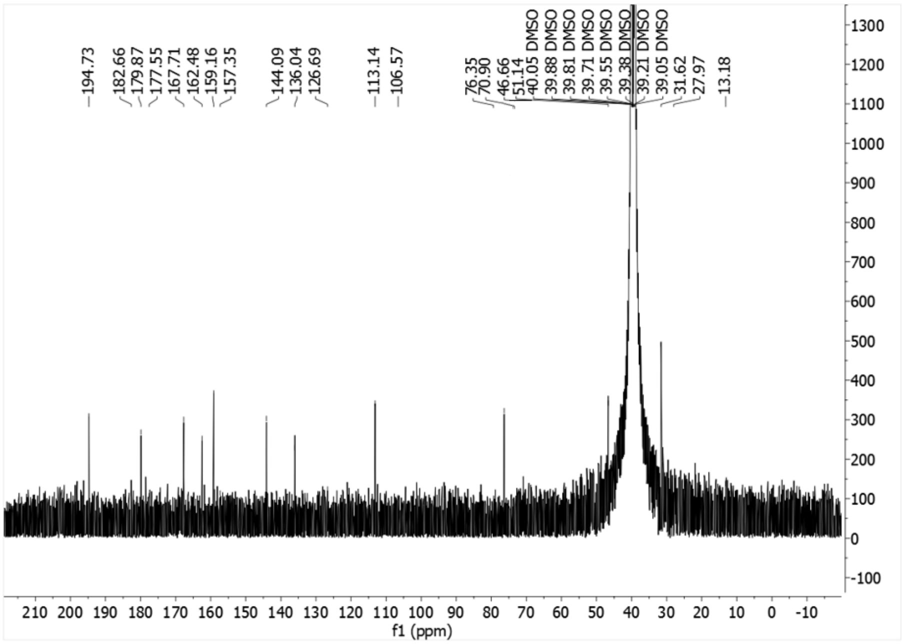

The ^{13}C NMR spectrum of Ethyl-2-amino-7,7-dimethyl-2′,5-dioxo-5,6,7,8-tetrahydrospiro[chromene-4,3′-indoline]-3-carboxylate

7'-Amino-2,2',4'-trioxo-1',2',3',4'-tetrahydrospiro[indoline-3,5'-pyrano[2,3-*d*]pyrimidine]-6'-carbonitrile.

White solid. m.p. 270-272 ºC. FT-IR (ATR) ῡ (cm^{-1}): 3467, 3303, 3157, 2202, 1677, 1614, 1528, 1439. ^1H NMR (500 MHz, *DMSO-d$_6$*) /δ (ppm): 6.79 (s, *J* = 6.5 Hz, 1H), 6.92 (t, *J* = 7.0 Hz, 1H), 7.12-7.17 (m, 2H), 7.37 (s, 2H, NH_2), 10.48 (s, 1H, NH), 11.13 (s, 1H, NH), 12.30 (s, 1H, NH). ^{13}C NMR (125MHz, *DMSO-d$_6$*) /δ (ppm): 46, 57, 86, 116, 121, 123, 128, 138, 149, 153, 157, 158, 161, 177.

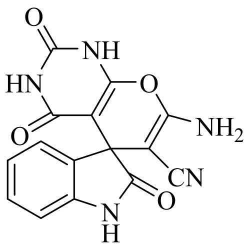

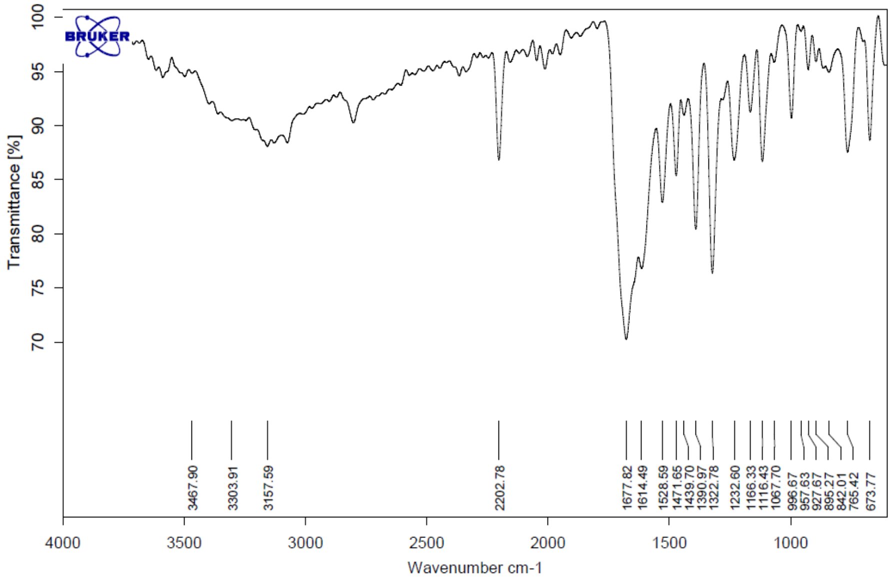

The FT-IR of 7'-Amino-2,2',4'-trioxo-1',2',3',4'-tetrahydrospiro[indoline-3,5'-pyrano[2,3-*d*]pyrimidine]-6'-carbonitrile

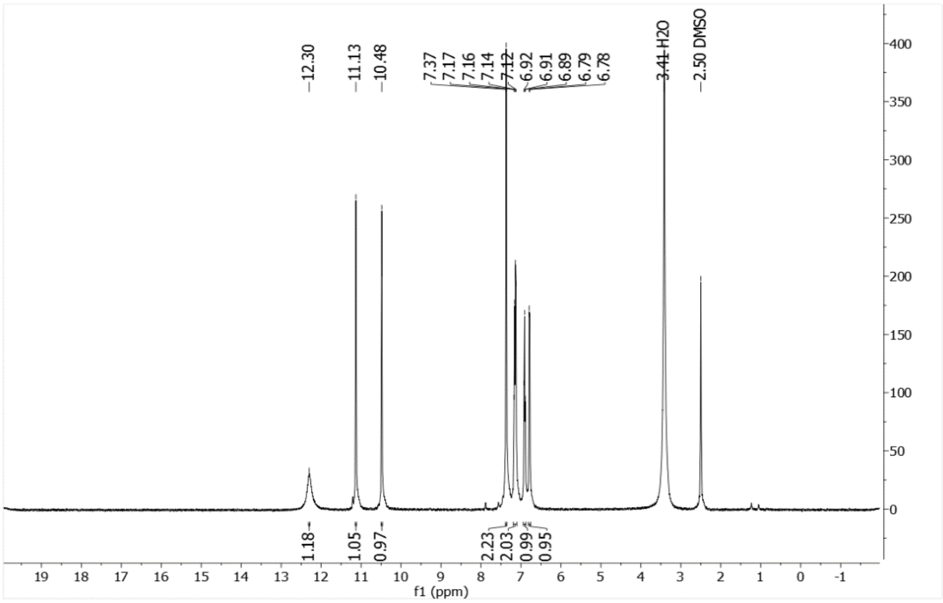

The ^{1}H NMR spectrum of 7'-Amino-2,2',4'-trioxo-1',2',3',4'-tetrahydrospiro[indoline-3,5'-pyrano[2,3-*d*]pyrimidine]-6'-carbonitrile

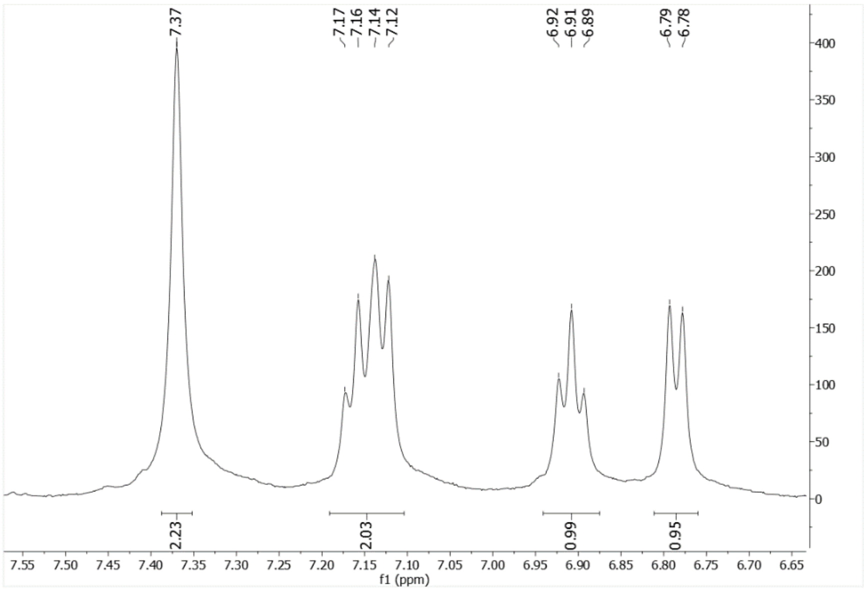

The ^{1}H NMR spectrum of 7'-Amino-2,2',4'-trioxo-1',2',3',4'-tetrahydrospiro[indoline-3,5'-pyrano[2,3-*d*]pyrimidine]-6'-carbonitrile

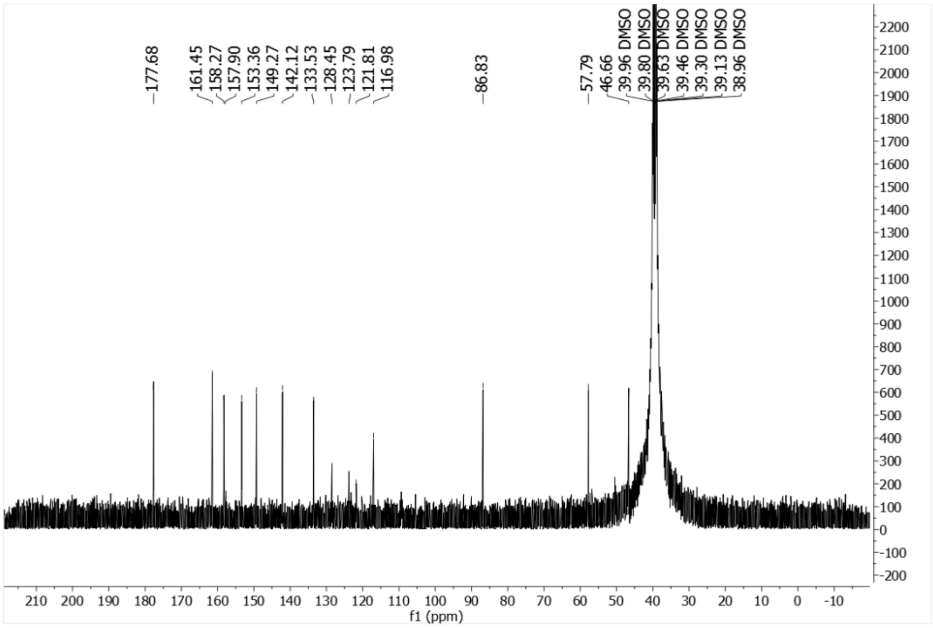

The ^{13}C NMR spectrum of 7'-Amino-2,2',4'-trioxo-1',2',3',4'-tetrahydrospiro[indoline-3,5'-pyrano[2,3-*d*]pyrimidine]-6'-carbonitrile

2′-Amino-2,5′-dioxo-5′*H*-spiro[indoline-3,4′-pyrano(3,2-*c*)chromen]-3′-carbonitrile.

White solid, m.p. 293-295 ºC. FT-IR (ATR) ῡ (cm^{-1}): 3355, 3289, 3195, 2199, 1706, 1667, 1601, 1523, 1470, 1355. ^{1}H NMR (500 MHz, *DMSO-d*$_6$) /δ (ppm): 6.87 (d, J = 8.0 Hz, 1H), 6.95 (t, J = 8.0 Hz, 1H), 7.20-7.23 (m, 2H), 7.50 (d, J = 8.0 Hz, 1H), 7.56 (t, J = 8.0 Hz, 1H), 7.66 (s, 2H, NH_2), 7.78 (t, J = 8.0 Hz, 1H), 7.95 (d, J = 7.5 Hz, 1H), 10.67 (s, 1H, NH). ^{13}C NMR (125 MHz, *DMSO-d*$_6$) /δ (ppm): 47, 57, 87, 101, 109, 112, 117, 122, 123, 124, 125, 128, 133, 134, 142, 152, 155, 158, 158, 177.

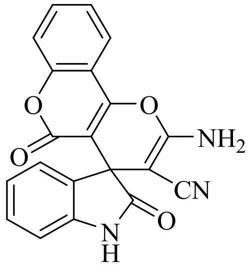

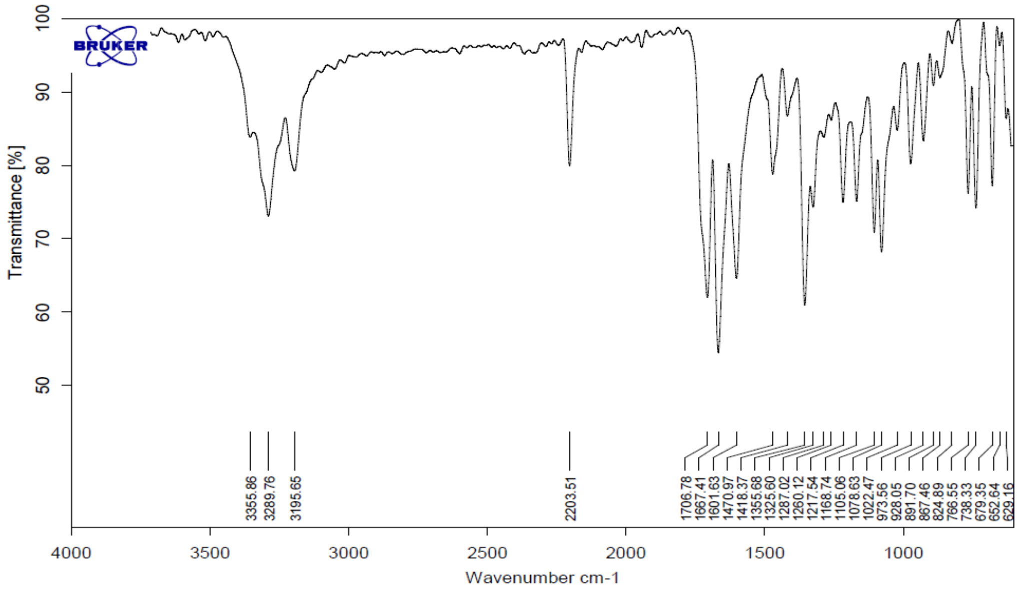

The FT-IR of 2′-Amino-2,5′-dioxo-5′*H*-spiro[indoline-3,4′-pyrano(3,2-*c*)chromen]-3′-carbonitrile

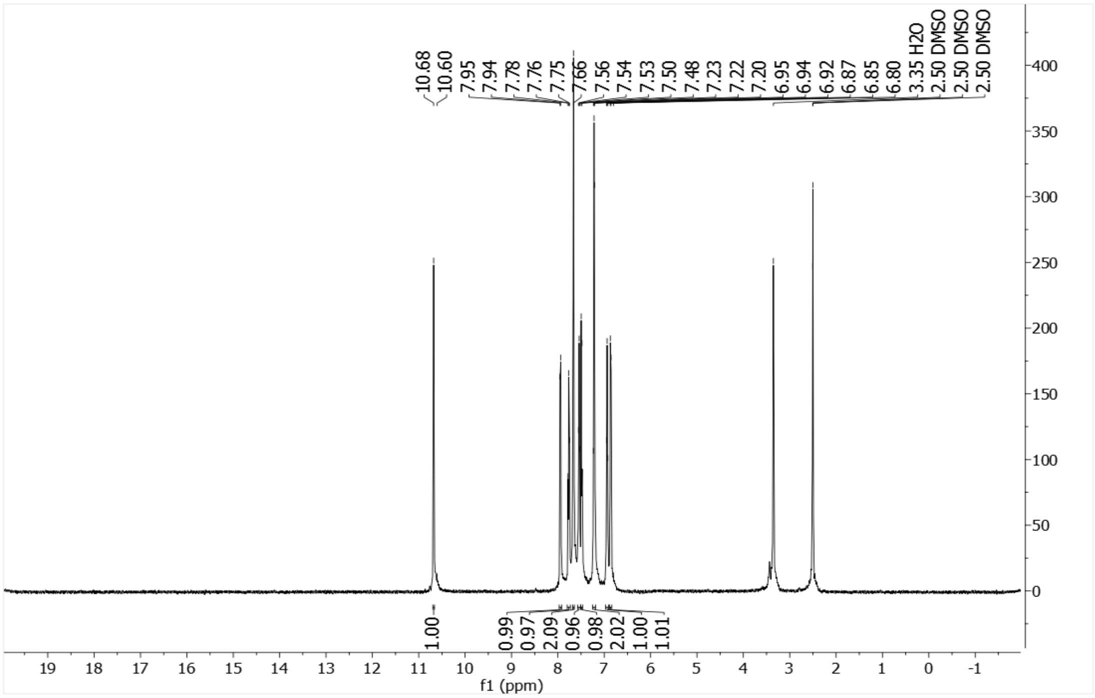

The ^{1}H NMR spectrum of 2′-Amino-2,5′-dioxo-5′*H*-spiro[indoline-3,4′-pyrano(3,2-*c*)chromen]- 3′-carbonitrile

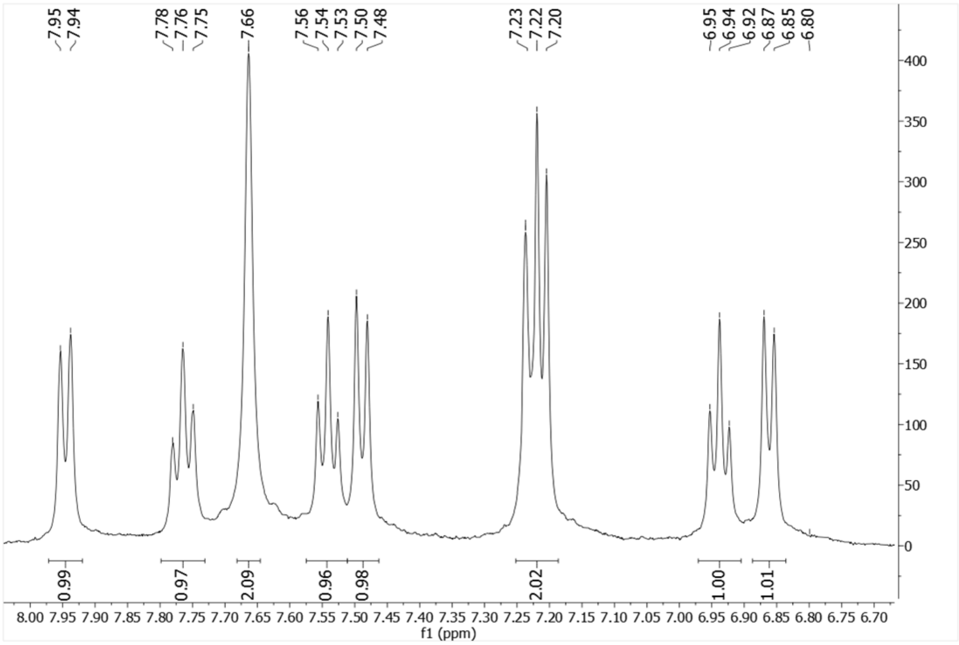

The ^{1}H NMR spectrum of 2′-Amino-2,5′-dioxo-5′*H*-spiro[indoline-3,4′-pyrano(3,2-*c*)chromen]- 3′-carbonitrile

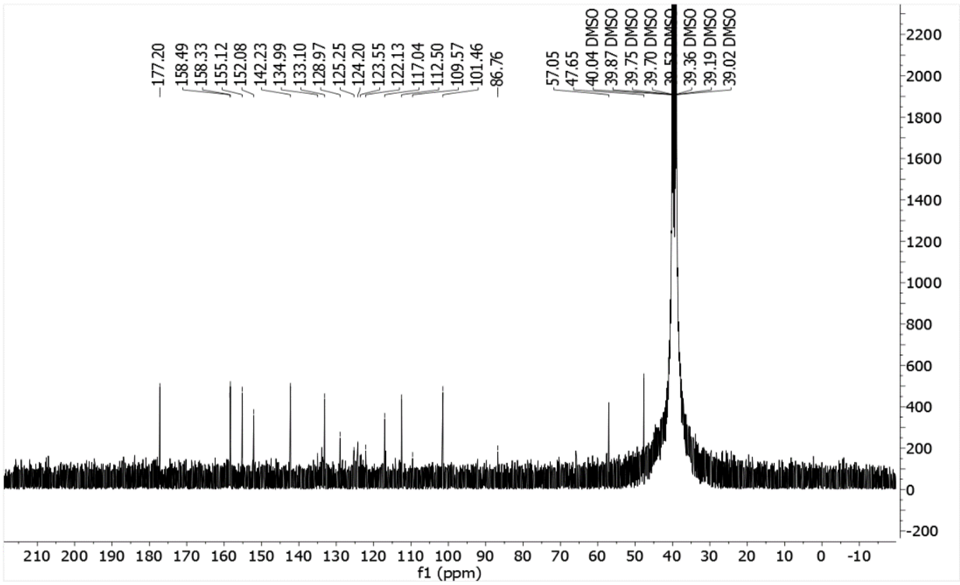

The ^{13}C NMR spectrum of 2′-Amino-2,5′-dioxo-5′*H*-spiro[indoline-3,4′-pyrano(3,2-*c*)chromen]- 3′-carbonitrile

Ethyl-2′-amino-2,5′-dioxo-5′*H*-spiro[indoline-3,4′-pyrano(3,2-*c*)chromen]-3′-carbonitrile.

White solid, m.p. 210-212 ºC. FT-IR (ATR) ῡ (cm^{-1}): 3471, 3380, 3058, 1712, 1653, 1612, 1513, 1472, 1354. ^{1}H NMR (500 MHz, *DMSO-d_6*) /δ (ppm): 0.85 (t, 3H, CH_3), 3.72- 3.80 (m, 2H, OCH_2), 6.76 (d, J = 7.5 Hz, 1H), 6.82 (t, J = 7.5 Hz, 1H), 7.02 (d, J = 7.0 Hz, 1H), 7.14 (t, J = 7.5 Hz, 1H), 7.44 (d, J = 8.5 Hz, 1H), 7.53 (t, J = 7.5 Hz, 1H), 7.75 (t, J = 8.5 Hz, 1H), 8.03 (d, J = 8.0 Hz, 1H), 8.14 (s, 2H, NH_2), 10.43 (s, 1H, NH). ^{13}C NMR (125MHz, *DMSO-d_6*) /δ (ppm): 13, 25, 37, 42, 47, 59, 75, 103, 112, 127, 134, 144, 151, 153, 157, 158, 167, 178, 195.

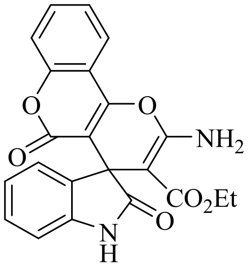

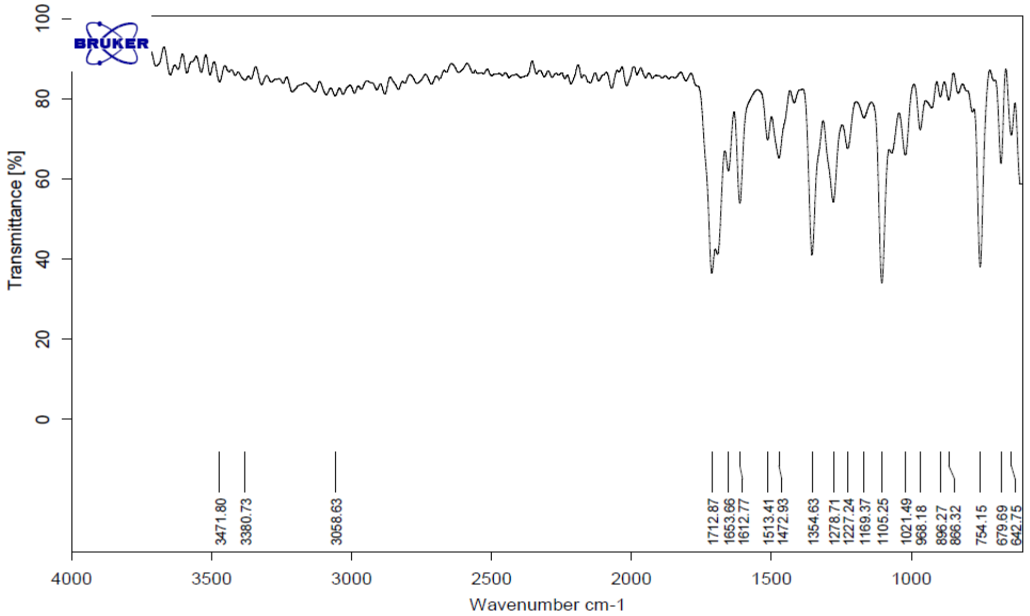

The FT-IR of Ethyl-2′-amino-2,5′-dioxo-5′*H*-spiro[indoline-3,4′-pyrano(3,2-*c*)chromen]-3′-carbonitrile

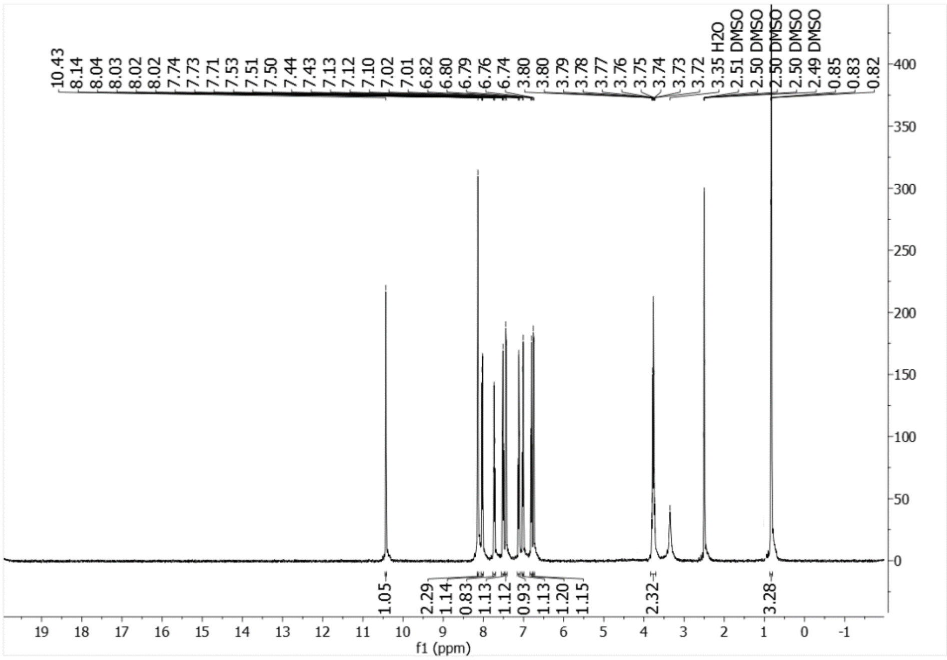

The ^{1}H NMR spectrum of Ethyl-2′-amino-2,5′-dioxo-5′*H*-spiro[indoline-3,4′-pyrano(3,2-*c*)chromen]-3′-carbonitrile

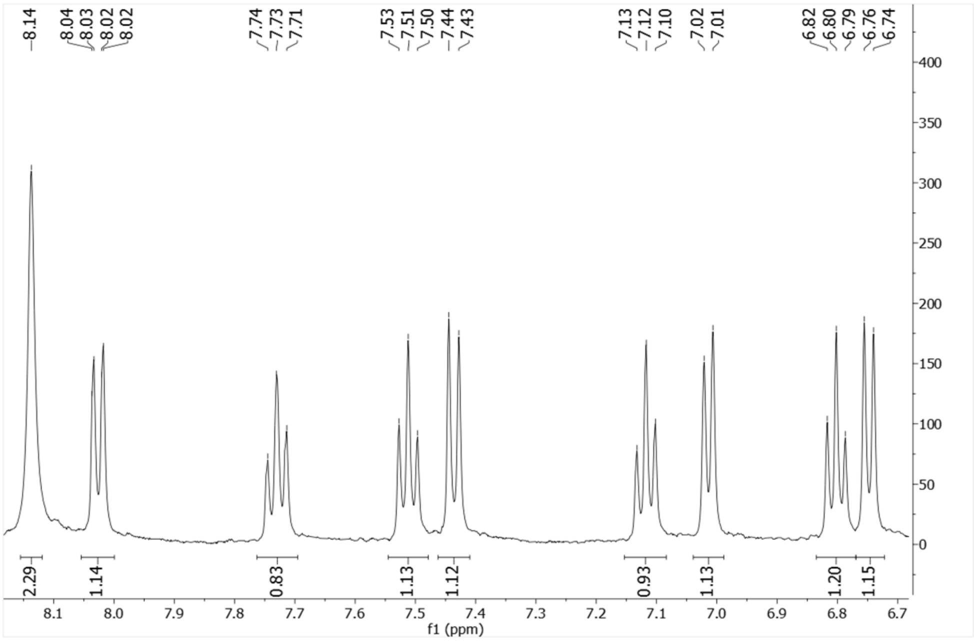

The ^{1}H NMR spectrum of Ethyl-2′-amino-2,5′-dioxo-5′*H*-spiro[indoline-3,4′-pyrano(3,2-*c*)chromen]-3′-carbonitrile

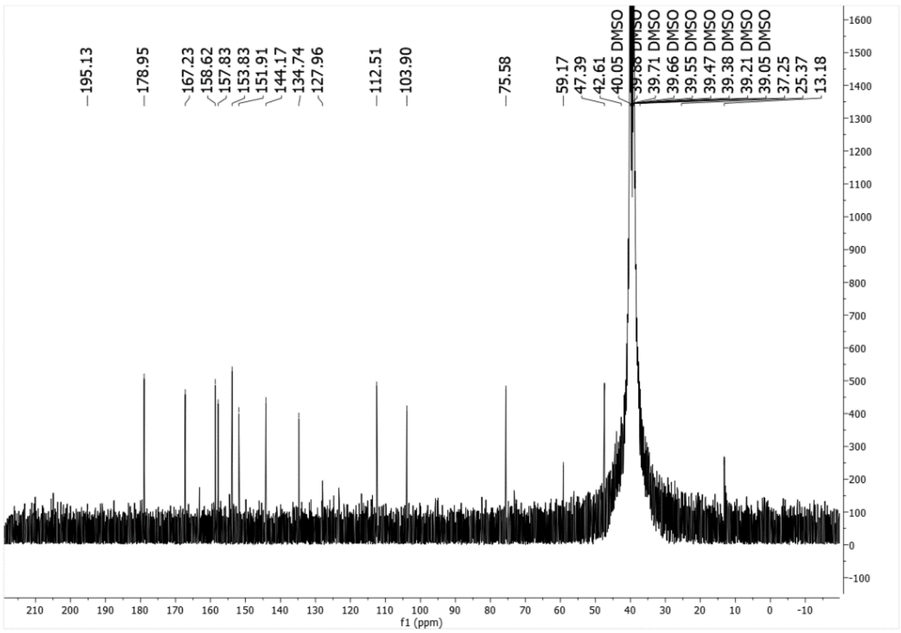

The ^{13}C NMR spectrum of Ethyl-2′-amino-2,5′-dioxo-5′*H*-spiro[indoline-3,4′-pyrano(3,2-*c*)chromen]-3′-carbonitrile

7′-Amino-2,4′-dioxo-2′-thioxo-1′,2′,3′,4′-tetrahydrospiro[indoline-3,5′-pyrano(2,3-*d*)pyrimidine]-6′-carbonitrile.

White solid, m.p. 244-246 ºC. FT-IR (ATR) ῡ (cm^{-1}): 3427, 3310, 3159, 2200, 1686, 1653, 1567, 1467, 1399, 1340. ^{1}H NMR (500 MHz, *DMSO-d$_6$*) /δ (ppm): 6.80 (d, *J* = 8.0 Hz, 1H), 6.93 (t, *J* = 7.5 Hz, 1H), 7.16-7.20 (m, 2H), 7.42 (s, 2H, NH_2), 10.54 (s, 1H, NH), 11.20 (s, NH, 1H), 12.50 (s, 1H, NH). ^{13}C NMR (125MHz, *DMSO-d$_6$*) /δ (ppm): 46, 57, 91, 116, 124, 128, 132, 142, 150, 152, 158, 159, 173, 177.

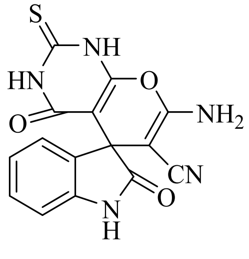

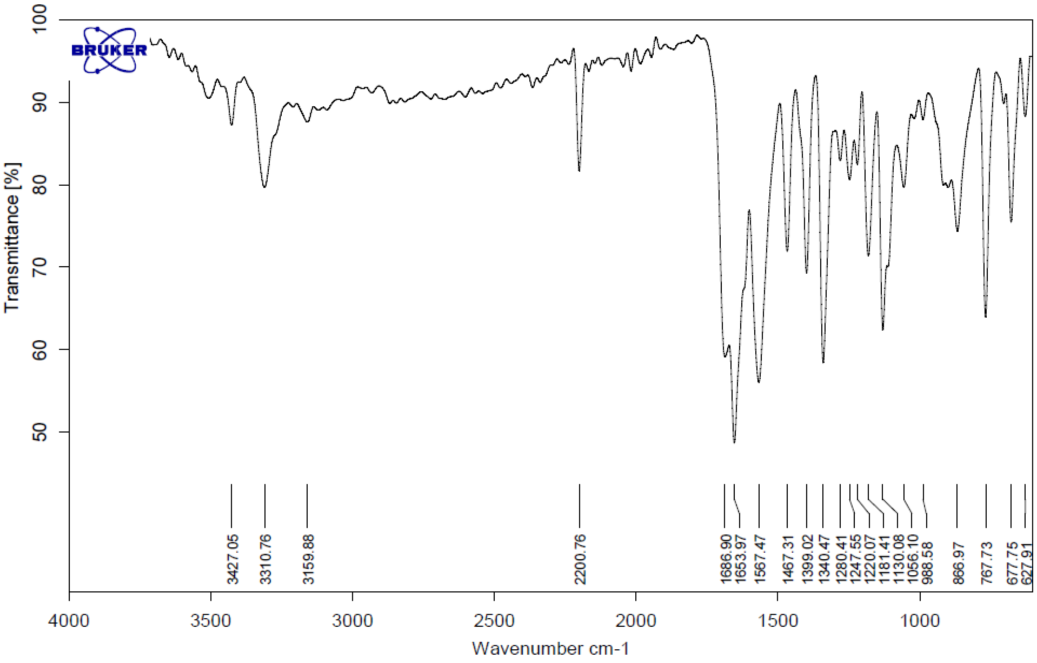

The FT-IR of 7′-Amino-2,4′-dioxo-2′-thioxo-1′,2′,3′,4′-tetrahydrospiro[indoline-3,5′-pyrano(2,3-*d*)pyrimidine]-6′-carbonitrile

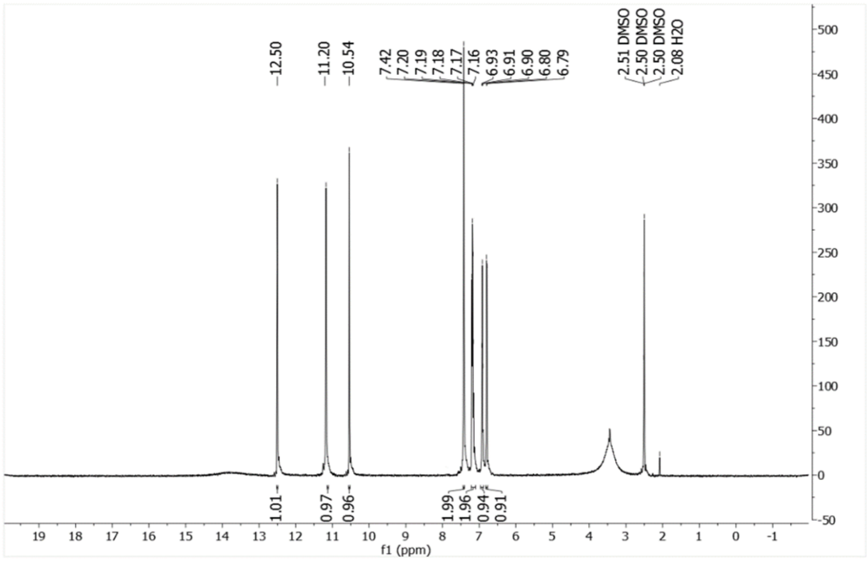

The ^{1}H NMR spectrum of 7′-Amino-2,4′-dioxo-2′-thioxo-1′,2′,3′,4′-tetrahydrospiro[indoline-3,5′-pyrano(2,3-*d*)pyrimidine]-6′-carbonitrile

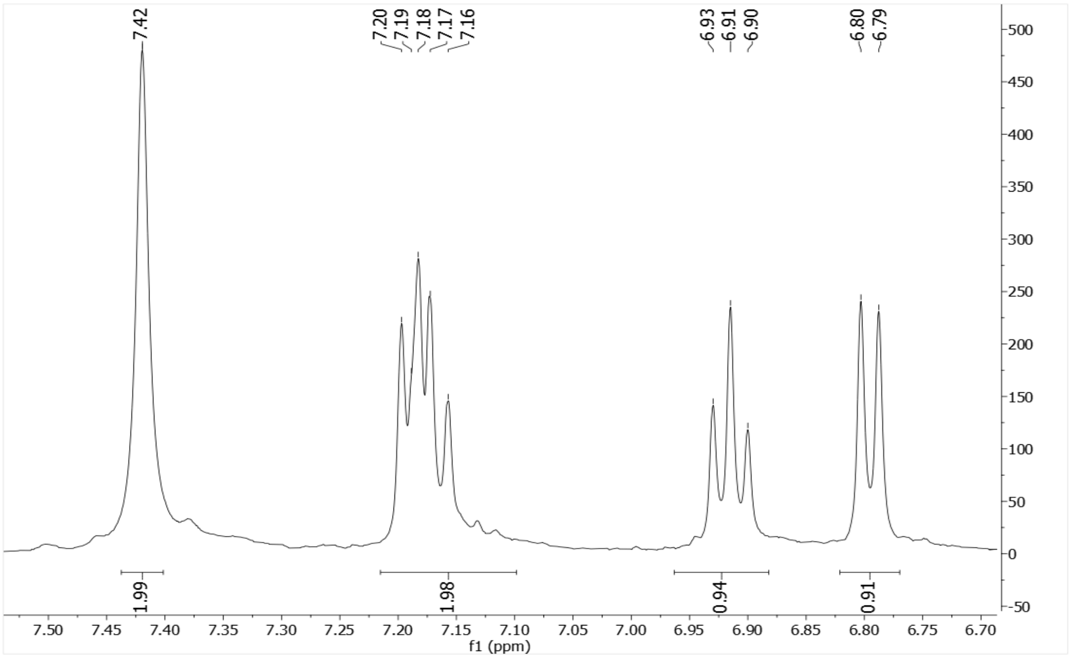

The ^{1}H NMR spectrum of 7′-Amino-2,4′-dioxo-2′-thioxo-1′,2′,3′,4′-tetrahydrospiro[indoline-3,5′-pyrano(2,3-*d*)pyrimidine]-6′-carbonitrile

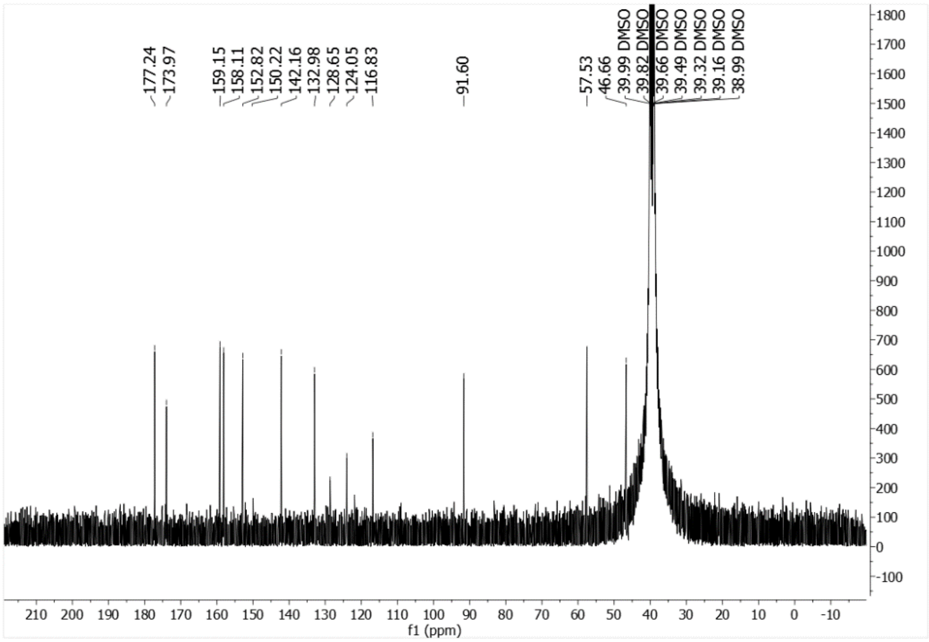

The ^{13}C NMR spectrum of 7′-Amino-2,4′-dioxo-2′-thioxo-1′,2′,3′,4′-tetrahydrospiro[indoline-3,5′-pyrano(2,3-*d*)pyrimidine]-6′-carbonitrile

7'-Amino-1',3'-dimethyl-2,2',4'-trioxo-1',2',3',4'-tetrahydrospiro[indoline-3,5'-pyrano[2,3-*d*]pyrimidine]-6'-carbonitrile.

White solid, m.p. 221-224 ºC. FT-IR (ATR) ῡ (cm^{-1}): 3310, 3184, 2200, 1706,1667, 1619, 1505, 1336, 1185. ^{1}H NMR (500 MHz, *DMSO-d_6*) /δ (ppm): 3.02 (s, 3H), 3.38 (s, 3H), 6.81 (d, J = 7.5 Hz, 1H), 6.92 (t, J = 8.0 Hz 1H), 7.13 (d, J = 8.0 Hz, 1H),7.18 (t, J = 7.5 Hz 1H), 7.56 (s, 2H, NH_2), 10.50 (s, 1H, NH). ^{13}C NMR (125 MHz, *DMSO-d_6*) /δ (ppm): 27, 29, 47, 57, 87, 110, 116, 123, 128, 133, 142, 149, 152, 158, 159, 177.

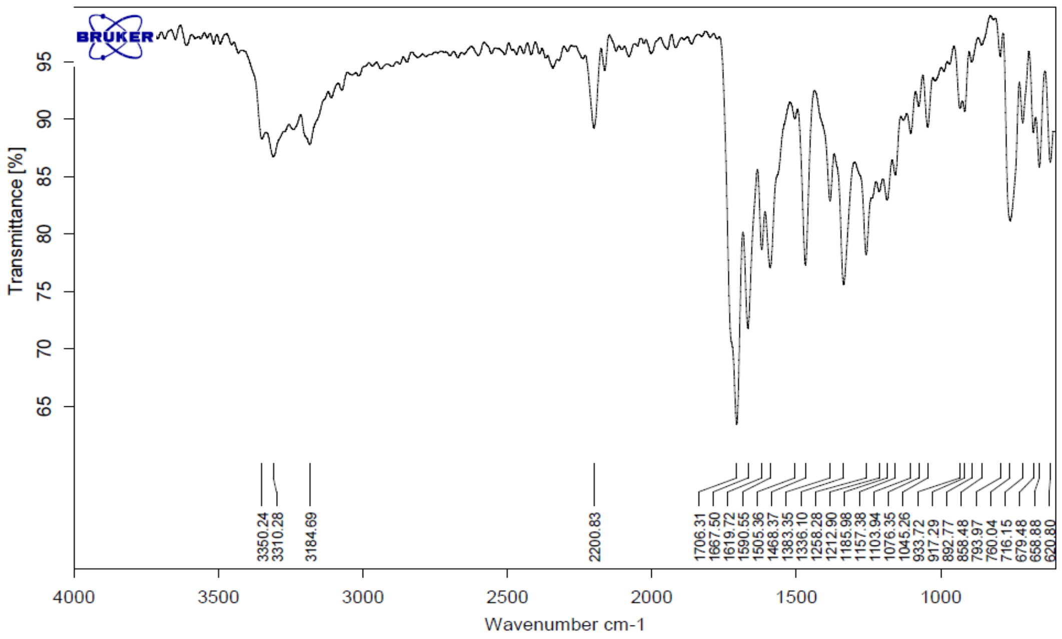

The FT-IR of 7'-Amino-1',3'-dimethyl-2,2',4'-trioxo-1',2',3',4'-tetrahydrospiro[indoline-3,5'-pyrano[2,3-*d*]pyrimidine]-6'-carbonitrile

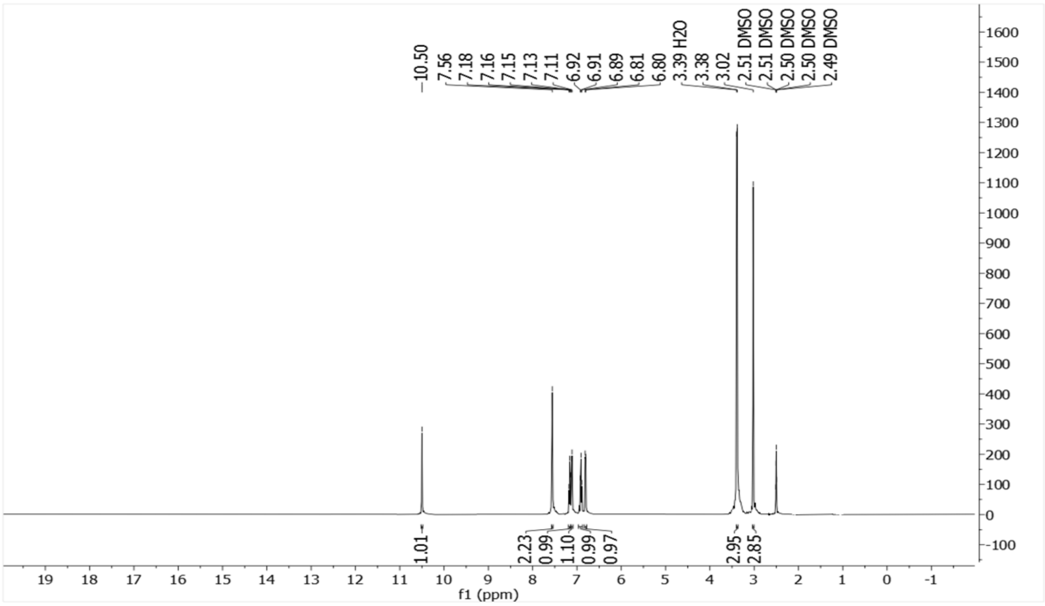

The ^{1}H NMR spectrum of 7'-Amino-1',3'-dimethyl-2,2',4'-trioxo-1',2',3',4'-tetrahydrospiro[indoline-3,5'-pyrano[2,3-*d*]pyrimidine]-6'-carbonitrile

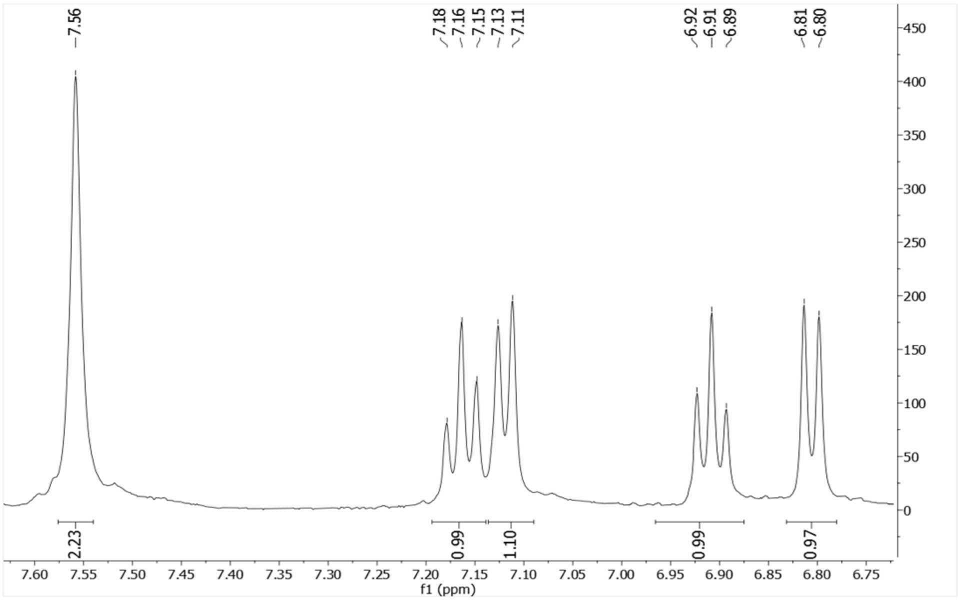

The ^{1}H NMR spectrum of 7'-Amino-1',3'-dimethyl-2,2',4'-trioxo-1',2',3',4'-tetrahydrospiro[indoline-3,5'-pyrano[2,3-*d*]pyrimidine]-6'-carbonitrile

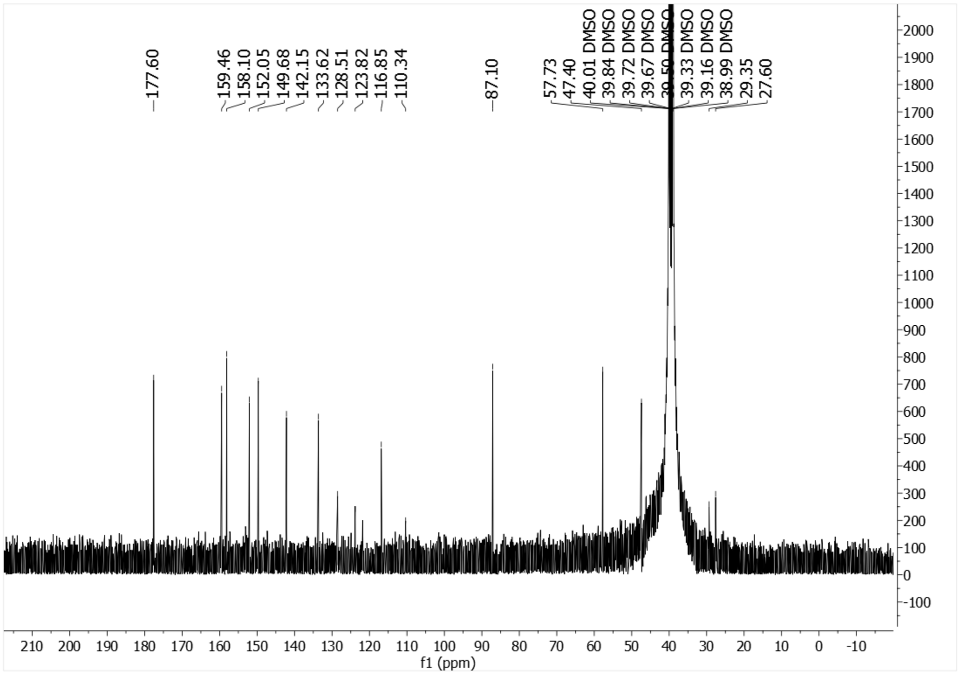

The ^{13}C NMR spectrum of 7'-Amino-1',3'-dimethyl-2,2',4'-trioxo-1',2',3',4'-tetrahydrospiro[indoline-3,5'-pyrano[2,3-*d*]pyrimidine]-6'-carbonitrile

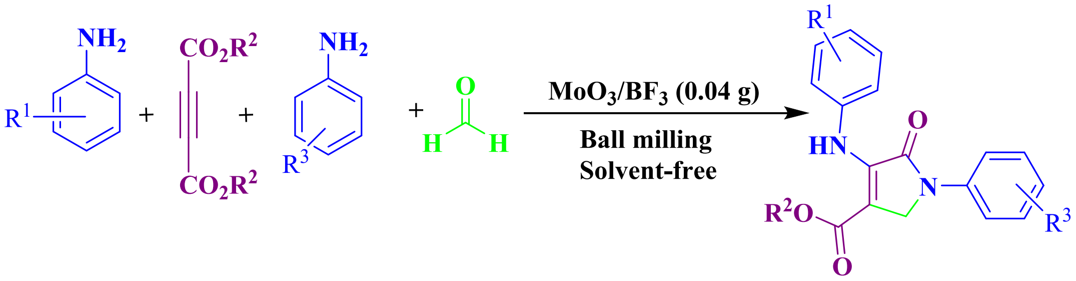

Methyl-1-(4-chlorophenyl)-4-((4-chlorophenyl)amino)-5-oxo-2,5-dihydro-1H-pyrrole-3-carboxylate

Cream solid. M. p. 173-174 °C. FT-IR (KBr)/ ῡ(cm^{-1}): 3280, 1671, 1645, 1591, 1532, 1495, 1439, 1395, 1289, 1224, 1150, 1093, 829. ^{1}H NMR ($CDCl_3$, 400 MHz)/δ ppm: 8.05 (br, s, 1H, NH), 7.73 (d, 2H, 3*J*= 6.4 Hz, Ar–H), 7.35 (s, 3H, Ar–H), 7.27 (d, 1H, 3*J*= 7.6 Hz, Ar–H), 7.07 (s, 2H, Ar–H), 4.50 (s, 2H, NCH_2), 3.78 (s, 3H, OCH_3). ^{13}C NMR ($CDCl_3$, 100 MHz)/δ ppm: 164.7, 163.5,

142.9, 137.1, 136.9, 130.3, 130.1, 129.2 (2C), 128.4 (2C), 124.1 (2C), 120.2 (2C), 103.6, 51.5, 48.0.

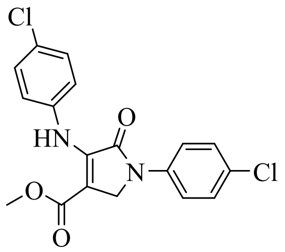

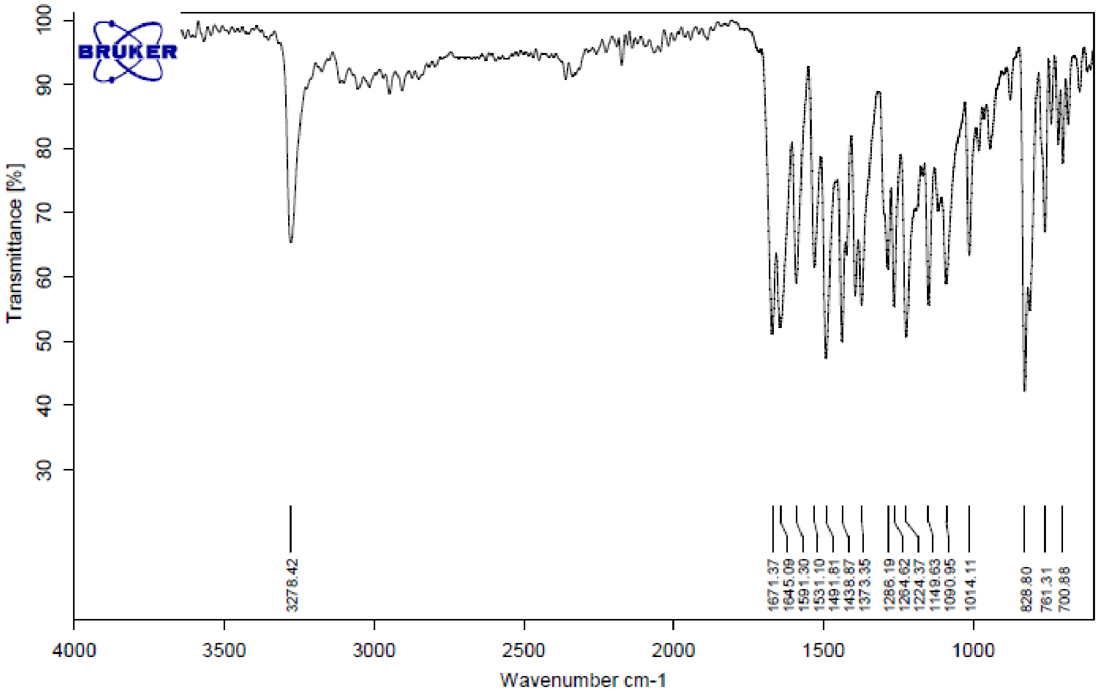

The FT-IR of methyl-1-(4-chlorophenyl)-4-((4-chlorophenyl)amino)-5-oxo-2,5-dihydro-1H-pyrrole-3-carboxylate

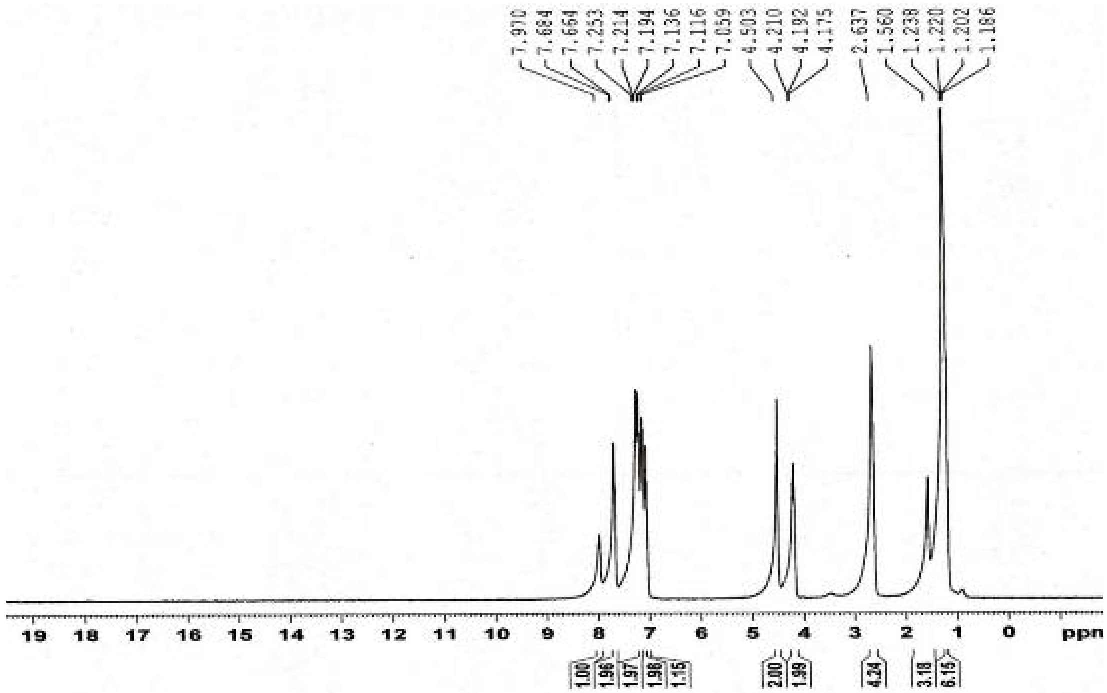

The ^{1}H NMR spectrum of methyl-1-(4-chlorophenyl)-4-((4-chlorophenyl)amino)-5-oxo-2,5-dihydro-1H-pyrrole-3-carboxylate

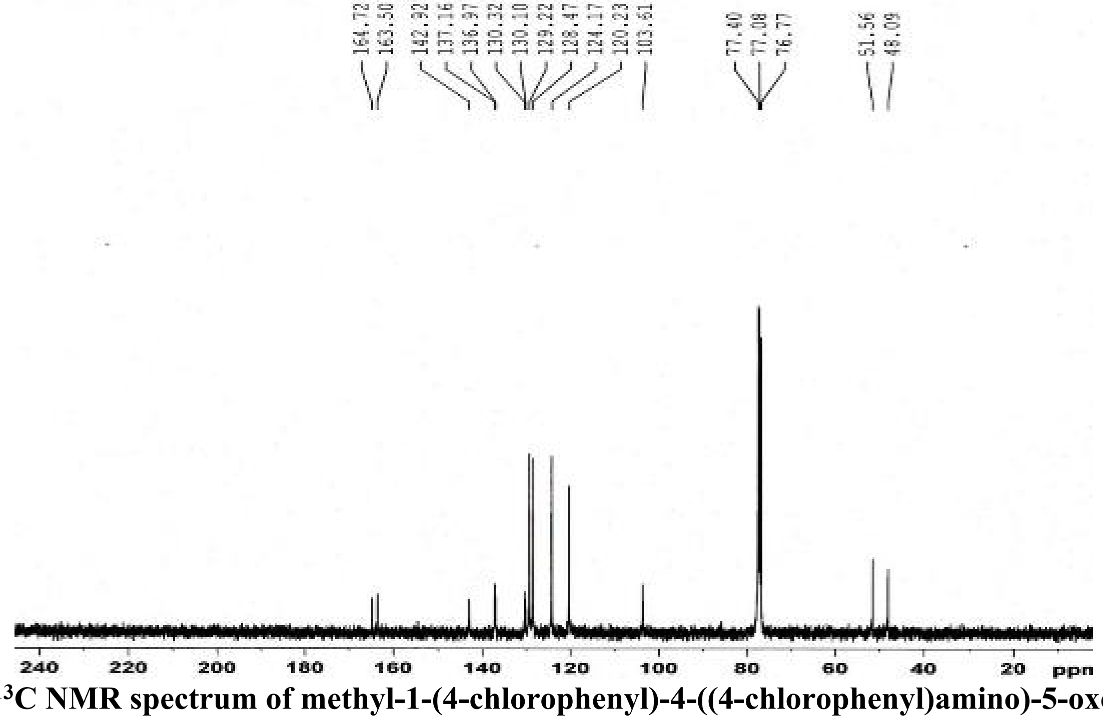

The ^{13}C NMR spectrum of methyl-1-(4-chlorophenyl)-4-((4-chlorophenyl)amino)-5-oxo-2,5-dihydro-1H-pyrrole-3-carboxylate

Ethyl-1-(4-chlorophenyl)-4-((4-chlorophenyl)amino)-5-oxo-2,5-dihydro-1H-pyrrole-3-carboxylate

White solid. M. p. 165-167 °C. FT-IR (KBr)/ ῡ(cm^{-1}): 3308, 1691, 1639, 1595, 1492, 1456, 1384, 1197, 822. ^{1}H NMR ($CDCl_3$, 400 MHz)/δ ppm: 8.04 (s, 1H, NH), 7.74 (d, 2H, ^3J= 8.8 Hz, Ar–H), 7.35 (d, 2H, ^3J= 7.6 Hz, ^3J= 8.8 Hz, Ar–H), 7.27 (d, 2H, ^3J= 7.6 Hz, Ar–H), 7.06 (d, 2H, 3*J*= 8.4 Hz, Ar–H), 4.50 (s, 2H, NCH_2), 4.24 (q, 3H, 3*J*= 7.2 Hz, OCH2CH3), 1.26 (t, 3H, ^3J= 7.2 Hz, OCH_2CH_3). ^{13}C NMR ($CDCl_3$, 100 MHz)/δ ppm: 164.4, 163.6, 142.7, 137.2, 137.1, 130.3, 130.0, 129.2 (2C), 128.5 (2C), 124.0 (2C), 120.3 (2C), 104.1, 60.6, 48.1, 14.0.

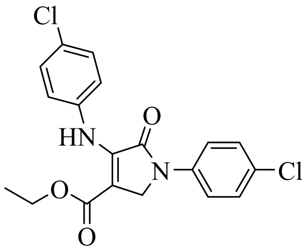

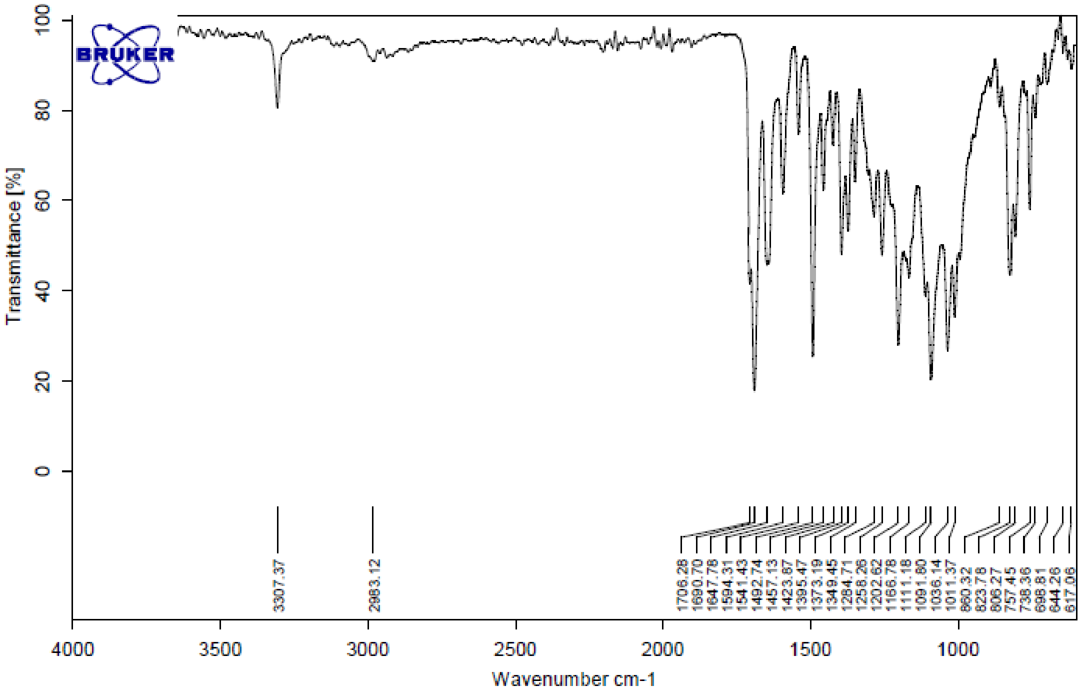

The FT-IR of ethyl-1-(4-chlorophenyl)-4-((4-chlorophenyl)amino)-5-oxo-2,5-dihydro-1H-pyrrole-3-carboxylate

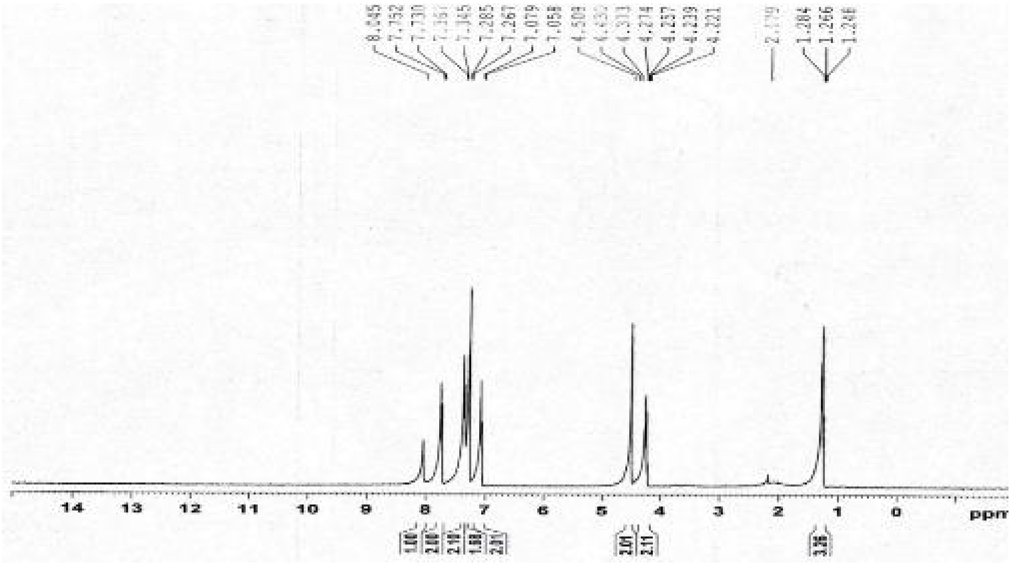

The ^{1}H NMR spectrum of ethyl-1-(4-chlorophenyl)-4-((4-chlorophenyl)amino)-5-oxo-2,5-dihydro-1H-pyrrole-3-carboxylate

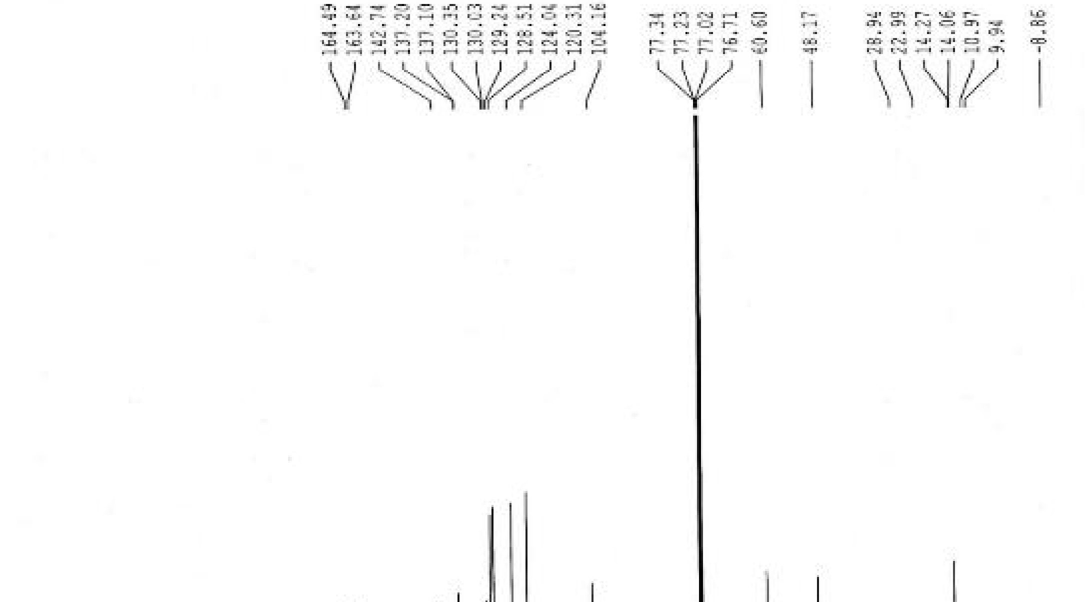

The ^{13}C NMR spectrum of ethyl-1-(4-chlorophenyl)-4-((4-chlorophenyl)amino)-5-oxo-2,5-dihydro-1H-pyrrole-3-carboxylate

Methyl-1-(4-bromophenyl)-4-((4-bromophenyl)amino)-5-oxo-2,5-dihydro-1H-pyrrole-3-carboxylate

White solid. M. p. 181-182 °C. FT-IR (KBr)/ ῡ(cm^{-1}): 3309, 1699, 1687, 1637, 1587, 1487, 1381, 1192, 815. ^{1}H NMR ($CDCl_3$, 400 MHz)/δ ppm: 8.02 (br, s, 1H, NH), 7.66 (d, 2H, ^{3}J= 9.2 Hz, Ar–H), 7.49 (d, 3H, ^{3}J= 8.8 Hz, Ar–H), 7.41 (d, 1H, ^{3}J= 8.4 Hz, Ar–H), 6.99 (d, 2H, ^{3}J= 8.0 Hz, Ar–H), 4.48 (s, 2H, NCH_2), 3.77 (s, 3H, OCH_3). ^{13}C NMR ($CDCl^3$, 100 MHz)/δ ppm: 164.7, 163.5, 142.8, 137.6, 137.4, 132.1 (2C), 131.4 (2C), 124.4 (2C), 120.5 (2C), 118.0, 117.8, 103.8, 51.5, 48.03.

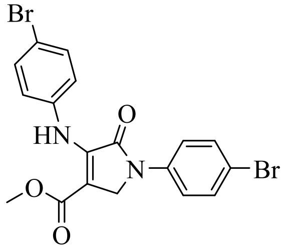

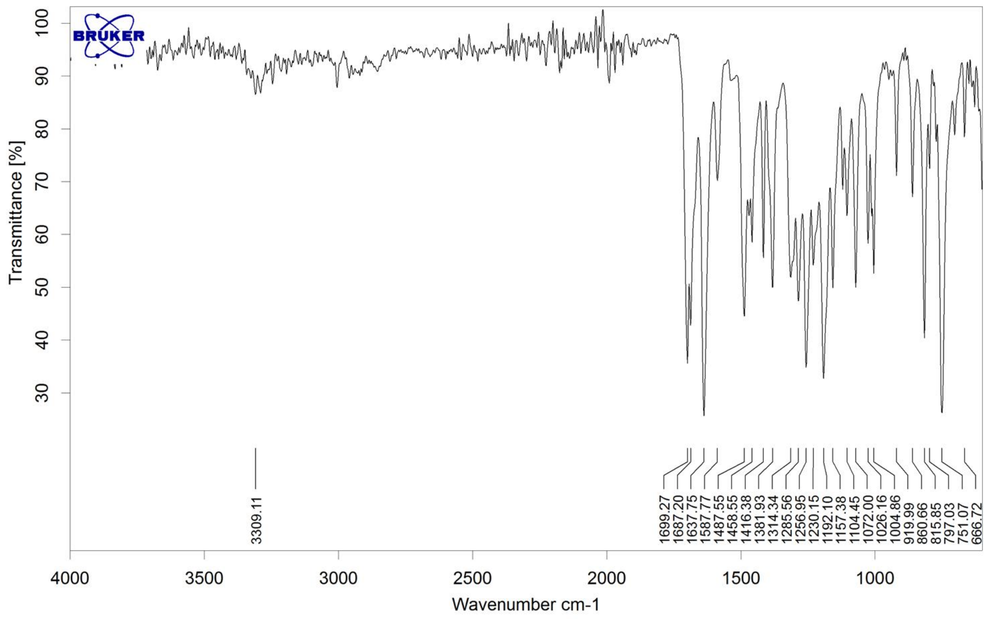

The FT-IR of methyl-1-(4-bromophenyl)-4-((4-bromophenyl)amino)-5-oxo-2,5-dihydro-1H-pyrrole-3-carboxylate

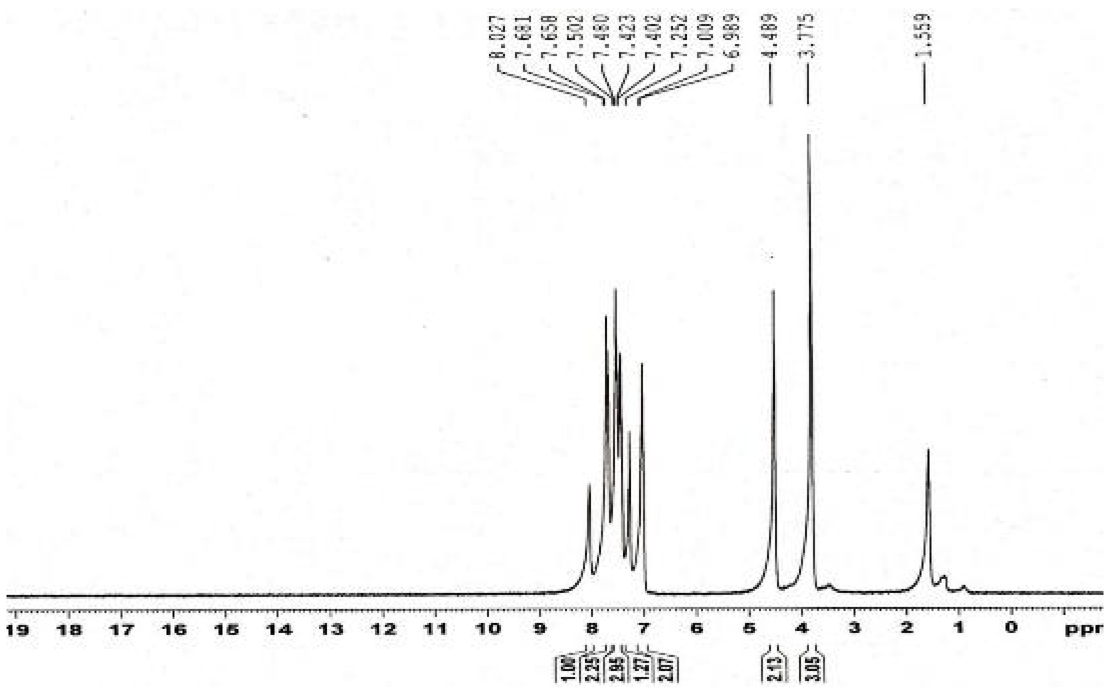

The ^{1}H NMR spectrum of methyl-1-(4-bromophenyl)-4-((4-bromophenyl)amino)-5-oxo-2,5-dihydro-1H-pyrrole-3-carboxylate

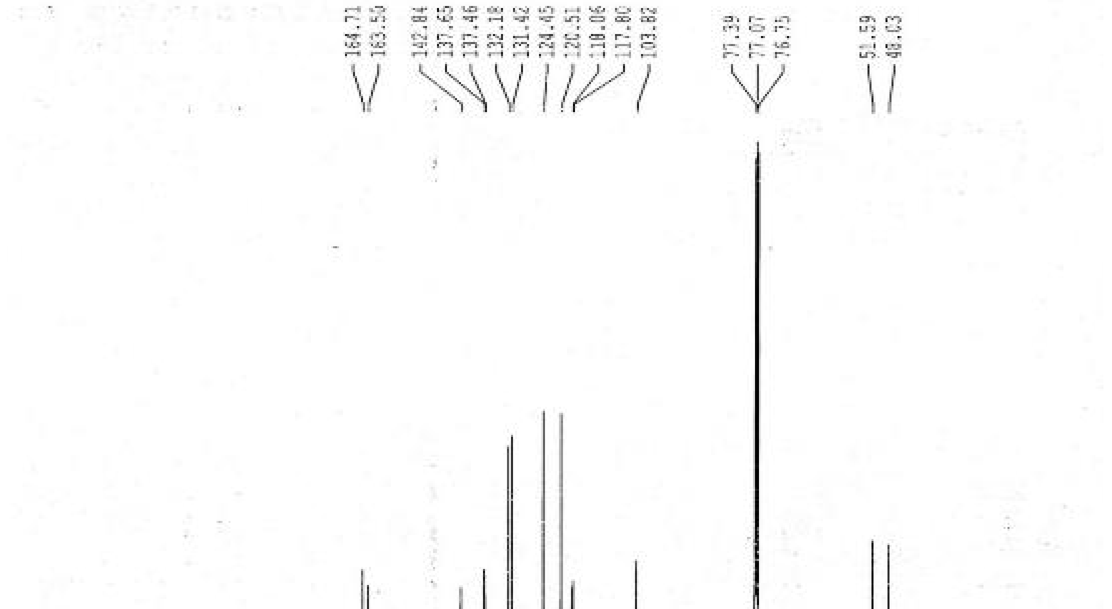

The ^{13}C NMR spectrum of methyl-1-(4-bromophenyl)-4-((4-bromophenyl)amino)-5-oxo-2,5-dihydro-1H-pyrrole-3-carboxylate

Ethyl-1-(4-bromophenyl)-4-((4-bromophenyl)amino)-5-oxo-2,5-dihydro-1H-pyrrole-3-carboxylate

White solid. M. p. 165-166 °C . FT-IR (KBr)/ ῡ(cm^{-1}): 3283, 1697, 1638, 1587, 1490, 1384, 1258, 1165, 819.;^{1}H NMR ($CDCl_3$, 400 MHz)/δ ppm: 8.03 (br, s, 1H, NH), 7.69 (d, 2H, ^{3}J= 8.4 Hz, Ar–H), 7.50 (d, 2H, ^{3}J= 8.4 Hz, Ar–H), 7.42 (d, 2H, ^{3}J= 8.0 Hz, Ar–H), 7.01 (d, 2H, ^{3}J= 8.0 Hz, Ar–H), 4.50 (s, 2H, NCH_2), 4.24 (s, 2H, OCH_2CH_3), 1.27 (s, 3H, OCH_2CH_3). ^{13}C NMR ($CDCl_3$, 100 MHz)/δ ppm: 165.1, 163.1, 140.3, 139.8, 138.4, 132.3 (2C), 131.0 (2C), 123.0 (2C), 121.5 (2C), 117.1, 114.5, 106.9, 60.3, 48.8, 14.2.

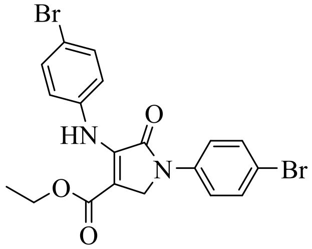

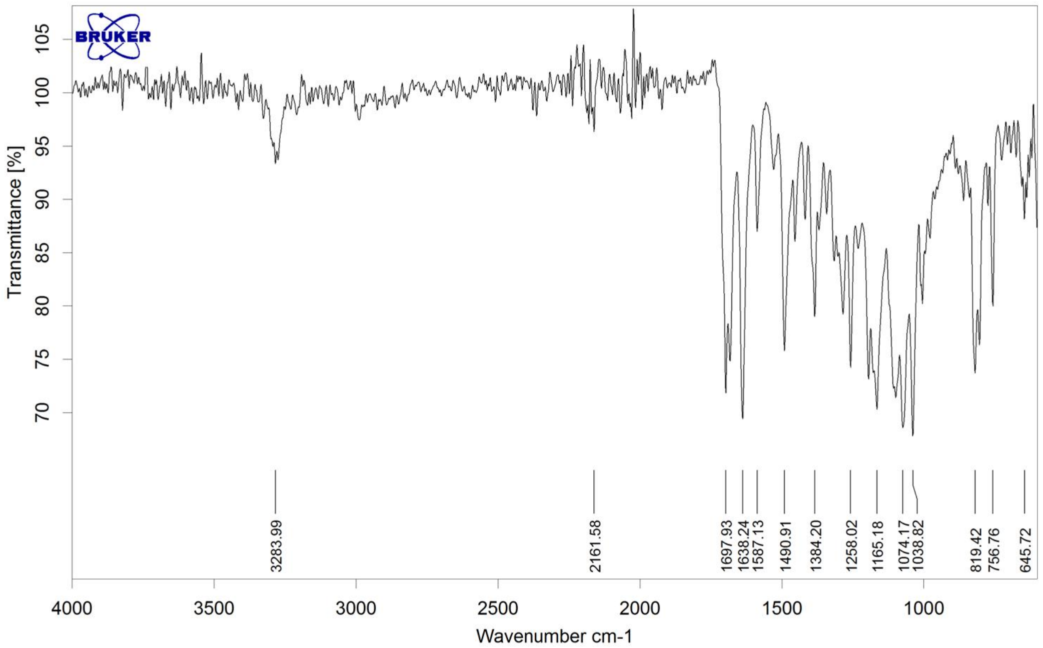

The FT-IR of ethyl-1-(4-bromophenyl)-4-((4-bromophenyl)amino)-5-oxo-2,5-dihydro-1H-pyrrole-3-carboxylate

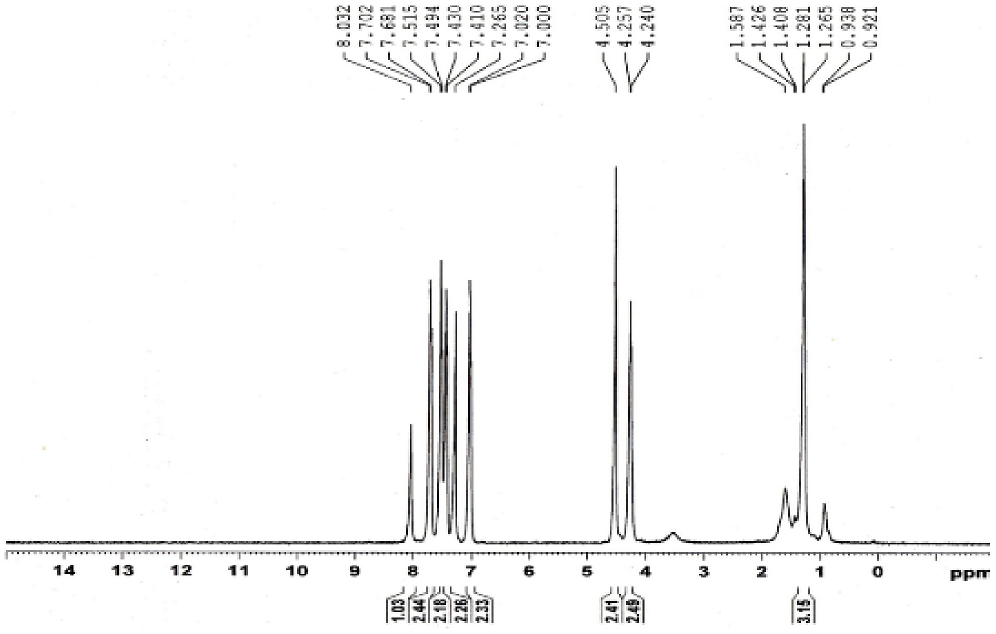

The ^{1}H NMR spectrum of ethyl-1-(4-bromophenyl)-4-((4-bromophenyl)amino)-5-oxo-2,5-dihydro-1H-pyrrole-3-carboxylate

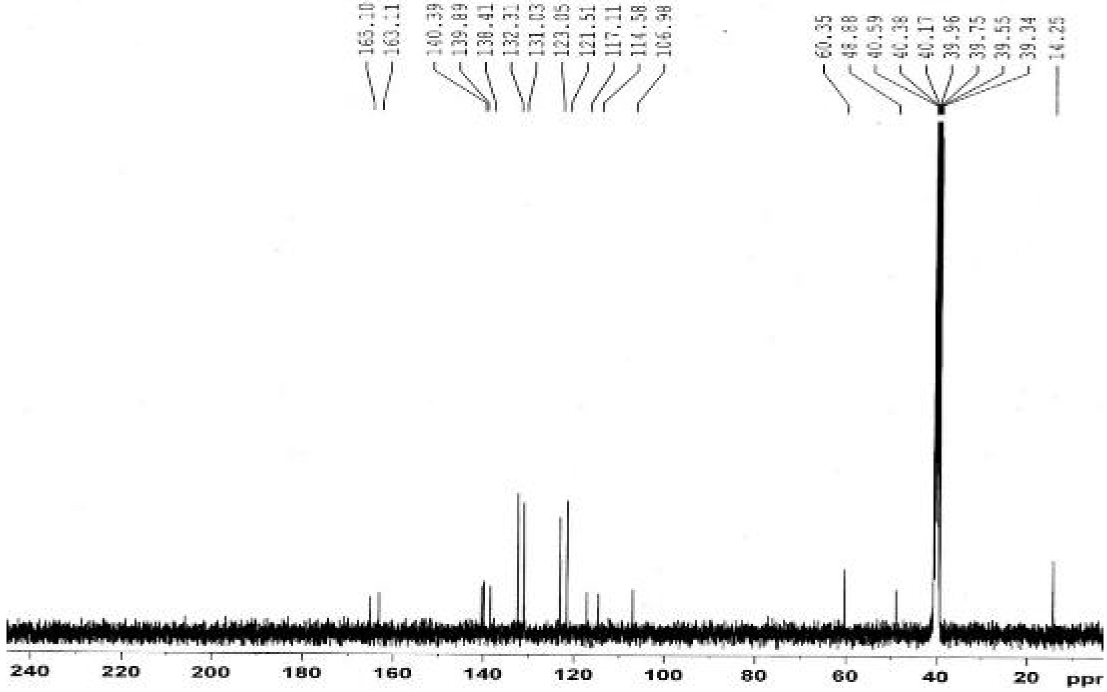

The ^{13}C NMR spectrum of ethyl-1-(4-bromophenyl)-4-((4-bromophenyl)amino)-5-oxo-2,5-dihydro-1H-pyrrole-3-carboxylate

Ethyl-1-(4-Nitrophenyl)-4-((4-Nitrophenyl)amino)-5-oxo-2,5-dihydro-1H-pyrrole-3-carboxylate

Yellow solid. M. p. 206-208 °C. FT-IR (KBr)/ ῡ(cm^{-1}): 3313, 1710, 1677, 1593, 1510, 1306, 1267, 1185, 1104, 844.;^{1}H NMR ($CDCl_3$, 400 MHz)/δ ppm: 8.35 (s, 1H, NH), 8.30 (d 2H, ^{3}J= 8.4 Hz, Ar–H), 8.21 (d, 2H, ^{3}J= 8.8 Hz, Ar–H), 8.01 (d, 2H, ^{3}J= 8.8 Hz, Ar–H), 7.19 (d, 2H, ^{3}J= 8.4 Hz, Ar–H), 4.65 (s, 2H, NCH_2), 4.32 (s, 2H, OCH_2CH_3), 1.32 (t, 3H, OCH_2CH_3).;^{13}C NMR ($CDCl_3$, 100 MHz)/δ ppm: 165.1, 162.5, 146.8, 144.1, 143.7, 141.9, 139.3, 125.0 (2C), 124.1 (2C), 119.6 (2C), 118.5 (2C), 111.9, 60.8, 48.8, 14.2.; MS (*m/z*): 412.1 (M+), 339.2, 219.1, 190.1, 174.1, 163.1, 150.1, 129.1, 117.1, 103.0, 92, 76 (100%), 65, 51.1.29; Anal. Calcd for $C_{18}H_{14}N_4O_7$, C, 54.28; H, 3.54; N, 14.07; O, 28.12; foundC, 55.11; H, 3.14; N, 13.64.

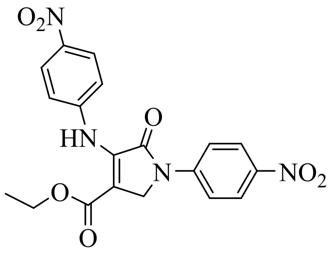

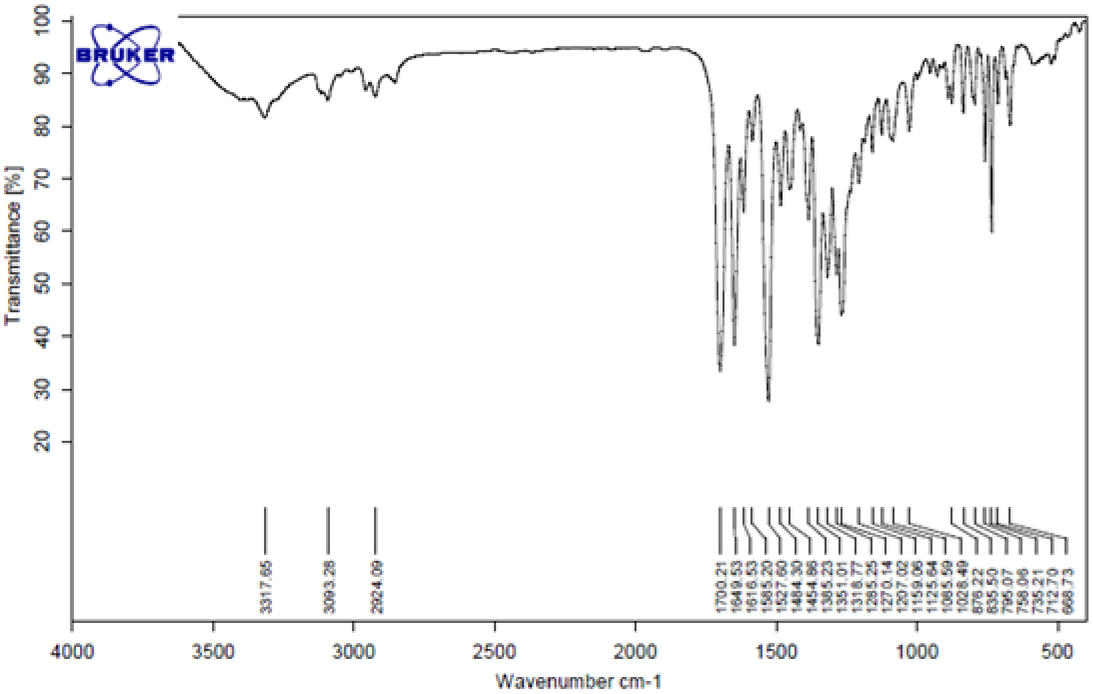

The FT-IR of ethyl-1-(4-Nitrophenyl)-4-((4-Nitrophenyl)amino)-5-oxo-2,5-dihydro-1H-pyrrole-3-carboxylate

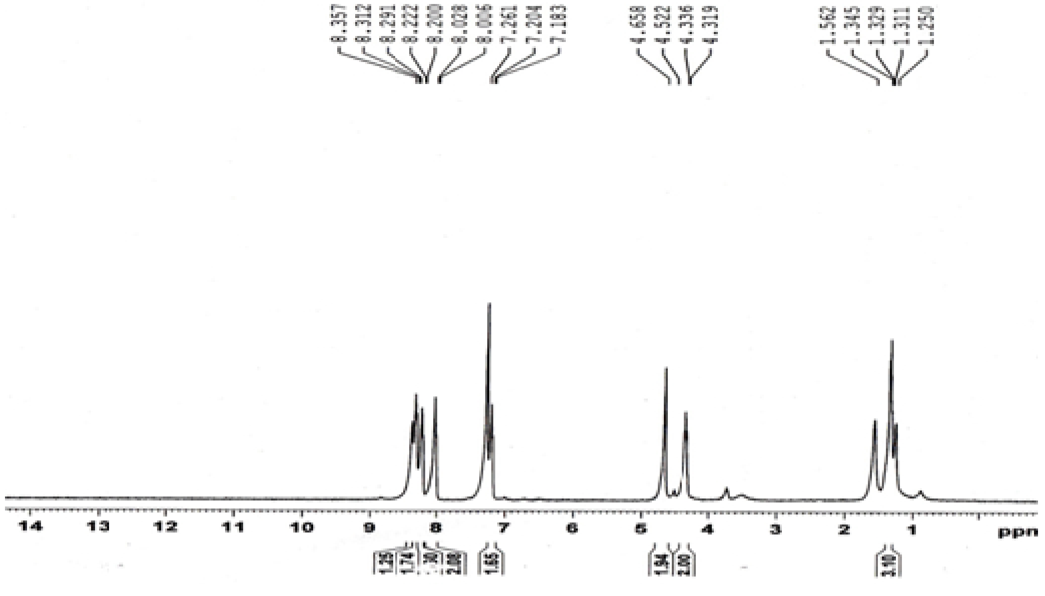

The ^{1}H NMR spectrum of ethyl-1-(4-Nitrophenyl)-4-((4-Nitrophenyl)amino)-5-oxo-2,5-dihydro-1H-pyrrole-3-carboxylate

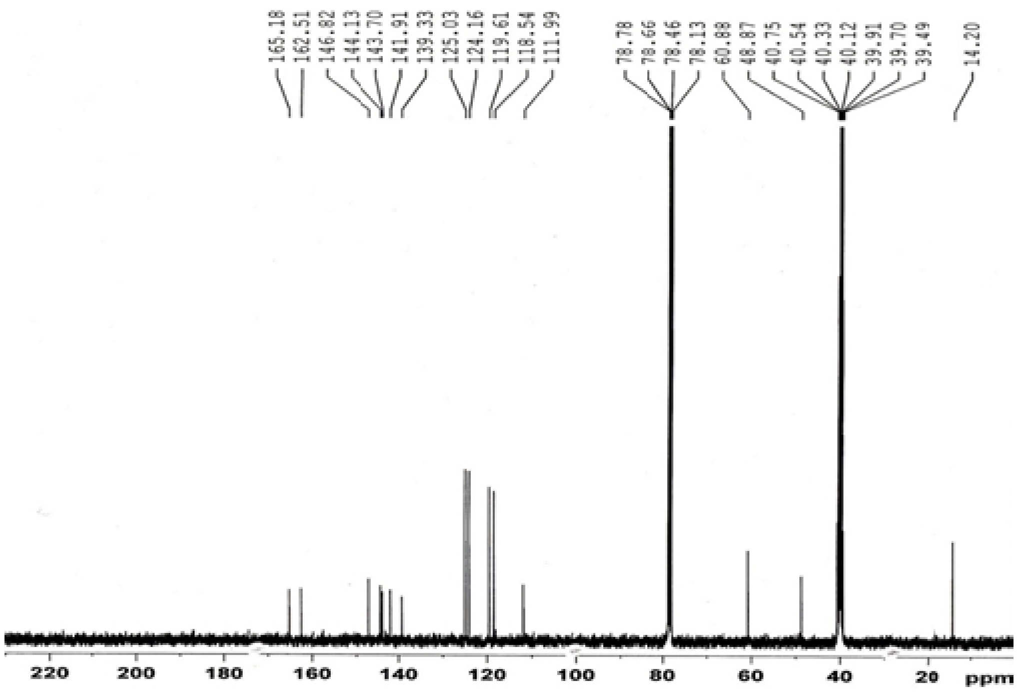

The ^{13}C NMR spectrum of ethyl-1-(4-Nitrophenyl)-4-((4-Nitrophenyl)amino)-5-oxo-2,5-dihydro-1H-pyrrole-3-carboxylate

Methyl-1-(3-Nitrophenyl)-4-((3-Nitrophenyl)amino)-5-oxo-2,5-dihydro-1H-pyrrole-3-carboxylate

white solid. M. p. 202-204 °C. FT-IR (KBr)/ ῡ(cm^{-1}): 3338, 1696, 1645, 1615, 1625, 1483, 1349, 1318, 1284, 733. ^{1}H NMR ($CDCl_3$, 400 MHz)/δ ppm: 8.52 (br, s, 1H, NH), 8.32 (br, s, 1H, Ar–H), 8.26 (br, s, 1H, Ar–H), 8.03 (br, s, 3H, Ar–H), 7.59 (d, 1H, ^{3}J= 7.2 Hz, Ar–H), 7.48 (d, 2H, ^{3}J= 7.6 Hz, Ar–H), 4.65 (s, 2H, NCH_2), 3.85 (s, 3H, OCH_3).;^{13}C NMR ($CDCl_3$, 100 MHz)/δ ppm: 165.2, 162.5, 149.6, 149.0, 143.1, 141.0, 140.8, 132.1, 130.6, 128.4, 126.4, 120.7, 118.4, 116.2, 114.9, 110.6, 53.0, 50.1.; MS (*m/z*): 398.3 (M+), 339.3, 218.2, 190.2, 174.1, 164.1, 150.1, 138.1, 128.1, 116.1, 103.1, 92, 76 (100%), 65, 51; Anal. Calcd for $C_{18}H_{14}N_4O_7$ C, 54.28; H, 3.54; N, 14.07; found C, 53.88; H, 3.74; N, 14.27.

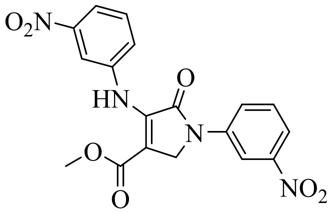

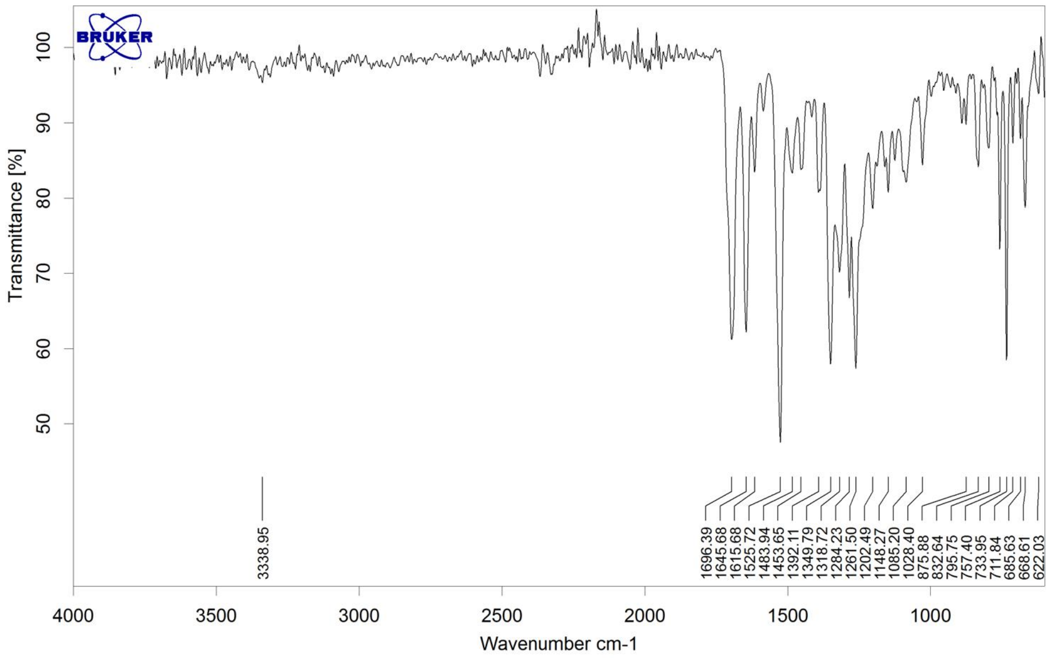

The FT-IR of Methyl-1-(3-Nitrophenyl)-4-((3-Nitrophenyl)amino)-5-oxo-2,5-dihydro-1H-pyrrole-3-carboxylate

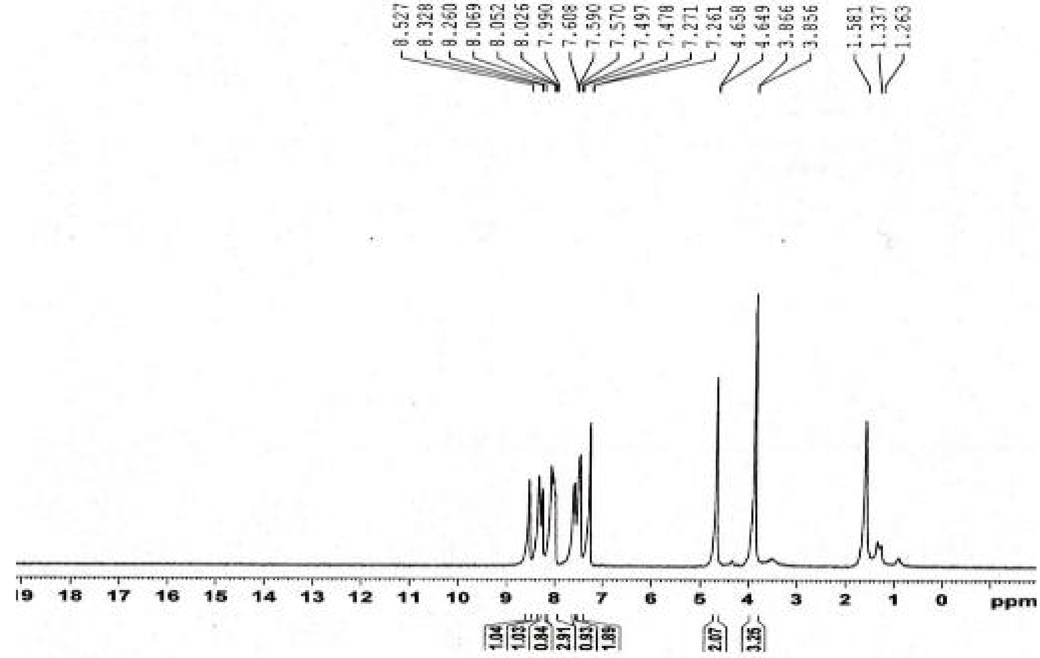

The ^{1}H NMR spectrum of Methyl-1-(3-Nitrophenyl)-4-((3-Nitrophenyl)amino)-5-oxo-2,5-dihydro-1H-pyrrole-3-carboxylate

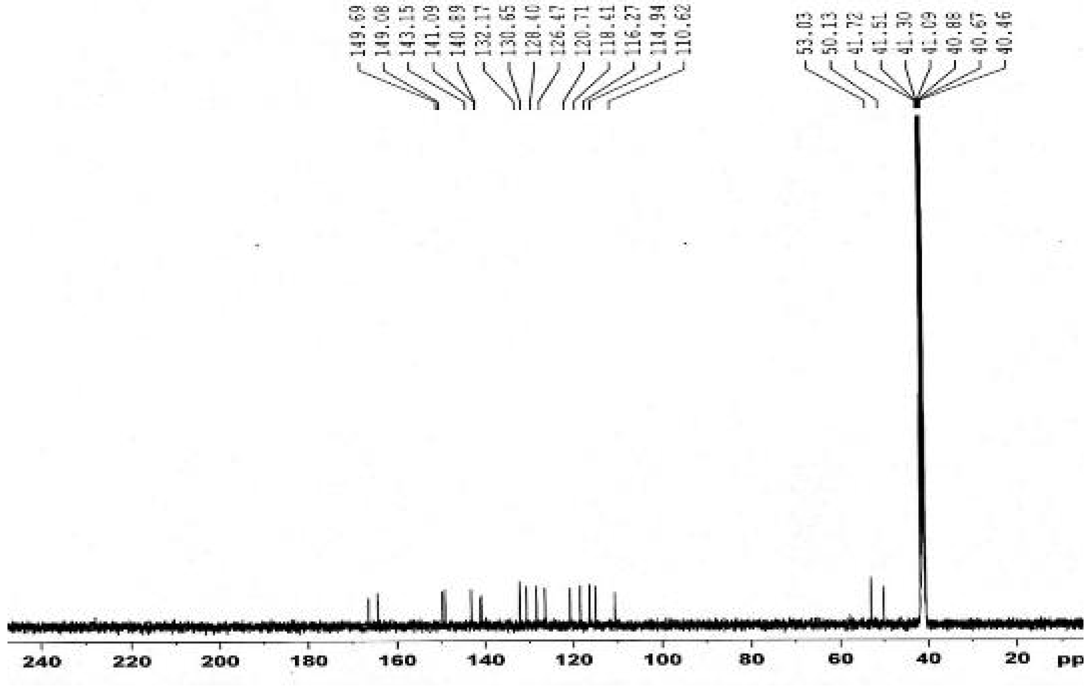

The ^{13}C NMR spectrum of Methyl-1-(3-Nitrophenyl)-4-((3-Nitrophenyl)amino)-5-oxo-2,5-dihydro-1H-pyrrole-3-carboxylate

Ethyl-1-(3-Nitrophenyl)-4-((3-Nitrophenyl)amino)-5-oxo-2,5-dihydro-1H-pyrrole-3-carboxylate

white solid. M.p. 190-192 °C. FT-IR (KBr)/ ῡ(cm^{-1}): 3301, 2923, 1701, 1687, 1648, 1524, 1480, 1455, 1349, 1204, 1035, 735.;^{1}H NMR ($CDCl_3$, 400 MHz)/δ ppm: 8.52 (s, 1H, NH), 8.35 (br, s, 1H, Ar–H), 8.26 (s, 1H, Ar–H), 8.06 (d, 1H, ^{3}J= 6.4 Hz, Ar–H), 7.99 (br, s, 2H, Ar–H), 7.59 (br, s, 1H, Ar–H), 7.47 (br, s, 2H, Ar–H), 4.65 (s, 2H, NCH_2), 4.33 (s, 2H, OCH_2CH_3), 1.34 (s, 3H, OCH_2CH_3).;^{13}C NMR ($CDCl_3$, 100 MHz)/δ ppm: 165.4, 162.8, 148.5, 148.0, 142.3, 139.9, 139.3, 131.0, 129.5 (2C), 126.9 (2C), 125.3 (2C), 119.5 (2C), 117.1, 114.8, 113.8, 109.7, 60.6, 49.1, 14.3.; MS (*m/z*): 412.3 (M+), 339.1, 219.2, 190.1, 174.1, 163.1, 150.1 (100%), 139.1, 128.1, 116.1, 103.1, 92, 76, 65, 51; Anal. Calcd for $C_{19}H_{16}N_4O_7$ C, 55.34; H, 3.91; N, 13.59; foundC, 54.99, H, 4.21; N, 13.64.

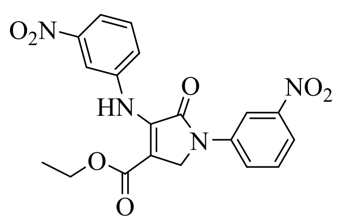

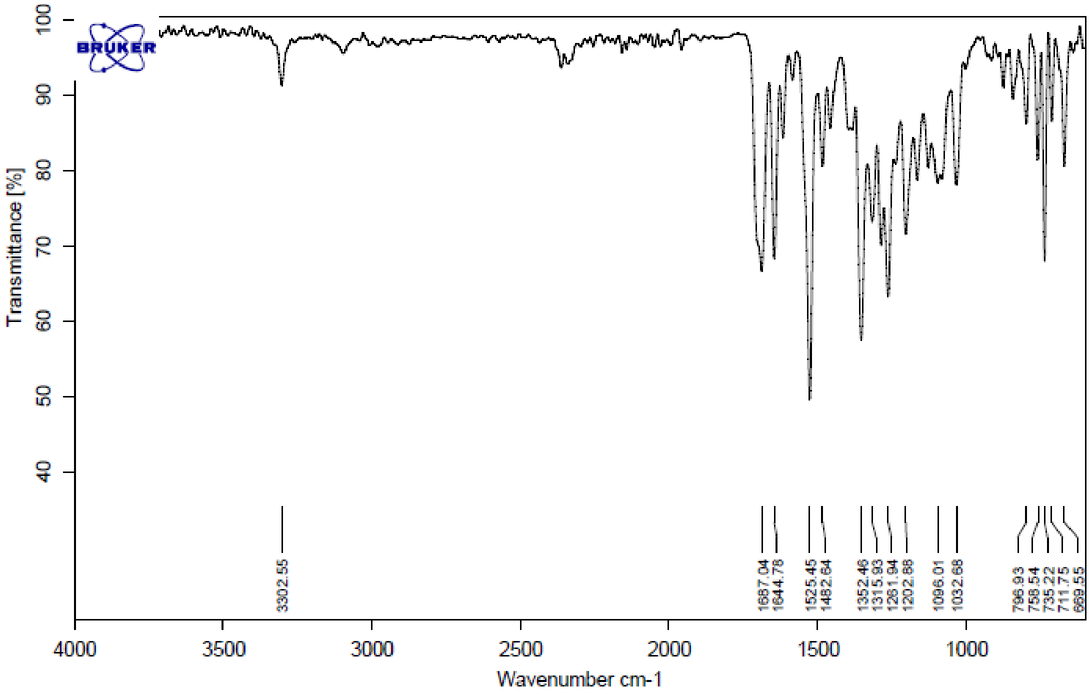

The FT-IR of Ethyl-1-(3-Nitrophenyl)-4-((3-Nitrophenyl)amino)-5-oxo-2,5-dihydro-1H-pyrrole-3-carboxylate

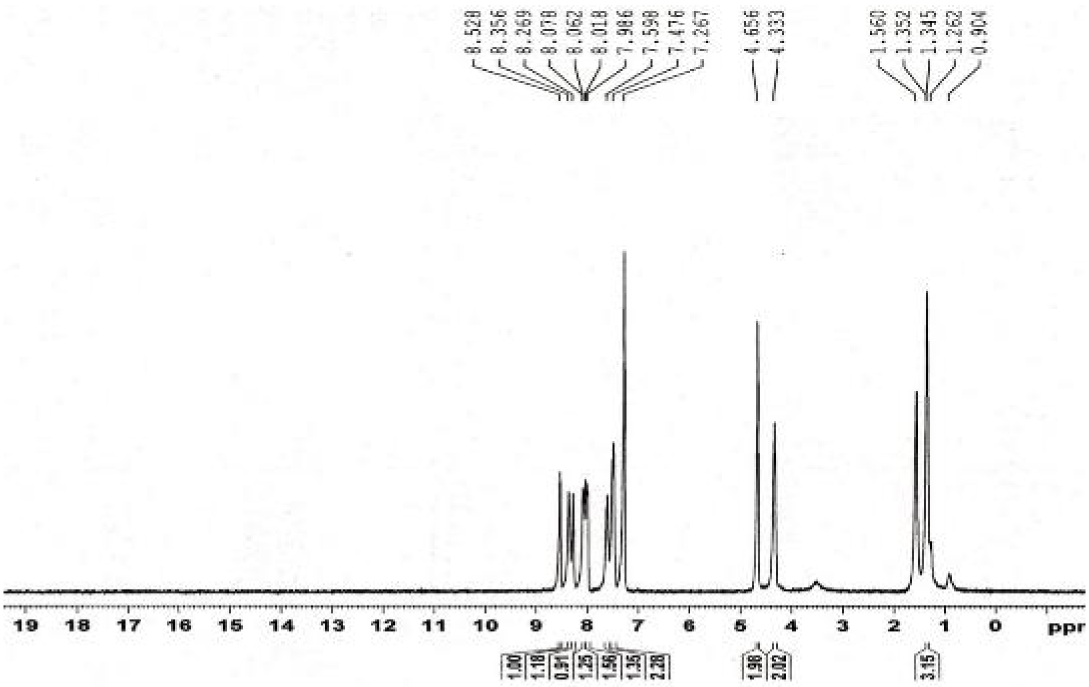

The ^{1}H NMR spectrum of Ethyl-1-(3-Nitrophenyl)-4-((3-Nitrophenyl)amino)-5-oxo-2,5-dihydro-1H-pyrrole-3-carboxylate

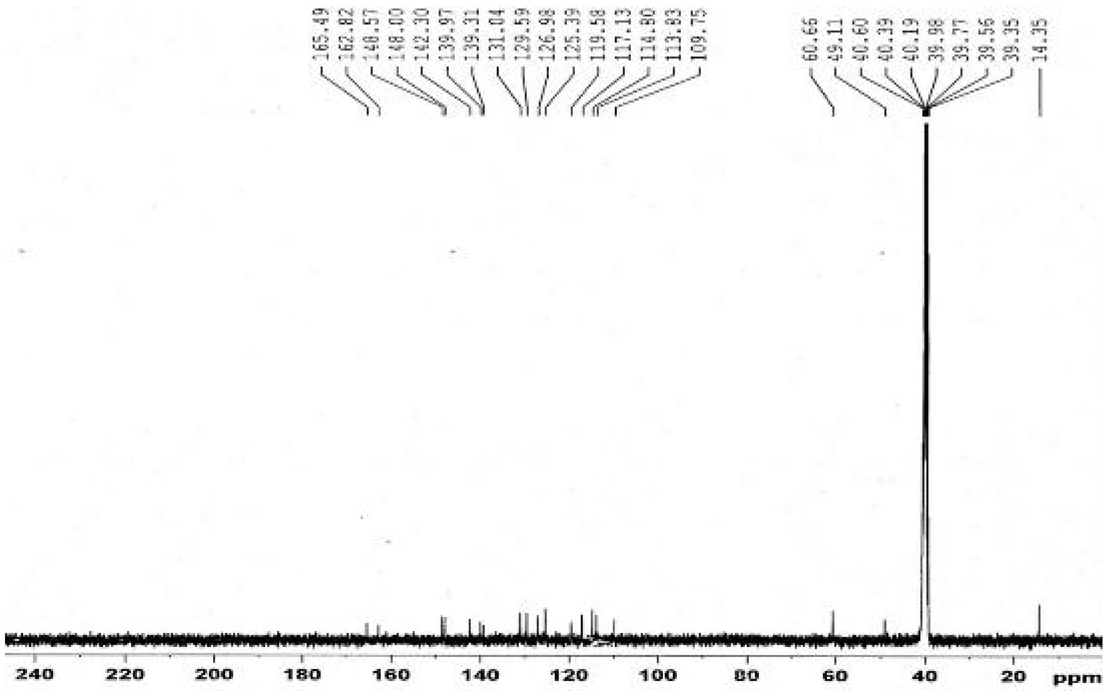

The ^{13}C NMR spectrum of Ethyl-1-(3-Nitrophenyl)-4-((3-Nitrophenyl)amino)-5-oxo-2,5-dihydro-1H-pyrrole-3-carboxylate

Methyl-1-(*p*-tolyl)-4-((*p*-tolyl)amino)-5-oxo-2,5-dihydro-1H-pyrrole-3-carboxylate

Pale yellow solid. M. p. 174-176 ˚C. FT-IR (KBr)/ ῡ(cm^{-1}): 3284, 1672, 1646, 1512, 1440, 1398, 1225, 1151. ^{1}H NMR ($CDCl_3$, 400 MHz)/δ ppm: 8.00 (br, s, 1H, NH), 7.66 (br, s, 2H, Ar–H), 7.19 (br, s, 2H, Ar–H), 7.12 (br, s, 2H, Ar–H), 7.04 (br, s, 2H, Ar–H), 4.50 (s, 2H, NCH_2), 3.75 (s, 3H, OCH_3),2.34 (s, 6H, Ar-Me). ^{13}C NMR ($CDCl_3$, 100 MHz)/δ ppm: 164.9, 163.5, 143.5, 136.2, 135.9, 134.7, 134.4, 129.6 (2C), 128.9 (2C), 123.1 (2C), 119.2 (2C), 101.8, 51.2, 48.2, 21.0, 20.8.

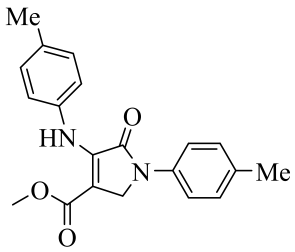

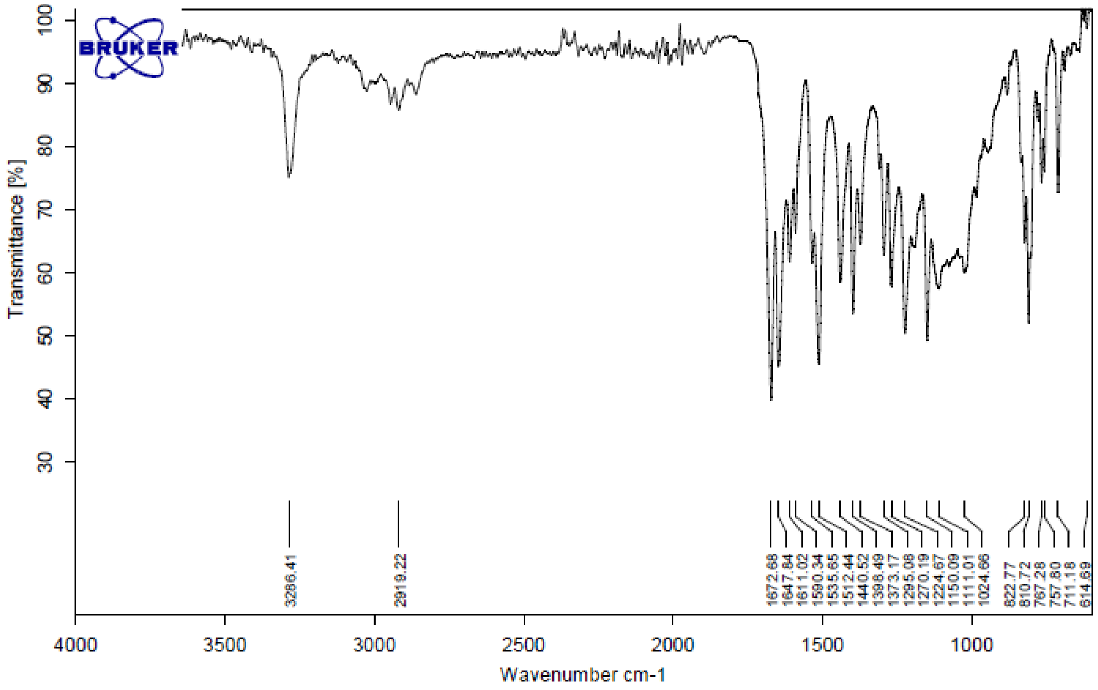

The FT-IR of Methyl-1-(*p*-tolyl)-4-((*p*-tolyl)amino)-5-oxo-2,5-dihydro-1H-pyrrole-3-carboxylate

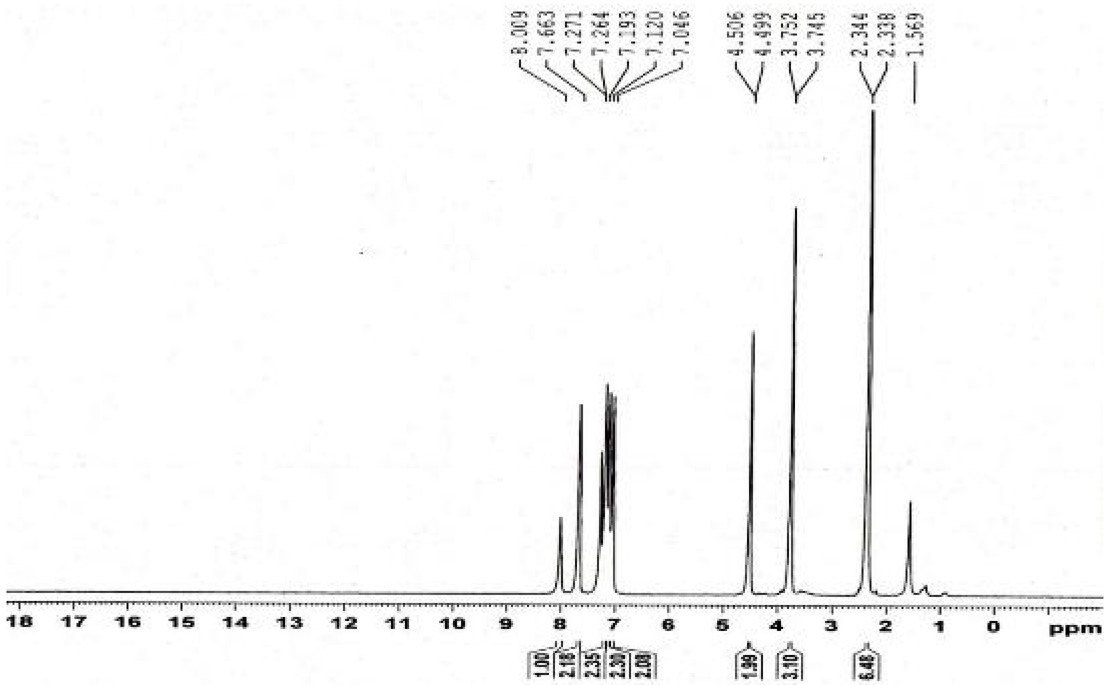

The ^{1}H NMR spectrum of Methyl-1-(*p*-tolyl)-4-((*p*-tolyl)amino)-5-oxo-2,5-dihydro-1H-pyrrole-3-carboxylate

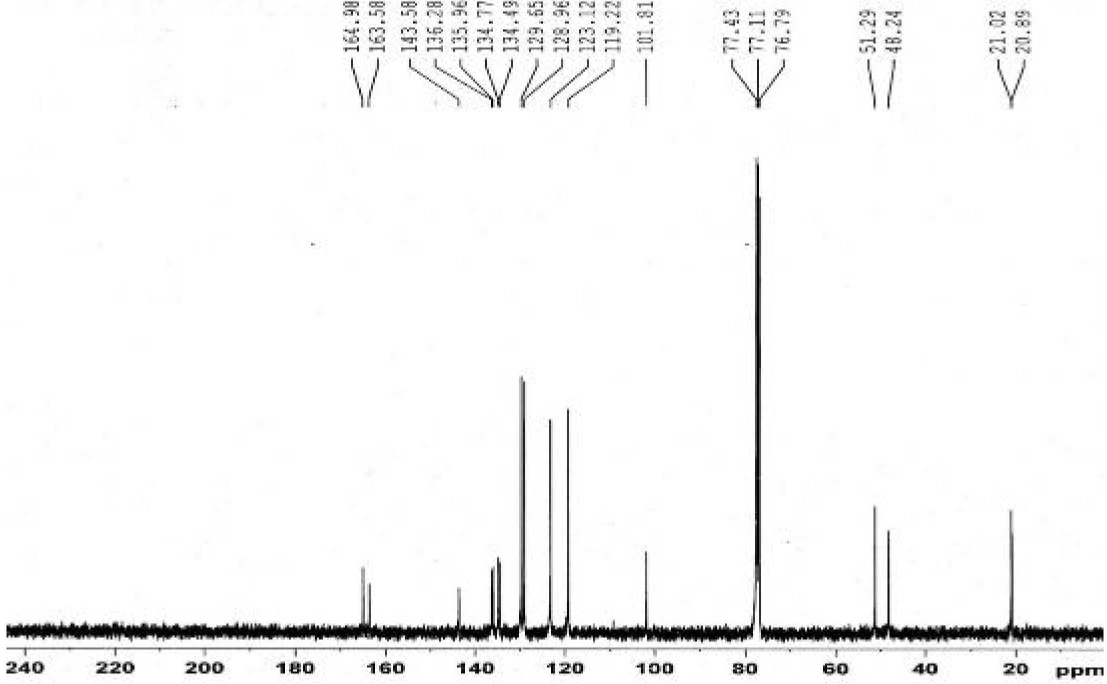

The ^{13}C NMR spectrum of Methyl-1-(*p*-tolyl)-4-((*p*-tolyl)amino)-5-oxo-2,5-dihydro-1H-pyrrole-3-carboxylate

Methyl-1-(4-ethylphenyl)-4-((4-ethylphenyl)amino)-5-oxo-2,5-dihydro-1H-pyrrole-3-carboxylate

Cream solid. M. p.125-126 °C. FT-IR (KBr)/ ῡ(cm^{-1}): 3292, 2963, 1673, 1644, 1609, 1513, 1436, 1401, 1274, 1222, 1148. ^{1}H NMR ($CDCl_3$, 400 MHz)/δ ppm: 8.03 (br, s, 1H, NH), 7.69 (br, s, 2H, Ar–H), 7.22 (br, s, 2H, Ar–H), 7.14 (br, s, 2H, Ar–H), 7.07 (br, s, 2H, Ar–H), 4.51 (s, 2H, NCH_2), 3.73 (s, 3H, OCH_3), 2.65 (s, 4H, Ar-CH_2CH_3), 1.24 (t, 3*J*= 5.2 Hz, 6H, Ar-CH_2CH_3)

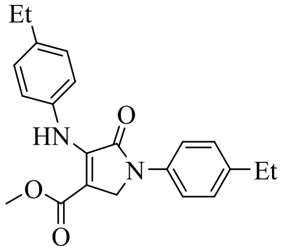

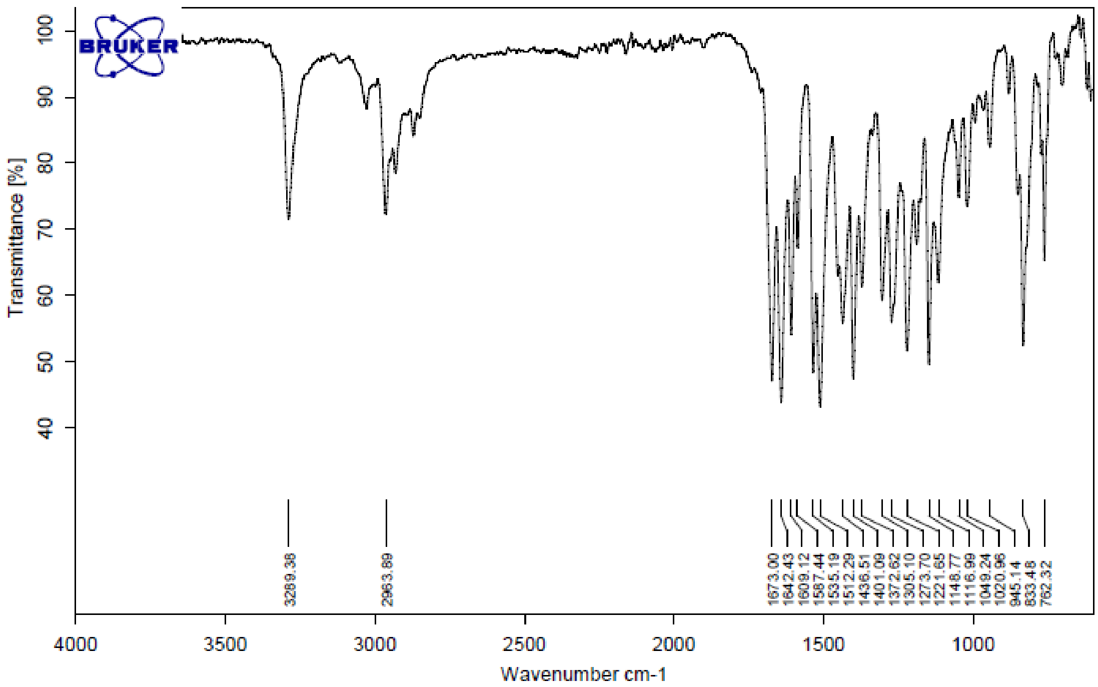

The FT-IR of Methyl-1-(4-ethylphenyl)-4-((4-ethylphenyl)amino)-5-oxo-2,5-dihydro-1H-pyrrole-3-carboxylate

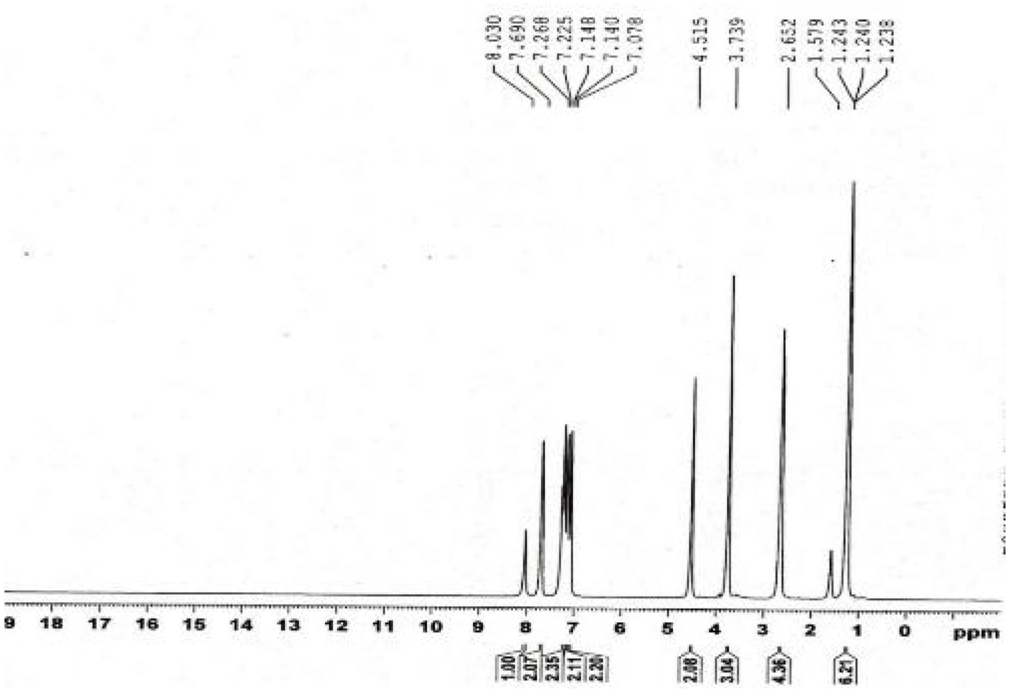

The ^{1}H NMR spectrum of Methyl-1-(4-ethylphenyl)-4-((4-ethylphenyl)amino)-5-oxo-2,5-dihydro-1H-pyrrole-3-carboxylate

Methyl-1-(4-methoxyphenyl)-4-((4-methoxyphenyl)amino)-5-oxo-2,5-dihydro-1H-pyrrole-3-carboxylate

White solid. M. p.160-162 °C. FT-IR (KBr)/ ῡ(cm-1): 3280, 1642, 1508, 1509, 1443, 1402, 1270, 1179, 1151, 1030. ^{1}H NMR ($CDCl_3$, 400 MHz)/δ ppm: 8.00 (br, s, 1H, NH), 7.64 (d, 2H, ^3J= 8.8 Hz, Ar–H), 7.08 (d, 2H, ^3J= 8.4Hz, Ar–H), 6.89 (d, 2H, ^3J= 8.4 Hz, Ar–H), 6.84 (d, 2H, ^3J= 8.4 Hz, Ar–H), 4.46 (br, s, 2H, NCH_2), 3.79 (s, 6H, $ArOCH_3$), 3.73 (s, 3H, OCH_3).;^{13}C NMR ($CDCl_3$, 100 MHz)/δ ppm: 165.1, 163.3, 157.1, 156.9, 144.0, 131.9, 131.5, 125.0 (2C), 121.1 (2C), 114.2 (2C), 113.6 (2C), 100.8, 55.5, 55.4, 51.2, 48.4.

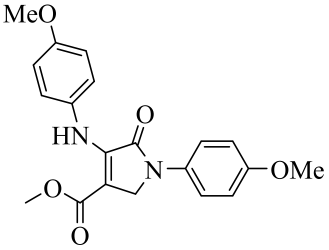

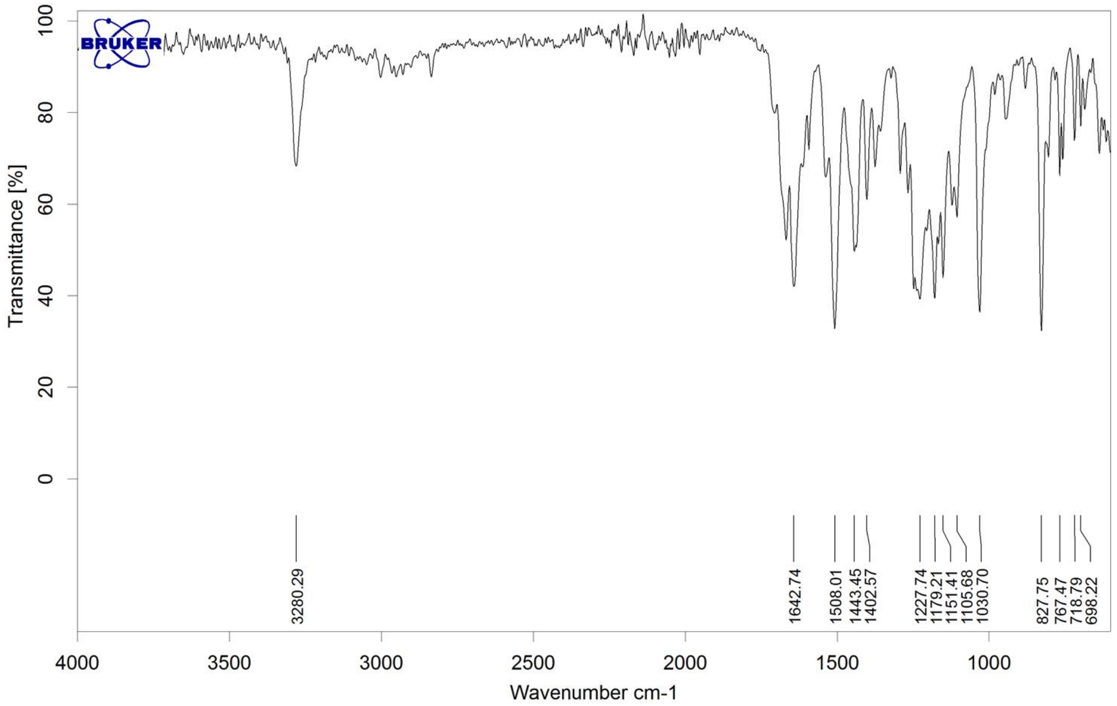

The FT-IR of Methyl-1-(4-methoxyphenyl)-4-((4-methoxyphenyl)amino)-5-oxo-2,5-dihydro-1H-pyrrole-3-carboxylate.

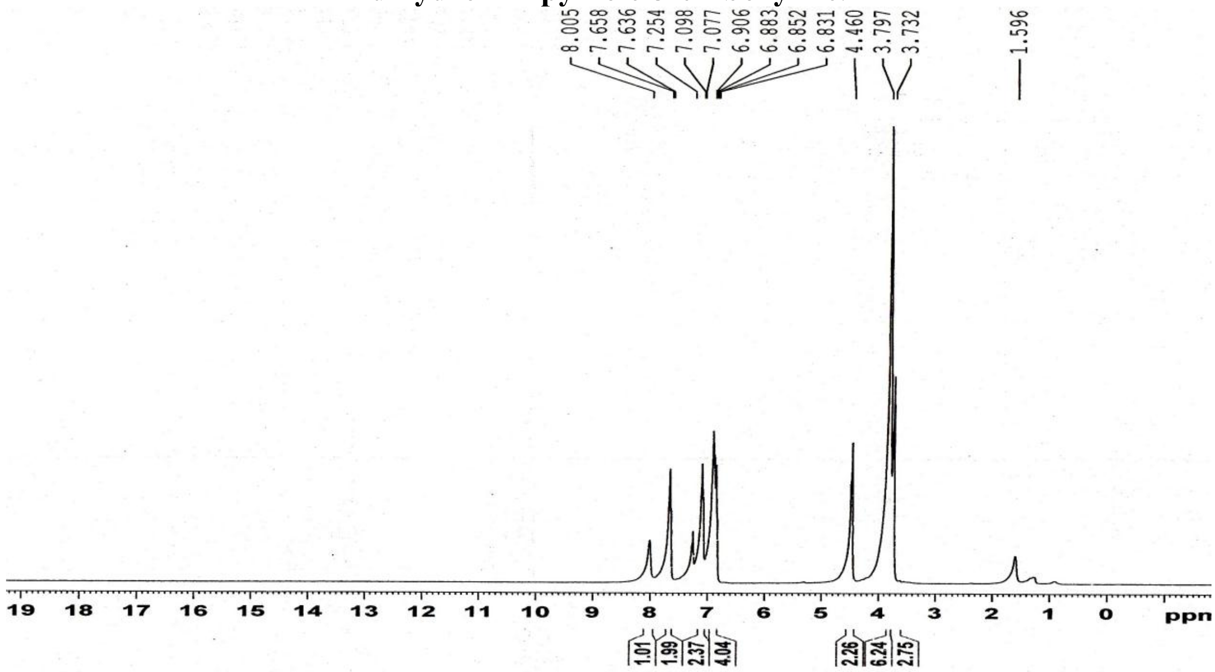

The ^{1}H NMR(400MHz) spectrum of Methyl-1-(4-methoxyphenyl)-4-((4-methoxyphenyl)amino)-5-oxo-2,5-dihydro-1H-pyrrole-3-carboxylate

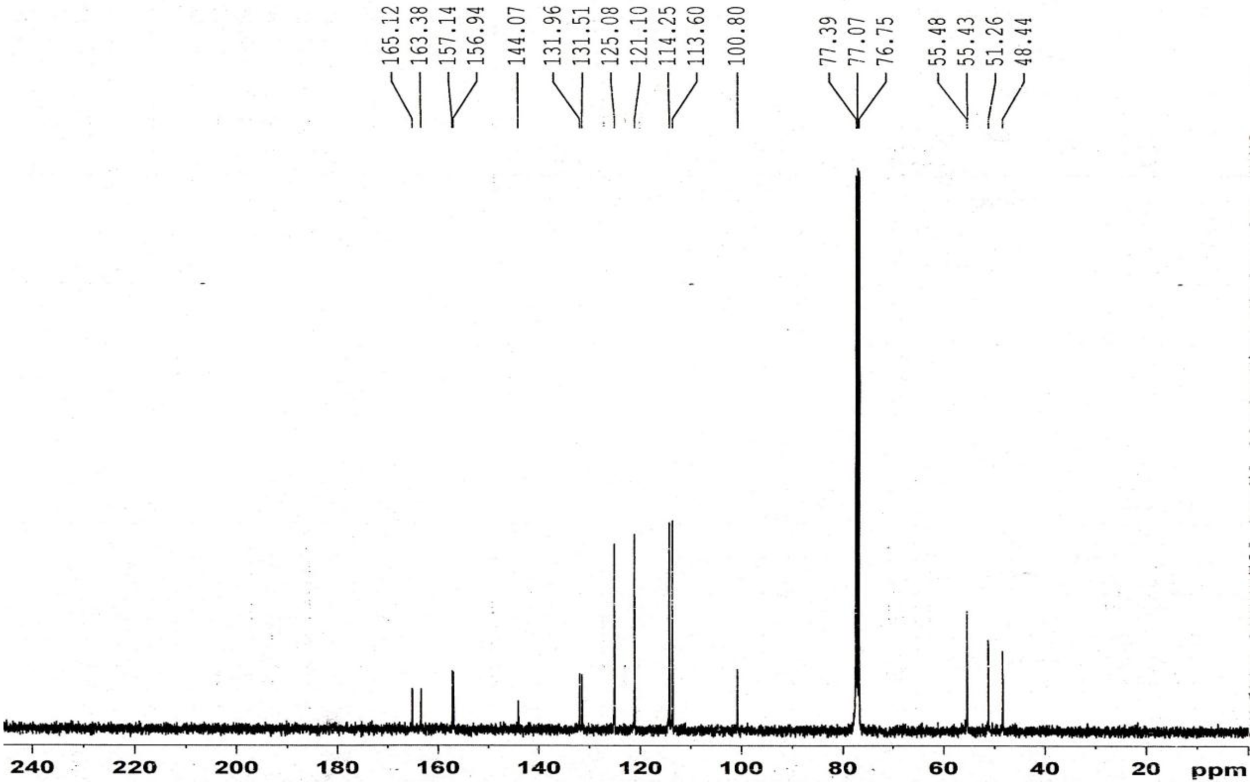

The ^{13}C NMR(100MHz) spectrum of Methyl-1-(4-methoxyphenyl)-4-((4-methoxyphenyl)amino)-5-oxo-2,5-dihydro-1H-pyrrole-3-carboxylate

Ethyl-1-(4-methoxyphenyl)-4-((4-methoxyphenyl)amino)-5-oxo-2,5-dihydro-1H-pyrrole-3-carboxylate

White solid. M. p. 152-154 °C. FT-IR (KBr)/ ῡ(cm^{-1}): 3263, 1690, 1639, 1509, 1465, 1439, 1373, 1244, 1173, 1105, 1039, 819. ^{1}H NMR ($CDCl_3$, 400 MHz)/δ ppm: 8.00 (br, s, 1H, NH), 7.65 (br, s, 2H, Ar–H), 7.09 (br, s, 2H, Ar–H), 6.90 (br, s, 2H, Ar–H), 6.84 (br, s, 2H, Ar–H), 4.47 (s, 2H, NCH2), 4.20 (s, 2H, OCH_2CH_3), 3.80 (s, 6H, Ar-OMe), 1.24 (s, 3H, OCH_2CH_3). ^{13}C NMR ($CDCl_3$,

100 MHz)/δ ppm: 164.8, 163.5, 157.0, 156.9, 131.9, 131.6, 124.8 (2C), 121.1 (2C), 114.2 (2C), 113.6 (2C), 101.3, 60.1, 55.4, 55.4, 48.5, 14.3.

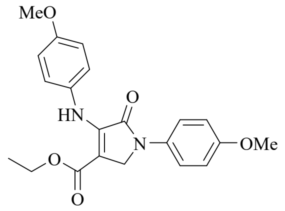

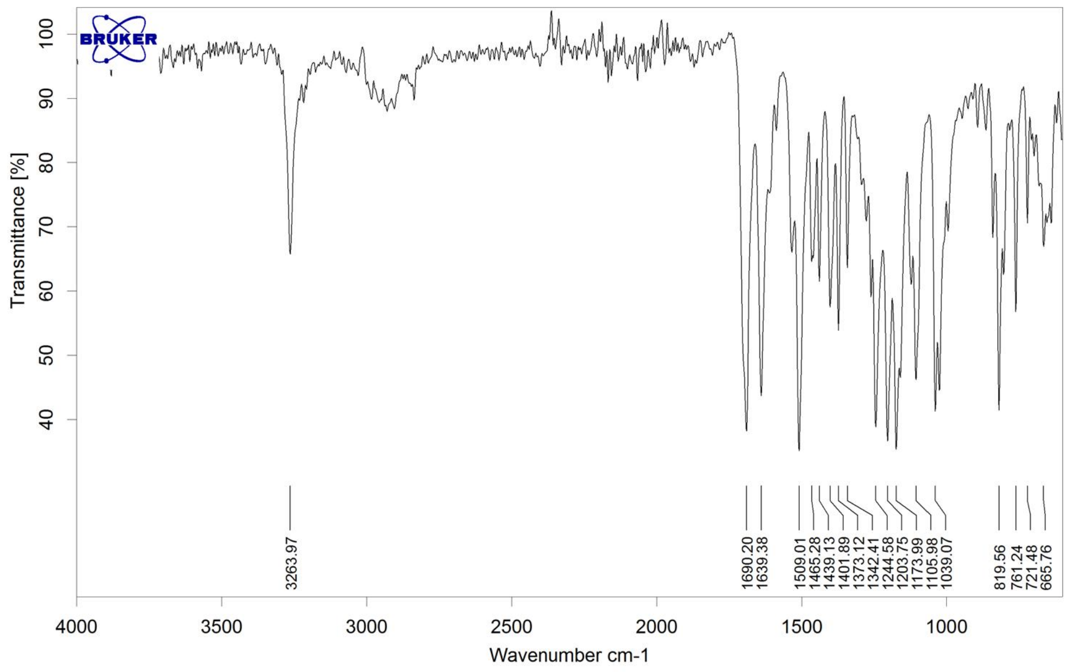

The FT-IR of Ethyl-1-(4-methoxyphenyl)-4-((4-methoxyphenyl)amino)-5-oxo-2,5-dihydro-1H-pyrrole-3-carboxylate

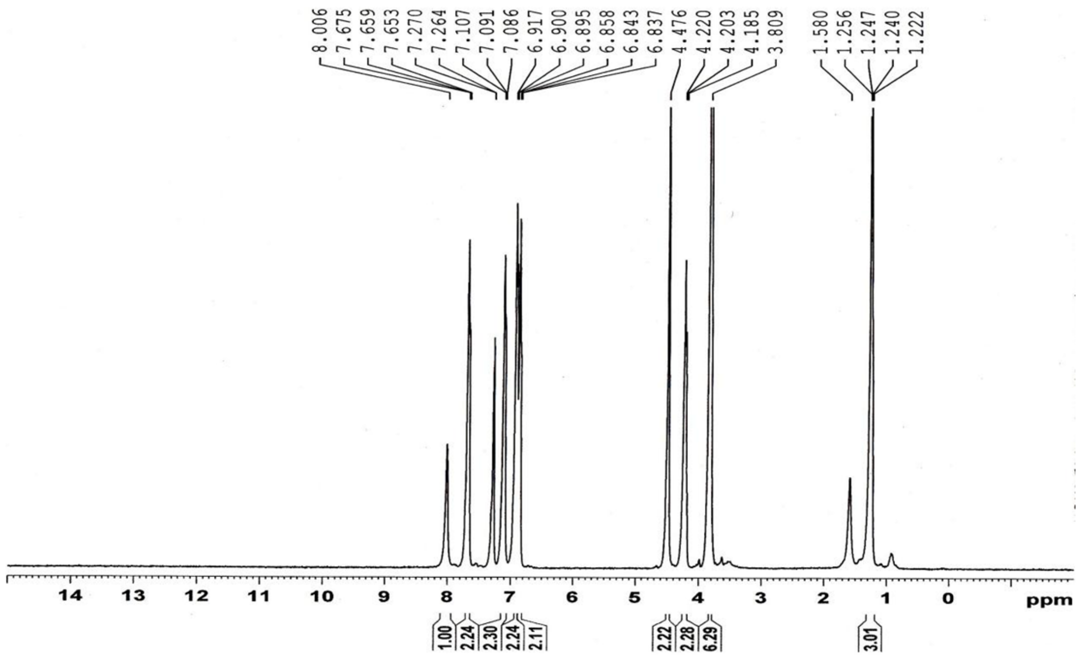

The ^{1}H NMR (400MHz) spectrum of Ethyl-1-(4-methoxyphenyl)-4-((4-methoxyphenyl)amino)-5-oxo-2,5-dihydro-1H-pyrrole-3-carboxylate

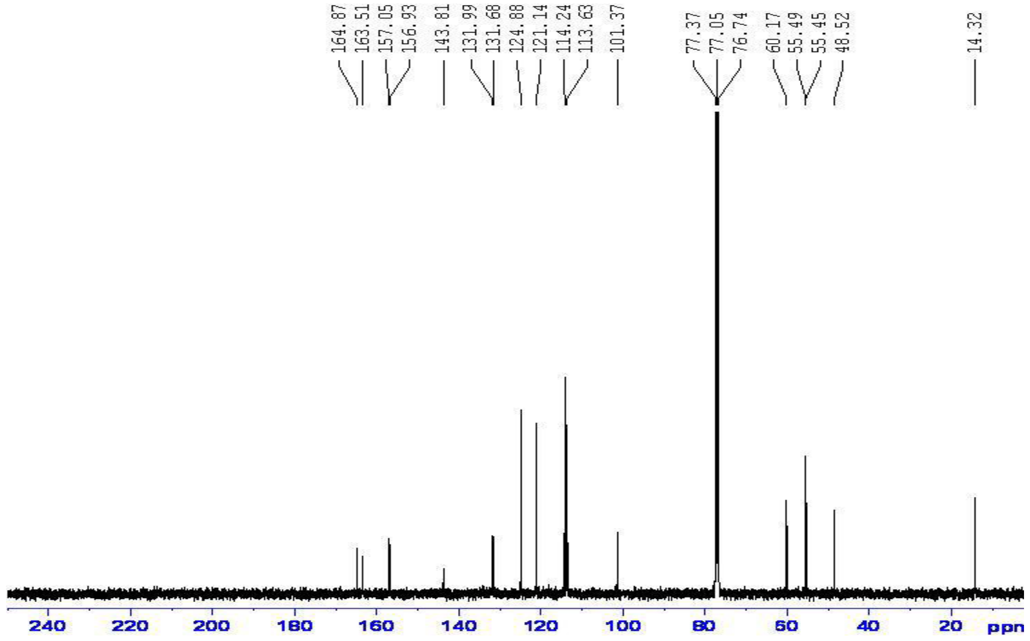

The ^{13}C NMR (100 MHz) spectrum of Ethyl-1-(4-methoxyphenyl)-4-((4-methoxyphenyl)amino)-5-oxo-2,5-dihydro-1H-pyrrole-3-carboxylate
